# Prevalence and risk factors of cognitive impairment in Chinese patients with hypertension: a systematic review and meta-analysis

**DOI:** 10.3389/fneur.2023.1271437

**Published:** 2024-02-13

**Authors:** Cheng Xie, Dongling Zhong, Yue Zhang, Xiaobo Liu, Lili Zhang, Xiao Luo, Yimeng Gong, Wei Jiang, Rongjiang Jin, Juan Li

**Affiliations:** ^1^School of Health Preservation and Rehabilitation, Chengdu University of Traditional Chinese Medicine, Chengdu, China; ^2^First Teaching Hospital of Tianjin University of Traditional Chinese Medicine, Tianjin, China; ^3^Department of Orthopedics, Pingshan County Hospital of Traditional Chinese Medicine, Yibing, China; ^4^Affiliated Sichuan Provincial Rehabilitation Hospital of Chengdu University of Traditional Chinese Medicine and Sichuan Provincial BAYI Rehabilitation Center, Chengdu, China

**Keywords:** cognitive impairment, hypertension, prevalence, risk factors, China, meta

## Abstract

**Background:**

Cognitive impairment is prevalent in Chinese patients with hypertension; however, current evidence on prevalence and risk factors is required to be synthesized.

**Objectives:**

This systematic review and meta-analysis aimed to evaluate the prevalence and risk factors of cognitive impairment in Chinese patients with hypertension.

**Methods:**

Two reviewers independently searched PubMed, Web of Science, Embase, The Cochrane Library, CNKI, CBM, the Wanfang database, and the VIP database from their inception to 7 June 2023. The gray literature and the reference lists of the included studies were also retrieved manually. Moreover, we also independently performed the eligibility screening, data extraction, and data synthesis. The primary outcome was the prevalence of cognitive impairment in Chinese patients with hypertension, and the secondary outcomes were the risk factors for cognitive impairment in patients with hypertension. R (version 4.0.3) was used for data synthesis.

**Results:**

In total, 82 studies involving 53,623 patients with hypertension were included in this meta-analysis. The pooled prevalence of cognitive impairment in patients with hypertension was 37.6% (95% CI: 33.2–42.2%). A total of 12 risk factors, including advanced age (r = −0.34, 95% CI: −0.45, −0.21), female sex (OR = 1.15, 95% CI: 1.01–1.32), BMI > 24 Kg/m^2^ (OR = 1.76, 95% CI: 1.04–3.00), lower educational level (OR = 2.01, 95% CI: 1.10–3.67), single status (OR = 1.63, 95% CI: 1.32–2.02), complications with diabetes (OR = 1.44, 95% CI: 1.14–1.80), coronary heart disease (OR = 1.49, 95% CI: 1.12–1.97), higher stage of hypertension [stage 3 vs. stage 1, OR = 3.08, 95% CI: 1.82–5.22; stage 2 vs. stage 1, OR = 1.83, 95% CI: 1.29–2.60], no regular physical activity (OR = 0.40, 95% CI: 0.21–0.77), higher levels of systolic blood pressure (r = −0.25, 95% CI: −0.42, −0.08), Hcy (r = −0.39, 95% CI: −0.63, −0.09), and IL-6 (r = −0.26, 95% CI: −0.48, −0.02) were detected.

**Conclusion:**

Cognitive impairment is prevalent in Chinese patients with hypertension, and the increased prevalence was associated with several demographic characteristics, complicated disease, no regular physical activity, worse hypertension status (higher stages and SBP), and high levels of biomarkers. Therefore, more attention should be paid to the early identification and treatment of patients with hypertension who are at high risk for cognitive impairment in clinical practice. In addition, relevant risk factors should be controlled to reduce the incidence of cognitive impairment.

**Systematic review registration:**

http://www.crd.york.ac.uk/PROSPERO, identifier [CRD42023410437].

## 1 Introduction

Hypertension is characterized by sustained blood pressure (BP) elevations and is associated with target organ damage and an increased risk of cardiovascular disease (CVD) (1, 2). According to a recent report (3), as of 2023 there were approximately 256.7 million adults with hypertension in China, accounting for nearly one-fifth of the 1.3 billion patients worldwide. However, only 16% of them had their BP under control. Additionally, the Chinese age-standardized prevalence of hypertension has increased from 24.7 to 27% in recent years (4), of which stage 2 and higher hypertension accounted for approximately 41% (5).

Cognitive impairment is a common complication of hypertension, usually consisting of a decline in memory, attention, visuospatial abilities, and executive functions (6). Cognitive impairment is the main cause of disability in the elderly, affecting nearly 50 million individuals worldwide, and is expected to increase to more than 130 million individuals by 2050 (7). According to the reports, apart from age, hypertension is the most important risk factor for cerebrovascular pathology, which increases the incidence of cognitive impairment and accelerates the transition to dementia (6, 8, 9). Several studies have concluded that the risk of cognitive dysfunction is increased to 40% in patients with hypertension, as opposed to those without hypertension (10–12). In addition, hypertension complicated with cognitive impairment was associated with a higher risk of depression (13), motor dysfunction (14), poor quality of life (14), and a higher incidence of falls and syncope (15), which contributed to a high mortality rate. According to the World Health Organization (WHO), hypertension complicated with cognitive impairment is a growing public health concern, especially in developing countries (16).

A previous meta-analysis (17) evaluated the global prevalence of mild cognitive impairment (MCI) in patients with hypertension. However, the prevalence of moderate and severe cognitive impairment was unclear. Furthermore, the risk factors had not been investigated yet. Considering the high prevalence and poor control rate in China, exploration of the prevalence and risk factors of cognitive impairment in Chinese patients with hypertension would be beneficial for developing effective management strategies. Therefore, the present study aimed to perform quantitative syntheses of the prevalence and investigate the risk factors of cognitive impairment in Chinese patients with hypertension. Moreover, association analysis was conducted to explore the relationship between potential risk factors and cognitive function. The results of this systematic review and meta-analysis would provide current evidence for clinicians and policymakers in the management of hypertension complicated by cognitive impairment.

## 2 Methods

This systematic review and meta-analysis was conducted according to the Preferred Reporting Items for Systematic Reviews and Meta-Analyses (PRISMA) statement (18), and the study protocol was registered with PROSPERO (CRD: 42023410437). The PRISMA checklist is shown in Supplementary Table 1.

### 2.1 Search strategy

Two reviewers (X Luo and YM Gong) independently searched PubMed, Web of Science, Embase, The Cochrane Library, CNKI, CBM, the Wanfang database, and the VIP database from their inception to 7 June 2023. Search strategies were developed using a combination of Medical Subject Headings (MeSH) and free text words related to hypertension and cognitive impairment. The gray literature and reference lists of the included studies were manually retrieved. In addition, experts in relevant fields were consulted for other possible studies. The detailed search strategies are shown in Supplementary Table 2.

### 2.2 Inclusion criteria

The inclusion criteria for this study were: (1) observational studies, including cross-sectional, cohort, and case–control studies, without the limitation of publication year; (2) Chinese population aged over 18 years without limitation of region and sex; (3) the prevalence of cognitive impairment in Chinese patients with hypertension was reported or could be calculated; (4) hypertension was diagnosed according to the WHO (19) or the 2010 Chinese guidelines for the treatment of hypertension (20), of which SBP ≥140 mmHg, and/or DBP ≥90 mmHg; (5) cognitive impairment was evaluated by recognized assessment tools with good reliability and validity; (6) the language of the published studies was limited to English or Chinese.

### 2.3 Exclusion criteria

The exclusion criteria for this study were: (1) the materials found being reviews, letters, conference reports, or protocols; (2) patients with hypertension complicated by stroke, Parkinson’s disease, traumatic brain injury, or other diseases that affected cognitive function; (3) the time and regions of the investigation were not reported; (4) overlapping publications; (5) the data were unavailable or could not be retrieved using different approaches.

### 2.4 Study selection

The retrieved records were imported into Endnote software (version X9) and the duplicate records were removed. Then, two independent reviewers (C Xie and DL Zhong) screened the titles and abstracts of the remaining records to exclude irrelevant literature. Subsequently, the full texts of the remaining studies were reviewed to assess eligibility. Any disagreements were arbitrated by a third reviewer (XB Liu).

### 2.5 Data extraction

Two reviewers (C Xie and DL Zhong) independently extracted the data using a pre-designed form. The following details were extracted: (1) the basic characteristics of the study: the first author, publication year, study site, and sample size; (2) the details of the participants, such as age, sex, body weight status, educational level, family status, etc.; (3) the assessment tools for cognitive impairment; (4) the outcomes: the primary outcome was the prevalence of cognitive impairment in patients with hypertension, while the secondary outcomes were the risk factors of cognitive impairment in patients with hypertension. The extracted data were cross-checked by the two reviewers. Any discrepancies were resolved through team discussion.

### 2.6 Risk of bias assessment

The risk of bias in the included studies was assessed by two independent reviewers (Y Zhang and LL Zhang) using the tool developed by Hoy et al. (21) to assess the risk of bias in epidemiologic studies. The tool comprises 10 items and a summary assessment: items 1–4 describe the external validity, including selection and nonresponse bias; items 5–10 assess the internal validity, including measurement and analysis bias. Each item is rated as “low risk” or “high risk” and the overall risk of bias is dependent on the number of “low risk” items. An overall low risk of bias is defined as 9–10 items with “low risk”; an overall moderate risk of bias is defined as 6–8 items with “low risk”; and an overall high risk of bias is defined as less than 5 items with “low risk.”

### 2.7 Statistical analysis

Data synthesis was performed using the meta packages of R (version 4.0.3). Before pooling the prevalence, we conducted normality tests based on logit-transformed proportions. The proportion of cognitive impairment in patients with hypertension was synthesized to calculate a pooled prevalence with a 95% confidence interval (CI). When analyzing the risk factors, the odds ratio (OR) was chosen as the effect size for the categorical variables (sex, body weight status, educational level, etc.), while the Pearson r correlation coefficient was chosen for continuous variables (age, level of SBP and DBP, etc.) (22). Heterogeneity was assessed by the Cochran Q test and the statistical value of *I^2^*, and it was considered significant heterogeneity if *I^2^* ≥ 50% and *p* < 0.1, while *I^2^* ≤ 50%, *p* > 0.1 meant nonsignificant heterogeneity. The random effects model was selected for the synthesis of results.

### 2.8 Subgroup analysis

Subgroup analysis was conducted according to the following aspects: (1) basic characteristics of the participants: sex (male, female subjects), body weight status (BMI ≤ 24 Kg/m^2^, >24 Kg/m^2^), educational level (primary school and below, middle school and above), family status (married, single); (2) recruitment source (community-based, hospitalized); (3) condition of the disease: duration of hypertension (<5 years, 5–10 years, >10 years), complications [diabetes, coronary heart disease (CHD)], classification of hypertension (stage 1, stage 2, stage 3); severity of cognitive impairment (mild, moderate, severe); (4) lifestyle: smoking and drinking, and engagement in regular physical activity; (5) assessment tools for cognitive impairment; (6) study design (cross-sectional studies, cohort studies).

### 2.9 Meta-regression

Univariable meta-regression analysis was conducted to explore the source of heterogeneity from the following characteristics: study period (before 2010, between 2010 and 2015, between 2015 and 2020, after 2020); regions (Nationwide, Central China, East China, North China, Northeast China, Northwest China, South China, Southwest China), recruitment source (community-based, hospitalized), study design (cross-sectional studies, cohort studies), assessment tools for cognitive impairment, sample size (0–100, 100–500, 500–1,000, >1,000), and risk of bias (low, moderate, high).

### 2.10 Sensitivity analysis

A sensitivity analysis was conducted to verify the stability of the main findings by eliminating the included studies one by one.

### 2.11 Publication bias

A funnel plot and *Egger’s* test were performed to assess the publication bias. The trim and fill method was used to verify the stability in cases of existing publication bias.

### 2.12 Certainty of evidence

The certainty of the evidence was assessed according to the GRADE guidelines using the GRADEpro GDT.^1^ For the primary outcome, we assessed the following five domains: limitations, inconsistency, indirectness, imprecision, and publication bias. The certainty of the evidence was reported in four categories, including high, moderate, low, and very low. Observational studies were classified as low-certainty evidence.

## 3 Results

### 3.1 Study selection

**Figure 1:**
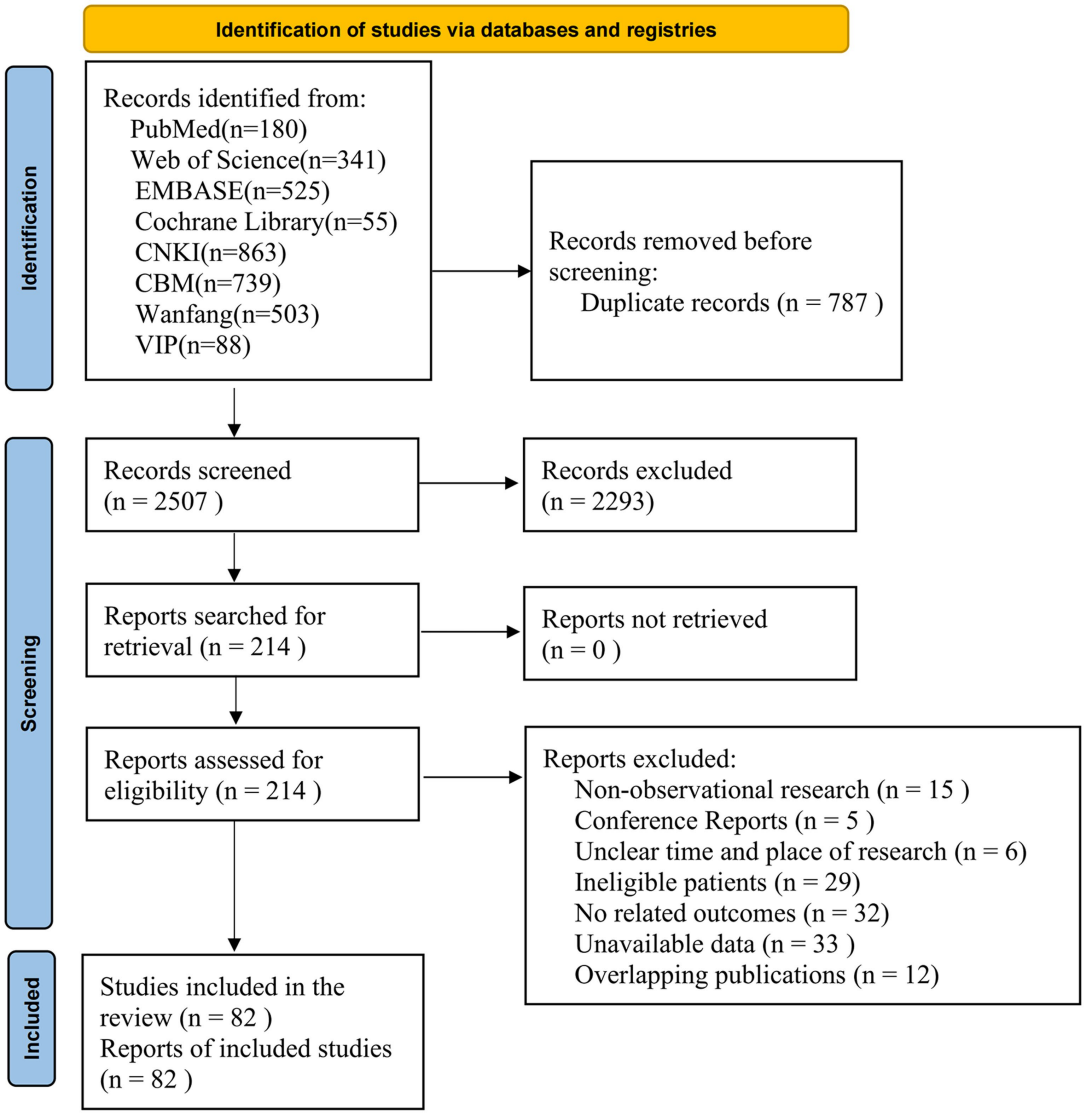
The flow chart of study selection.

A total of 3,294 records were identified from electronic databases. After removing 787 duplicates, 2,293 irrelevant records were excluded by screening the titles and abstracts. The full texts of the remaining 197 records were reviewed and 82 studies were finally included. The list of excluded studies with reasons is presented in Supplementary Table 3. The flow chart is shown in Figure 1.

### 3.2 Study characteristics

The basic characteristics of the included studies are summarized in Supplementary Table 4. A total of 53,623 patients with hypertension were included in this meta-analysis. Regarding the study regions, 3 studies were from the nationwide survey, 8 studies were from Central China, 21 studies were from East China, 21 studies were from North China, 1 study was from South China, 7 studies were from Northeast China, 13 studies were from Northwest China, and 8 studies were from Southwest China. The sample size ranged from 55 to 11,270 patients, and the mean age of the patients ranged from 38 to 93 years. The Mini-mental State Examination (MMSE), Montreal Cognitive Assessment (MoCA), Peterson, Clinical Dementia Rating, Clock Drawing Test, AD-8, Basic Cognitive Aptitude Tests, and clinical memory scale were used to assess cognitive function.

### 3.3 Risk of bias

The risk of bias in the included studies is described in Supplementary Table 5. In terms of the representation of the target population, four studies were judged as low risk, while the remaining studies were assessed as high risk because the target population could not represent the national population. The sampling frame of 15 studies was close to the target population, so they were rated as low risk, and the remaining studies were rated as high risk. Regarding the random selection of the sample, 15 studies selected the samples randomly and were assessed as low risk, while the remaining 67 studies failed to implement randomization and were considered high risk. Regarding the data collected directly from subjects, Luo et al. (23) collected these indirectly and assessed them as high risk, while the remaining 81 studies directly synthesized the data and were rated as low risk. With regard to the domains of proceeded minimal non-response bias, appropriate case definition, validated measure, consistent mode of data collection, appropriate length of the shortest prevalence period, and correct calculation of prevalence, all included studies were assessed as low risk. In summary, the overall risk of bias in 15 studies was rated as low, while 67 studies were rated as having a moderate risk of bias.

### 3.4 Prevalence of cognitive impairment in Chinese patients with hypertension

**Figure 2:**
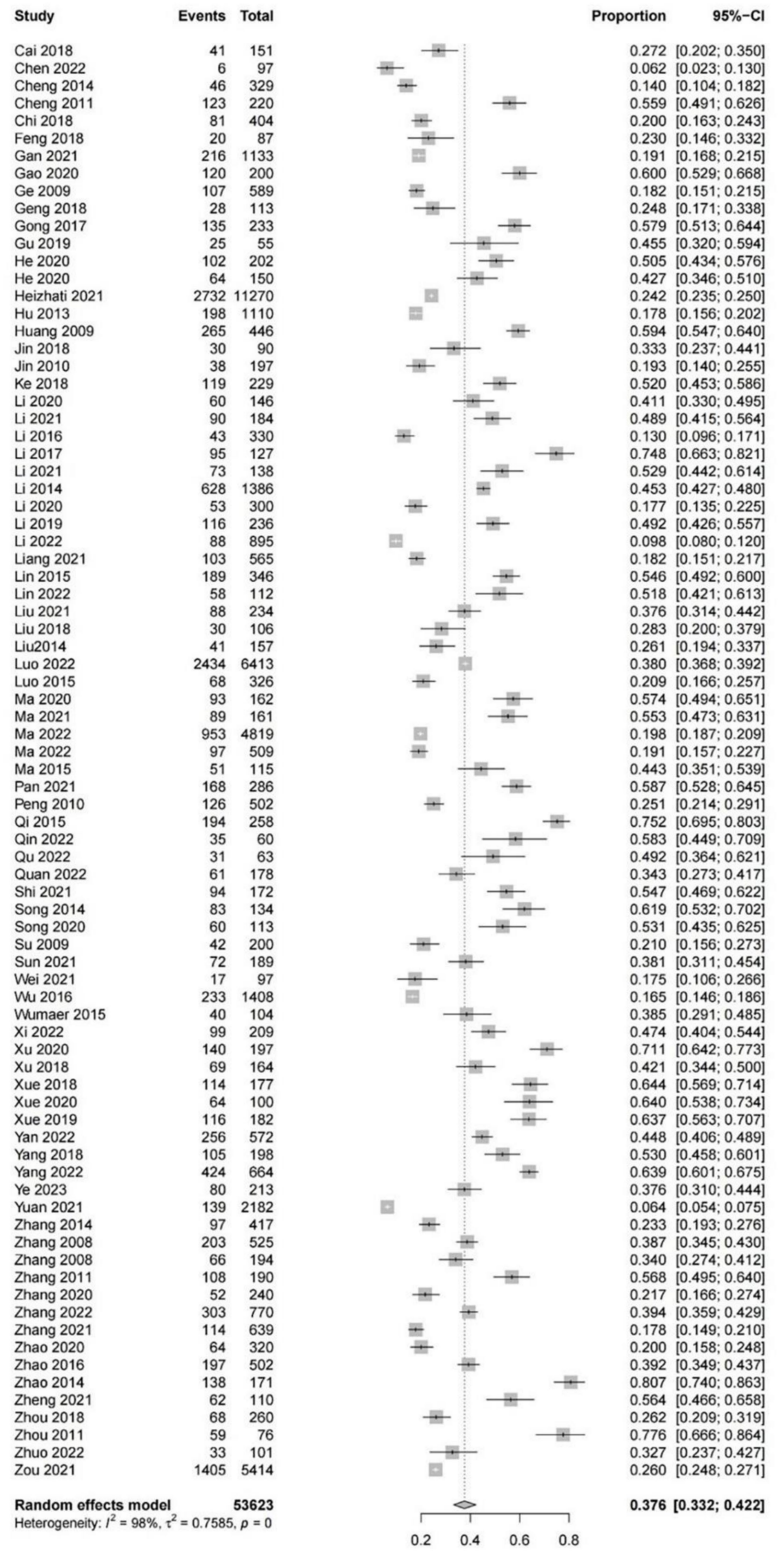
The forest plot of the overall pooled prevalence of cognitive impairment in Chinese patients with hypertension.

Based on 82 studies involving 53,623 patients with hypertension, the pooled overall prevalence of cognitive impairment in the subjects was 37.6% (95% CI: 33.2–42.2%, *I^2^* = 98%, *p* < 0.01; Figure 2). Regarding the severity of cognitive impairment, the prevalence of MCI in Chinese patients with hypertension was 37.0% (95% CI: 29.6–45.1%, *I^2^* = 99%, *p* < 0.01), which was higher than the prevalence of moderate (23.7, 95% CI: 14.5–36.3%, *I^2^* = 90%, *p* < 0.01) and severe cognitive impairment (11.7, 95% CI: 3.7–31.6%, *I^2^* = 93%, *p* < 0.01; Supplementary Figure 1). In terms of the assessment tools for cognitive impairment, the prevalence of cognitive impairment in Chinese patients with hypertension was higher on the basis of MoCA (48.7, 95% CI: 40.0–57.5%, *I^2^* = 97%, *p* < 0.01) than that based on MMSE + MoCA (48.1, 95% CI: 41.4–54.9%, *I^2^* = 91%, *p* < 0.01) and MMSE (32.0, 95% CI: 26.9–37.6%, *I^2^* = 98%, *p* < 0.01; Supplementary Figure 2). As for recruitment source, the prevalence of cognitive impairment in hospitalized patients with hypertension (40.8, 95%CI: 36.0–45.9%, *I^2^* = 96%, *p* < 0.01) was higher than that in community-based patients (26.8, 95% CI: 19.1–36.1%, *I^2^* = 99%, *p* < 0.01; Supplementary Figure 3). With respect to the study design, the prevalence of cognitive impairment in patients with hypertension from cross-sectional studies (40.8, 95% CI: 36.1–45.7%, *I^2^* = 98%, *p* < 0.01) was higher than that in cohort studies (25.9, 95% CI, 18.3–35.2%, *I^2^* = 99%, *p* < 0.01; Supplementary Figure 4).

### 3.5 Risk factors for cognitive impairment in Chinese patients with hypertension

The detailed results of the risk factors are summarized in Table 1.

**Table 1:** Subgroup analysis and risk factors for cognitive impairment in hypertension.

Risk factors	Number of studies (n)	Number of subjects (n)	Pooled prevalence (95%CI)	Heterogeneity		OR/r (95%CI)	Heterogeneity	
				*I^2^*	*P*		*I^2^*	*P*
**Gender**								
Male subjects	49	13,695	32.1% (31.6–43.0%)	97%	<0.01	1.0	/	/
Female subjects	48	15,974	40.3% (34.9–45.9%)	97%	<0.01	1.15 (1.01–1.32)	91%	<0.01
**Body weight status**								
BMI ≤ 24	5	4,532	33.4% (24.3–43.8%)	78%	<0.01	1.0	/	/
BMI > 24	5	2,859	46.0% (36.5–55.7%)	96%	<0.01	1.76 (1.04–3.00)	91%	<0.01
**Educational level**								
Middle school and above	16	5,829	26.9% (17.6–38.8%)	96%	<0.01	1.0	/	/
Primary school and below	15	4,258	36.5% (26.1–48.2%)	97%	<0.01	2.01 (1.10–3.67)	98%	<0.01
**Family status**								
Married	9	10,643	21.4% (13.4–32.2%)	99%	<0.01	1.0	/	/
Single	9	2,822	31.7% (21.2–44.6%)	97%	<0.01	1.63 (1.32–2.02)	61%	<0.01
**Duration of hypertension**								
<5 years	2	2,363	24.2% (16.5–34.0%)	91%	<0.01	1.0	/	/
5~10 years	2	472	30.8% (12.1–58.9%)	97%	<0.01	1.29 (0.49–3.41)	88%	<0.01
>10 years	2	2,295	33.5% (17.1–55.1%)	97%	<0.01	1.08 (0.93–1.25)	0%	0.33
**Complication with diabetes**								
Without diabetes	18	7,915	39.9% (31.7–48.7%)	95%	<0.01	1.0	/	/
With diabetes	18	1724	47.8% (38.3–57.4%)	90%	<0.01	1.44 (1.14–1.80)	64%	<0.01
**Complication with CHD**								
Without CHD	14	7,459	35.5% (24.9–47.6%)	94%	<0.01	1.0	/	/
With CHD	14	1,197	44.6% (32.9–56.9%)	96%	<0.01	1.49 (1.12, 1.97)	59%	<0.01
**Classification of hypertension**								
I	10	1,105	28.5% (20.6–37.9%)	88%	<0.01	1.0	/	/
II	12	1,467	40.1% (29.1–52.1%)	90%	<0.01	1.83 (1.29–2.60)	61%	<0.01
III	12	913	55.5% (37.6–72%)	87%	<0.01	3.08 (1.82–5.22)	73%	<0.01
**Smoking habits**								
Smokers	24	4,643	38.7% (31.8–46.2%)	96%	<0.01	1.0	/	/
Non-smokers	24	2,932	37.3% (30.2–44.9%)	90%	<0.01	0.93 (0.76–1.15)	60%	<0.01
**Drinking habits**								
No drinking habits	20	8,734	32.8% (26.0%-40.4)	96%	<0.01	1.0	/	/
Drinking habits	20	1859	33.5% (25.3–42.9%)	93%	<0.01	1.07 (0.85–1.35)	52%	<0.01
**Physical activity**								
Regular physical activity	7	1777	45.1% (25.4–66.4%)	98%	<0.01	1.0	/	/
No regular physical activity	7	6,241	24.4% (9.5–49.9%)	98%	<0.01	0.40 (0.21–0.77)	92%	<0.01
Age	4	903	/	/	/	−0.34 (−0.45, −0.21)	75%	<0.001
SBP	3	732	/	/	/	−0.25 (−0.42, −0.08)	82%	0.005
DBP	3	742	/	/	/	0.08 (−0.18–0.33)	91%	0.54
Hcy	4	691	/	/	/	−0.39 (−0.63, −0.09)	94%	0.01
hs-CRP	2	412	/	/	/	−0.04 (−0.13–0.06)	0%	0.47
IL-6	3	672	/	/	/	−0.26 (−0.48, −0.02)	87%	0.03

#### 3.5.1 Sex

A total of 49 studies included 13,695 male and 15,974 female patients with hypertension. The results indicated that female patients with hypertension had a higher prevalence of cognitive impairment (40.3, 95% CI: 34.9–45.9%, *I^2^* = 97%, *p* < 0.01) than the male patients (37.1, 95% CI: 31.6–43.0%, *I^2^* = 97%, *p* < 0.01; Supplementary Figure 5A). The OR for female subjects vs. male subjects was 1.15 (95% CI: 1.01–1.32, *I^2^* = 91%, *p* < 0.01; Supplementary Figure 5B).

#### 3.5.2 Body weight status

Five studies reported the prevalence of cognitive impairment in patients with hypertension based on BMI >24 Kg/m^2^ (2,859 participants) and BMI ≤ 24 Kg/m^2^ (4,532 participants). The patients with hypertension whose BMI > 24 Kg/m^2^ had a higher prevalence of cognitive impairment (46.0, 95% CI: 36.5–55.7%, *I^2^* = 96%, *p* < 0.01) than those whose BMI ≤24 Kg/m^2^ (33.4, 95% CI: 24.3–43.8%, *I^2^* = 78%, *p* < 0.01; Supplementary Figure 6A). The OR between patients with BMI >24 Kg/m^2^ and those with BMI ≤24 Kg//m^2^ was 1.76 (95% CI: 1.04–3.00, *I^2^* = 91%, *p* < 0.01; Supplementary Figure 6B).

#### 3.5.3 Educational level

According to the educational level, 18 studies with 4,258 patients in primary school and below, and 5,829 patients in middle school and above with hypertension were included. The prevalence of cognitive impairment was higher in patients with hypertension in primary school and below (36.5, 95% CI: 26.1–48.2%, *I^2^* = 97%, *p* < 0.01) than in patients in middle school and above (26.9, 95% CI: 17.6–38.8%, *I^2^* = 96%, *p* < 0.01; Supplementary Figure 7A). The OR of patients with hypertension in primary school and below compared with those in middle school and above was 2.01 (95% CI: 1.10–3.67, *I^2^* = 85%, *p* < 0.01; Supplementary Figure 7B).

#### 3.5.4 Family status

In total, nine studies reported that 10,643 patients with hypertension were married and 2,822 patients were single. Single patients with hypertension showed a higher prevalence of cognitive impairment (31.7, 95% CI: 21.2–44.6%, *I^2^* = 97%, *p* < 0.01) than married patients (21.4, 95% CI: 13.4–32.2%, *I^2^* = 99%, *p* < 0.01; Supplementary Figure 8A). The OR for the single patients compared with the married patients was 1.63 (95% CI: 1.32–2.02, *I^2^* = 61%, *p* < 0.01; Supplementary Figure 8B).

#### 3.5.5 Duration of hypertension

The duration of hypertension was dichotomized into <5 years, 5–10 years, and > 10 years. Among patients with hypertension, the prevalence of cognitive impairment in patients with a duration of hypertension >10 years was 33.5% (95% CI: 17.1–55.1%, *I^2^* = 97%, *p* < 0.01), followed by those with a duration of 5–10 years (30.8, 95% CI: 12.1–58.9%, *I^2^* = 97%, *p* < 0.01) and < 5 years (24.2, 95% CI: 16.5–34.0%, *I^2^* = 91%, *p* < 0.01; Supplementary Figure 9A). However, no statistical difference was detected among the three groups (5–10 vs. < 5, OR = 1.29, 95% CI: 0.49–3.41, *I^2^* = 88%, *p* < 0.01; >10 vs. < 5, OR = 1.08, 95% CI: 0.93–1.25, *I^2^* = 0%, *p* = 0.33; Supplementary Figures 9B,C).

#### 3.5.6 Diabetes/CHD complications

There were 1,724 patients with hypertension complicated by diabetes and 7,915 patients without diabetes in the 18 included studies. The findings revealed that patients with hypertension complicated with diabetes had a higher prevalence of cognitive impairment (47.8, 95% CI: 38.3–57.4%, *I^2^* = 90%, *p* < 0.01) than those without diabetes (39.9, 95% CI: 31.7–48.7%, *I^2^* = 95%, *p* < 0.01; Supplementary Figure 10A). The OR in those with diabetes compared with those without diabetes was 1.44 (95% CI: 1.14–1.80, *I^2^* = 64%, *p* < 0.01; Supplementary Figure 10B).

A total of 14 studies included 1,197 and 7,459 patients with hypertension complicated with and without CHD, respectively. A higher prevalence of cognitive impairment was observed in patients with CHD (44.6, 95% CI: 32.9–56.9%, *I^2^* = 94%, *p* < 0.01) compared to those without CHD (35.5, 95% CI: 24.9–47.6%, *I^2^* = 96%, *p* < 0.01; Supplementary Figure 10C). The OR in patients with CHD compared to those without CHD was 1.49 (95% CI: 1.12–1.97, *I^2^* = 59%, *p* < 0.01; Supplementary Figure 10D).

#### 3.5.7 Classification of hypertension

In total, 12 studies included 1,105, 1,467, and 913 patients with stage 1, stage 2, and stage 3 hypertension, respectively. The prevalence of cognitive impairment in patients with stage 3 hypertension (55.5, 95% CI: 37.6–72.0%, *I^2^* = 87%, *p* < 0.01) was higher than that in patients with stage 2 hypertension (40.1, 95% CI: 29.1–52.1%, *I^2^* = 90%, *p* < 0.01) and stage 1 hypertension (28.5, 95% CI: 20.6–37.9%, *I^2^* = 88%, *p* < 0.01; Supplementary Figure 11A). There was a statistical difference among the three groups (stage 3 vs. stage 1, OR = 3.08, 95% CI: 1.82–5.22, *I^2^* = 73%, *p* < 0.01; stage 2 vs. stage 1, OR = 1.83, 95% CI: 1.29–2.60, *I^2^* = 61%, *p* < 0.01; Supplementary Figures 11B,C).

#### 3.5.8 Smoking habits

A total of 24 studies evaluated the prevalence of cognitive impairment in 2,932 and 4,643 patients with hypertension with and without smoking habits, respectively. The prevalence of cognitive impairment was higher in patients with hypertension without smoking habits (38.7, 95% CI: 31.8–46.2%, *I^2^* = 96%, *p* < 0.01) than in those with smoking habits (37.3, 95% CI: 30.2–44.9%, *I^2^* = 90%, *p* < 0.01; Supplementary Figure 12A). The OR of patients with hypertension with smoking habits compared to those without smoking habits was 0.93 (95% CI: 0.76–1.15, *I^2^* = 60%, *p* < 0.01; Supplementary Figure 12B).

#### 3.5.9 Drinking habits

There were 20 studies with 1,859 and 8,734 patients with hypertension with and without drinking habits, respectively. The prevalence of cognitive impairment was 33.5% (95% CI: 25.3–42.9%, *I^2^* = 93%, *p* < 0.01) in patients with hypertension with drinking habits and 32.8% (95% CI: 26.0–40.4%, *I^2^* = 96%, *p* < 0.01) in those without drinking habits (Supplementary Figure 13A). No statistical difference was detected between the two groups (with drinking habits vs. without drinking habits, OR = 1.07, 95% CI: 0.85–1.35, *I^2^* = 52%, *p* < 0.01; Supplementary Figure 13B).

#### 3.5.10 Regular physical activity

Seven studies investigated the prevalence of cognitive impairment in 6,241 and 1,777 patients with hypertension who engaged in regular physical activity and those who did not. A higher prevalence was observed in patients without regular physical activity (45.1, 95% CI: 25.4–66.4%, *I^2^* = 98%, *p* < 0.01; Supplementary Figure 14A) than in those with regular physical activity (24.4, 95% CI: 9.5–49.9%, *I^2^* = 98%, *p* < 0.01). Compared with those without regular physical activity, the OR for those with regular physical activity was 0.40 (95% CI: 0.21–0.77, *I^2^* = 92%, *p* < 0.01; Supplementary Figure 14B).

#### 3.5.11 Age

The pooled results of four studies indicated that age was negatively associated with cognitive function in patients with hypertension (r = −0.34, 95% CI: −0.45, −0.21, *I^2^* = 75%, *p* < 0.001; Supplementary Figure 15).

#### 3.5.12 SBP

The pooled results demonstrated that there was a negative correlation between SBP and cognitive function in patients with hypertension (r = −0.25, 95% CI: −0.42, −0.08, *I^2^* = 82%, *p* = 0.005; Supplementary Figure 16).

#### 3.5.13 DBP

No significant association was detected between DBP and cognitive function in patients with hypertension (r = 0.08, 95% CI: −0.18–0.33, *I^2^* = 91%, *p* = 0.54; Supplementary Figure 17).

#### 3.5.14 Hcy

Hcy was adversely associated with cognitive function in patients with hypertension (r = −0.39, 95% CI: −0.63, −0.09, *I^2^* = 94%, *p* = 0.01; Supplementary Figure 18).

#### 3.5.15 hs-CRP

There was no correlation between hs-CRP and cognitive function in patients with hypertension (r = −0.04, 95% CI: −0.13–0.06, *I^2^* = 0%, *p* = 0.47; Supplementary Figure 19).

#### 3.5.16 Interleukin-6

IL-6 was inversely correlated with cognitive function in patients with hypertension (r = −0.26, 95% CI: −0.48, −0.02, *I^2^* = 87%, *p* = 0.03; Supplementary Figure 20).

### 3.6 Sensitivity analysis

The pooled prevalence of cognitive impairment in patients with hypertension varied from 37.1 to 38.2% after omitting the studies one by one, which indicated good stability (Supplementary Figure 21).

### 3.7 Publication bias

**Figure 3:**
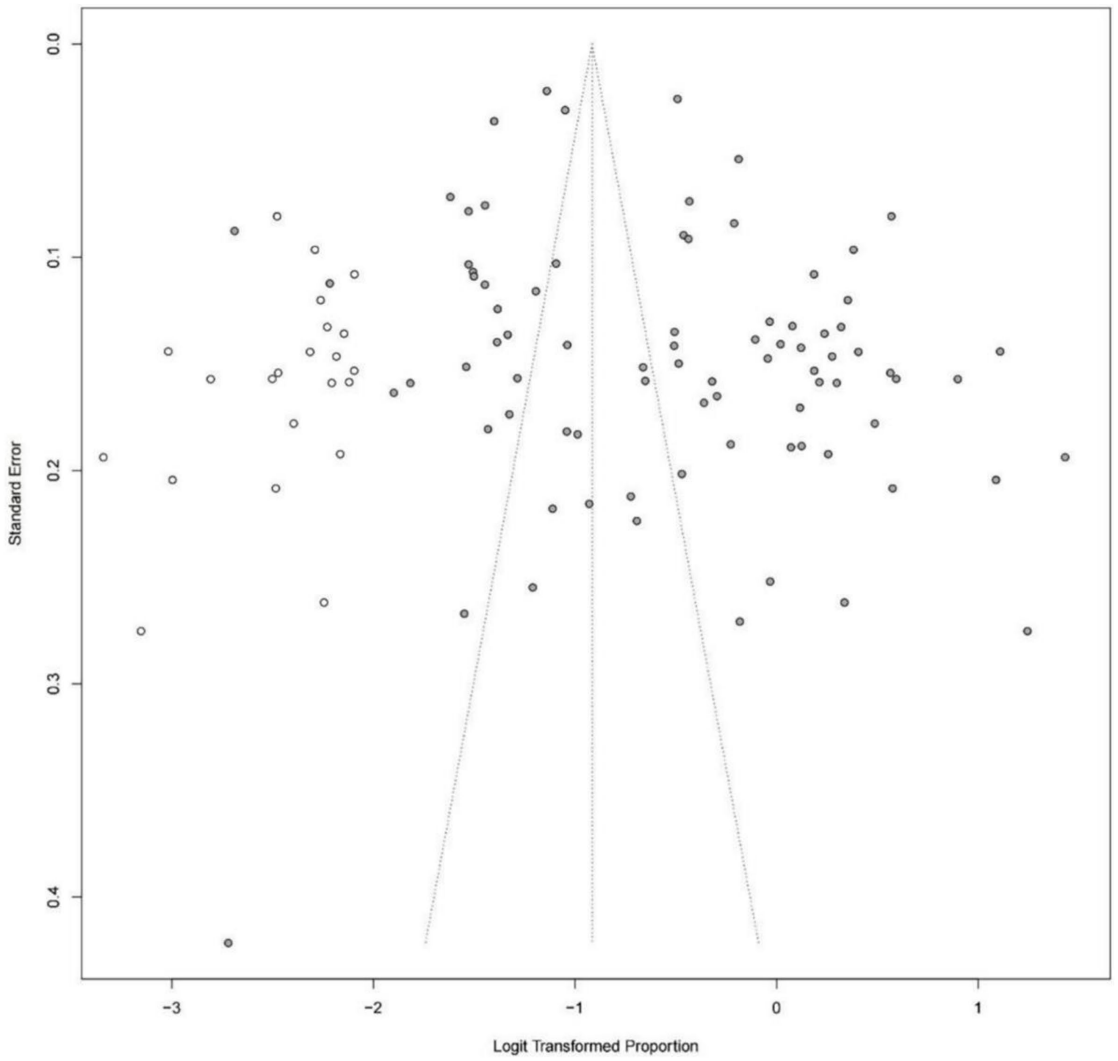
Funnel plot after applying trim and fill method.

The asymmetry funnel plot and the results of *Egger’s* test (*p* = 0.0029) indicated that publication bias existed in the prevalence of cognitive impairment in patients with hypertension. The trim and fill method was used and the results remained consistent with 22 additional virtual studies (Figure 3).

### 3.8 Meta-regression analysis

The results of univariate meta-regression showed that the study characteristics of recruitment source, study design, and cognitive impairment assessment tools could be potential sources of heterogeneity (Supplementary Table 6).

### 3.9 Certainty of evidence

The certainty of evidence was assessed for the prevalence of cognitive impairment in patients with hypertension. The certainty for the prevalence of cognitive impairment in Chinese patients with hypertension was upgraded from very low to low because of “a large sample size effect” (Supplementary Table 7).

## 4 Discussion

### 4.1 Cognitive impairment is prevalent in Chinese patients with hypertension

The present study included 82 studies involving 53,623 hypertensive patients, and the pooled prevalence of cognitive impairment (mild to severe) in Chinese patients with hypertension was 37.6%, indicating that cognitive impairment was prevalent. Inconsistently, according to the subgroup analysis of a recent meta-analysis (17), it was reported that the prevalence of MCI in Asian hypertensive patients was 26%, which was lower than the global prevalence of 30%. Such differences may be attributed to different study regions (China vs. Asia). Several epidemiological investigations have been conducted in Argentina (24), Spain (25) and Poland (26), reporting a prevalence of 22.1%, 15.7%, and 17.7%, respectively. As mentioned above, the prevalence of cognitive impairment in China was higher than in other countries. Thus, clinicians and policymakers in China should pay more attention to the control of cognitive impairment in patients with hypertension.

The prevalence varied strikingly among the included studies. Four potential influencing factors were detected: (1) the severity of cognitive impairment: MCI was most prevalent in Chinese patients with hypertension (37.0%) compared with moderate (23.7%) and severe (11.7%) cognitive impairment. (2) the assessment tools for cognitive impairment: the prevalence based on MMSE was 32.0 and 48.7% when MoCA was used, whereas MoCA had a higher sensitivity for MCI (27). (3) recruitment source: our findings revealed that the prevalence of cognitive impairment was higher in hospitalized patients with hypertension than in community-based patients. Hospitalized patients are usually complicated by multiple chronic diseases which could affect their BP and cognitive function. (4) study design: cross-sectional studies (66 studies) reported a higher prevalence than cohort studies (16 studies). Because the association between prevalence and time was neglected for cross-sectional studies, a misestimation of the prevalence was inevitable (28).

### 4.2 Risk factors for cognitive impairment in Chinese patients with hypertension

In total, 12 risk factors (advanced age, female sex, BMI > 24 Kg/m^2^, lower educational level, single status, complications with diabetes and CHD, higher stages of hypertension, no regular physical activity, higher levels of SBP, Hcy, and IL-6) were associated with an increased risk for the development of cognitive impairment in patients with hypertension.

#### 4.2.1 Risk factors related to the demographic characteristics

The results showed that advanced age was associated with an increased risk of cognitive impairment in Chinese patients with hypertension. A previous review reported that the prevalence of cognitive impairment increased exponentially after the age of 65, nearly doubling every 5 years until the age of 90 (7). Aging accelerated the development of cerebral small vessel lipohyalinosis and atherosclerosis in the circle of Willis, possibly causing chronic cerebral hypoperfusion, which has been recognized as a crucial factor in cognitive impairment (29). Of interest, both advanced age and hypertension share similar mechanisms for cognitive impairment involving oxidative stress and endothelial dysfunction (30, 31). Notably, the majority of the included studies focused on elderly patients, so the age distribution might be a confounder in our study.

As indicated in this study, female patients with hypertension were more likely to suffer from cognitive impairment compared with male patients (OR = 1.15). A meta-analysis pointed out that women had a higher risk of progression to AD (RR = 1.33) (32). Similarly, another review reported a significantly higher prevalence of non-amnestic MCI in women (33). Holland et al. revealed that female patients with MCI or AD had a greater rate of brain atrophy and clinical decline in comparison to men over a 1-year period, reflecting a faster cognitive decline (34). Moreover, the decline in estrogen levels after menopause would contribute to higher levels of associated pathological substance deposition, resulting in poorer cognitive performance in women (35).

The results of subgroup analysis based on five studies showed that BMI > 24 Kg/m^2^ was associated with an increased risk of cognitive impairment in patients with hypertension. A previous meta-analysis showed that both overweight (RR = 1.26) and obesity (RR = 1.64) in midlife were associated with an increased risk of cognitive impairment (36). Another study indicated that obesity may influence hippocampal long-term potentiation and impair recognition memory, leading to cognitive dysfunction. However, another national longitudinal study based on the elderly population showed that obesity was protective against the development of dementia (HR = 0.44) and dementia-related mortality. The correlation between overweight or obesity and cognitive impairment in patients with hypertension needs further investigation.

Consistent with our findings, multiple studies have documented a negative correlation between educational level and the prevalence of cognitive impairment in patients with hypertension (37–40). The results are in line with the brain reserve hypothesis (41), which suggests that individuals with higher levels of education possess a greater cognitive reserve that allows them to better withstand and compensate for the decline in brain function. Meanwhile, higher educational levels may serve as a positive stimulus for the change of brain structure, biochemical metabolism, and complexity of polysynaptic connections, ultimately resulting in a lower risk of cognitive impairment (42).

Our results indicated that patients with hypertension who are single had a higher risk of cognitive impairment compared with married patients. A multicenter cohort study found that single status was associated with a higher risk of hypertension (43). Moreover, Skirbekk et al. (44) revealed that the RR values of cognitive impairment for the unmarried, continuously divorced, and intermittently divorced were 1.73, 1.66, and 1.50, respectively, when taking the continuously married as the reference. In addition, the evidence from France (45), Finland (46), and the United States (47) confirmed the correlation between marital status and cognitive impairment.

#### 4.2.2 Risk factors related to the features of hypertension

In line with our findings (15, 48), higher levels of hypertension were associated with an increased risk of cognitive impairment. In particular, SBP showed a considerable correlation with cognitive function. Launer et al. (49) discovered that each 10 mmHg increase in SBP was associated with an increased risk of cognitive decline. Another longitudinal study published in JAMA found that an elevated baseline SBP of ≥160 mmHg was associated with a 14% increased risk of cognitive dysfunction during 9 years of follow-up (50). Atherosclerosis, white matter lesions, increased neuritic plaques and tangles, and brain atrophy may be the possible mechanisms (29, 51).

Diabetes and CHD have been identified as risk factors for cognitive impairment in patients with hypertension. Dhikav et al. reported that cardiovascular risk factors, including diabetes, would increase the risk of cognitive impairment (52–54). In a meta-analysis of six prospective studies, diabetes carried a 47% increased risk of dementia (55). On the one hand, diabetes-associated abnormal cerebral angiogenesis and higher capillary density in the central nervous system may speed up the deterioration and leakage of blood vessels during neurodegenerative processes (56). On the other hand, insulin deficiency, the underlying pathology of diabetes, has been linked to higher levels of amyloid beta and increased tau phosphorylation, both of which have been connected to the aberrant metabolism of AD (57, 58). Similar to our findings, Ryuno et al. (59) reported that patients with hypertension and diabetes were more likely to experience cognitive decline than those with hypertension alone. Regarding CHD, a cross-sectional study in Inner Mongolia (60) showed that patients with CHD had a higher risk of developing MCI (OR = 3.9) and dementia (OR = 6.8). Greater degrees of coronary stenosis may lead to greater gray matter loss in specific brain regions that are relevant to cognitive function (61).

#### 4.2.3 Lifestyle-related risk factors

Engagement in regular physical activity would reduce the risk of cognitive impairment in patients with hypertension. Hamer et al. (62) performed a meta-analysis of 14 prospective studies and revealed that a lack of regular physical activity increased the risk of developing dementia by 39%. Researches have shown that physical activity can increase brain volume (63) and brain-derived neurotrophic factor levels (64). Erickson et al. (65) revealed that aerobic exercise was able to reverse hippocampal volume loss in late adulthood and enhance memory performance. Moreover, multiple meta-analyses have shown that various forms of physical activity can lower BP levels (66–68).

#### 4.2.4 Biomarker-related risk factors

In the present study, we observed an inverse association between Hcy and cognitive function in patients with hypertension. It has been reported that Hcy is involved in the formation and development of hypertension (69). In addition, high levels of Hcy could promote neuronal damage by inducing vascular damage, thus impairing cognitive function (70). Furthermore, higher levels of IL-6 have been associated with poorer cognitive performance (71) and faster cognitive decline (72). Levels of Hcy and IL-6 may serve as biomarkers to reflect the cognitive function of patients with hypertension.

### 4.3 Comparisons with previous SR

A recent SR conducted by Qin et al. (17) investigated the prevalence of MCI in hypertensive patients. Qin et al. reported that the global prevalence of MCI in patients with hypertension was 30%. Unlike the study conducted by Qin et al., we focused on cognitive impairment (mild to severe) in the Chinese population with hypertension. Because cognitive impairment was prevalent in Chinese hypertensive patients, exploration of the prevalence and risk factors of cognitive impairment in Chinese patients with hypertension would be beneficial for developing effective management strategies. Moreover, we conducted an extensive search and pooled data from 82 studies involving 53,623 patients with hypertension. In addition to the overall prevalence, we further identified 12 risk factors associated with cognitive impairment in Chinese patients with hypertension through detailed subgroup analyses and association analyses. Meanwhile, we assessed the certainty of the evidence using GRADE.

### 4.4 Limitations

This study had several limitations. First, there was significant heterogeneity among the included studies, and the potential sources of heterogeneity were recruitment source, study design, and cognitive impairment assessment tools according to the meta-regressions. Second, we calculated the value of OR based on the original data without adjusting for the potential discrepancy among the subjects, which resulted in unavoidable confounding effects. Third, because the characteristics of the study population varied, an ecological fallacy may exist.

## 5 Conclusion

Cognitive impairment was prevalent in Chinese patients with hypertension with a prevalence of 37.6%. The increased prevalence was associated with several demographic characteristics, complicated diseases, no regular physical activity, worse hypertension status, and high biomarker levels. More attention should be paid to the early identification and treatment of patients with hypertension who are at high risk for cognitive impairment in clinical practice.

## Data availability statement

The original contributions presented in the study are included in the article/Supplementary material, further inquiries can be directed to the corresponding author.

## Author contributions

CX: Writing – original draft. DZ: Writing – review & editing. YZ: Writing – review & editing. XLi: Conceptualization, Writing – review & editing. LZ: Conceptualization, Writing – review & editing. XLu: Validation, Writing – review & editing. YG: Data curation, Writing – review & editing. WJ: Investigation, Methodology, Visualization, Writing – review & editing. RJ: Methodology, Writing – review & editing. JL: Methodology, Writing – review & editing.

## Footnotes

^1^ http://gradepro.org/
