# Supplementary material for: Prevalence and risk factors of cognitive impairment in Chinese patients with hypertension: a systematic review and meta-analysis
Source: Front Neurol. 2024 Feb 13;14:1271437. doi: 10.3389/fneur.2023.1271437 (PMC10898355; doi:10.3389/fneur.2023.1271437)
Supplement: Supplementary file 1 [file Table_1.DOCX]

Supplementary Material

# Supplementary Figures and Tables

## Supplementary Figures


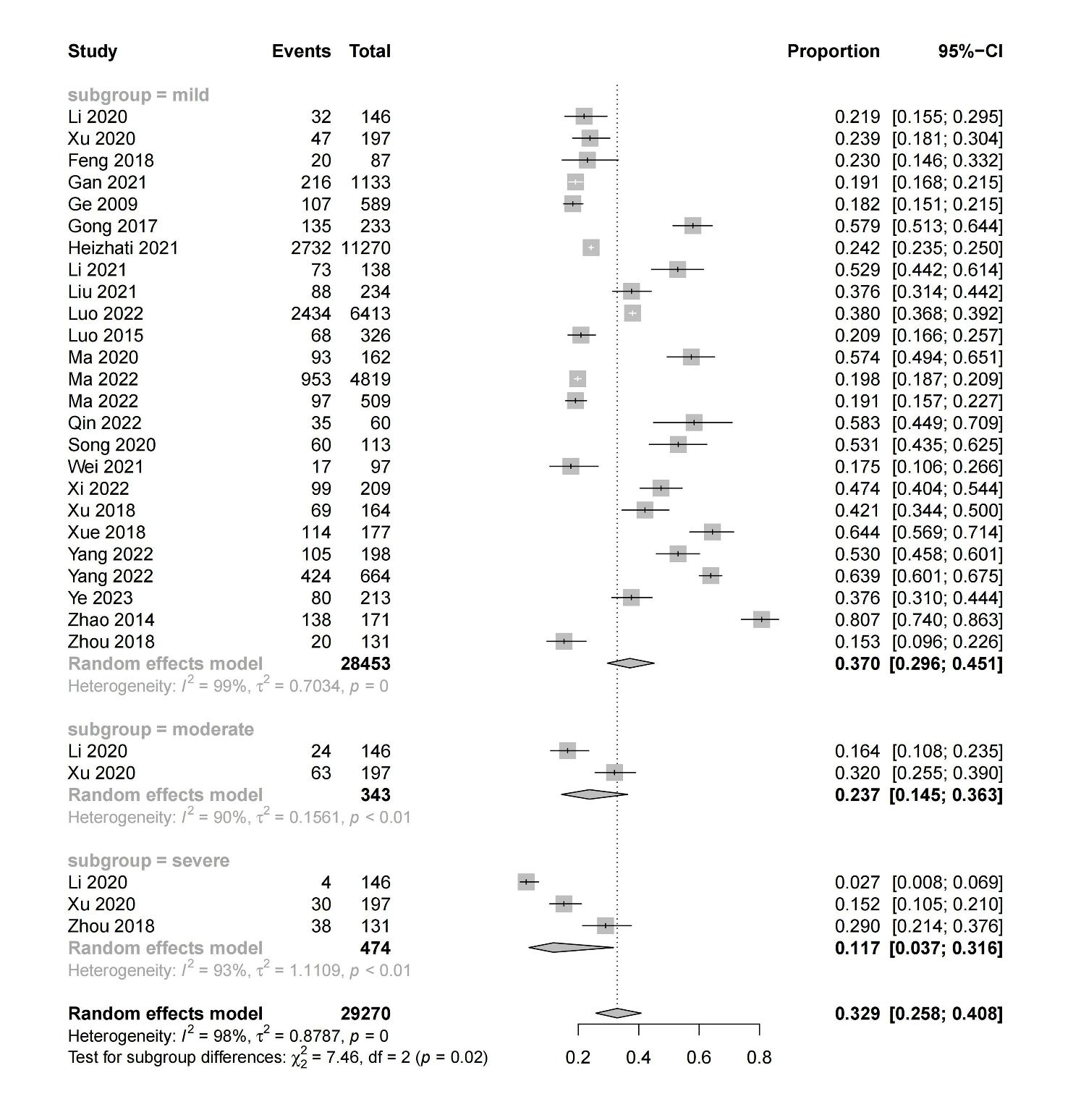


**Supplementary Figure 1.** **The forest plot of prevalence of cognitive impairment in Chinese hypertensive patients based on the severity of cognitive impairment**


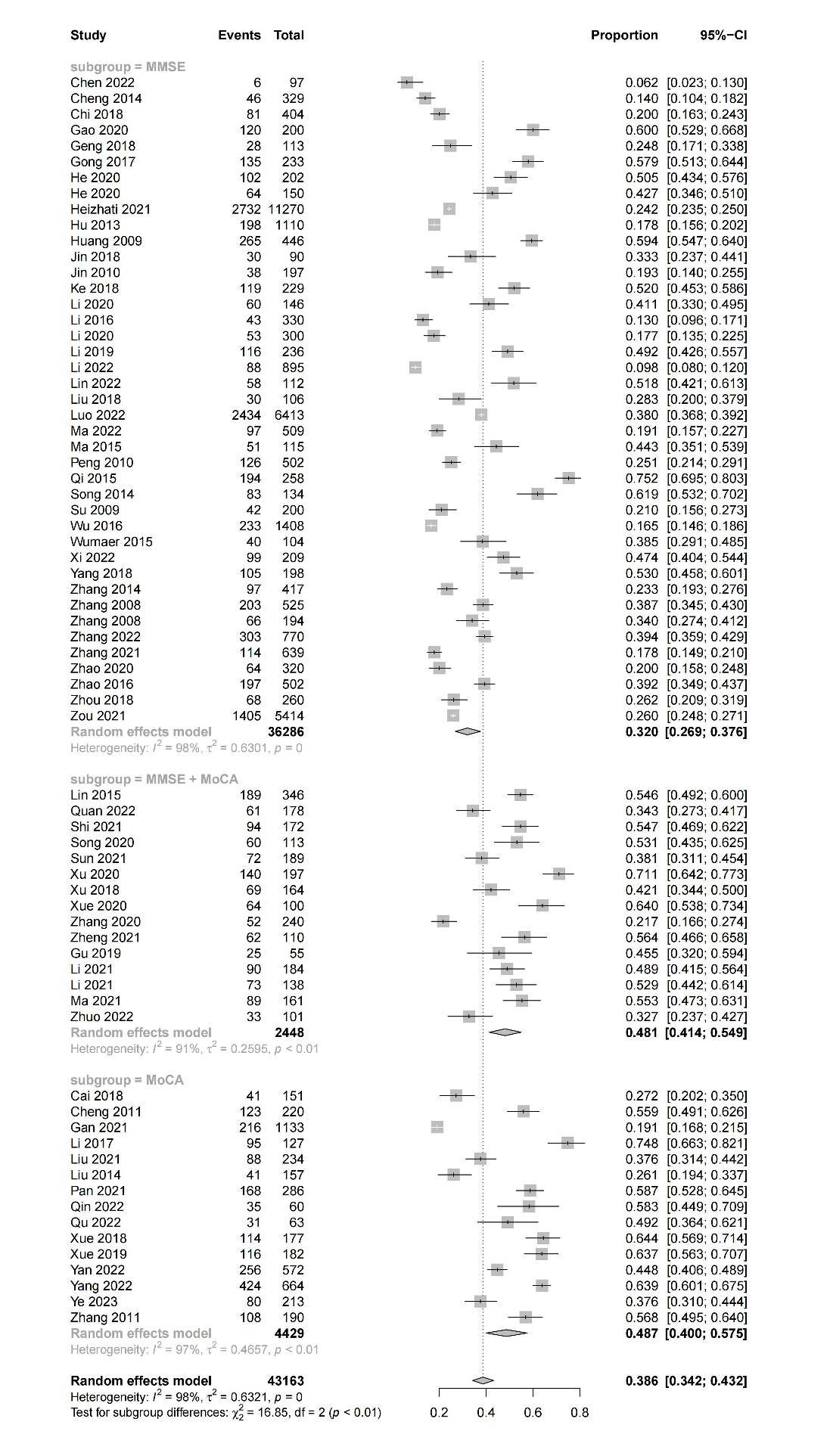


**Supplementary Figure 2.** **The forest plot of prevalence of cognitive impairment in Chinese hypertensive patients based on the assessment tools of cognitive impairment**


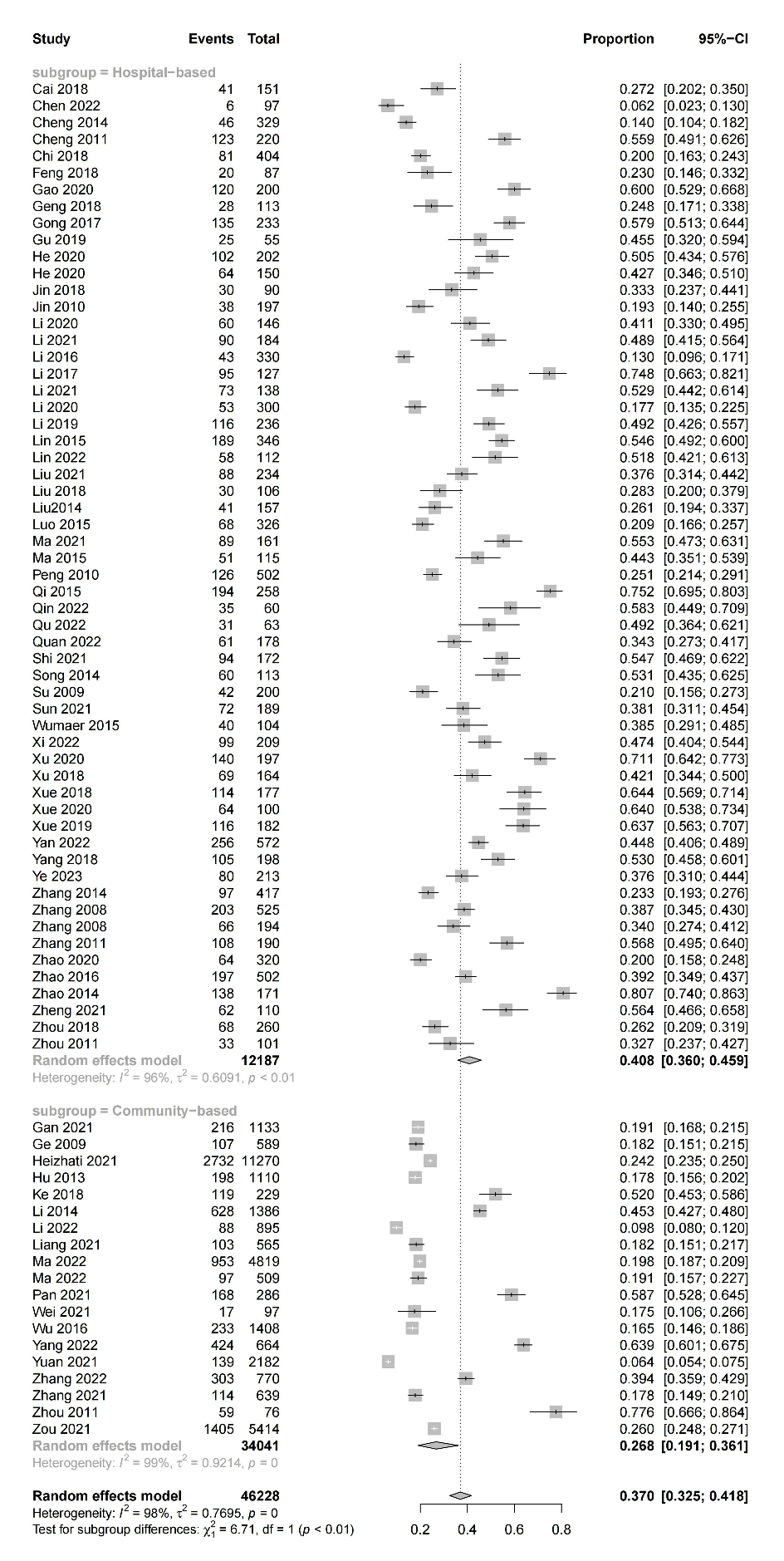


**Supplementary Figure 3.** **The forest plot of prevalence of cognitive impairment in Chinese hypertensive patients based on the recruitment source**


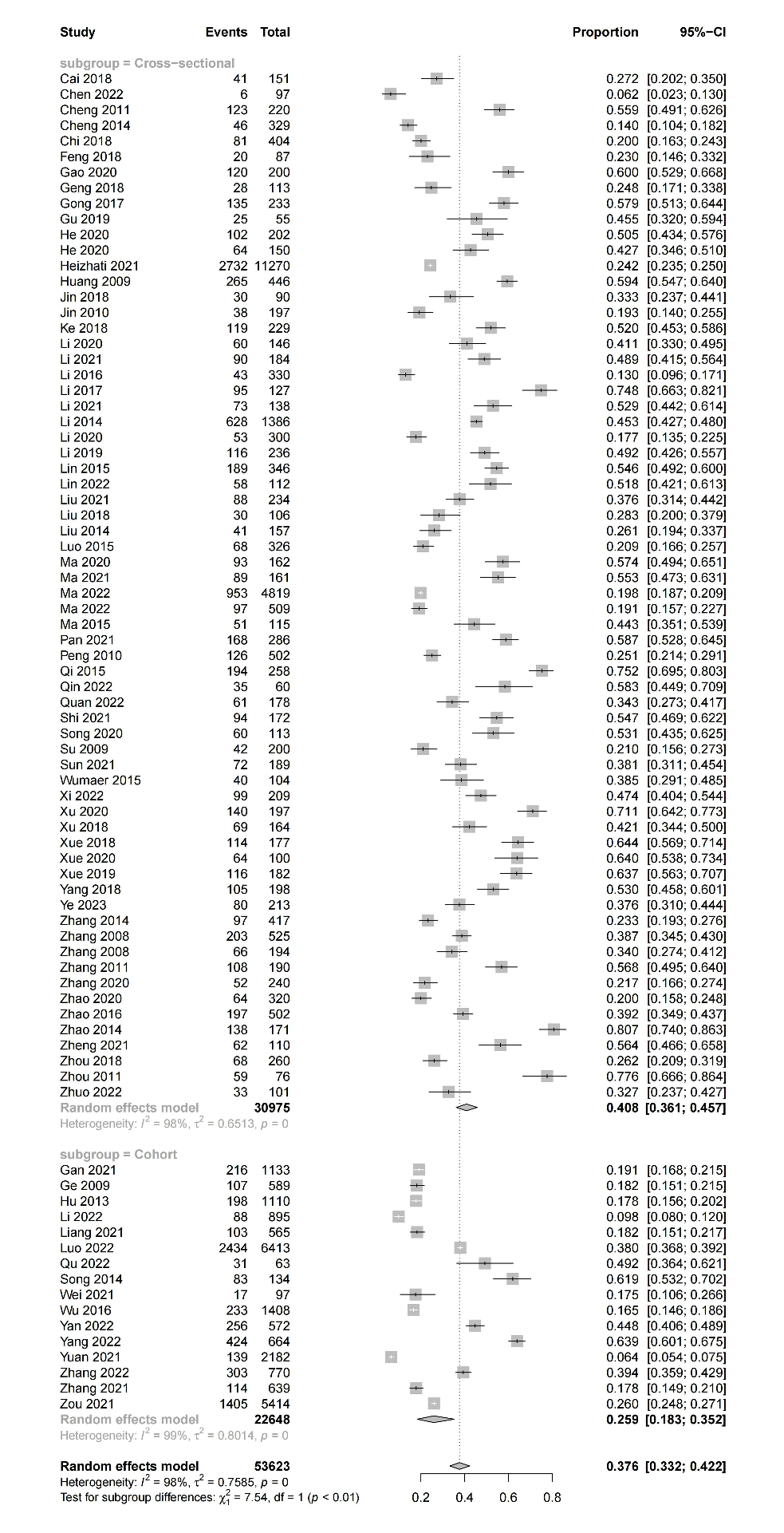


**Supplementary Figure 4.** **The forest plot of prevalence of cognitive impairment in Chinese hypertensive patients based on the study design**


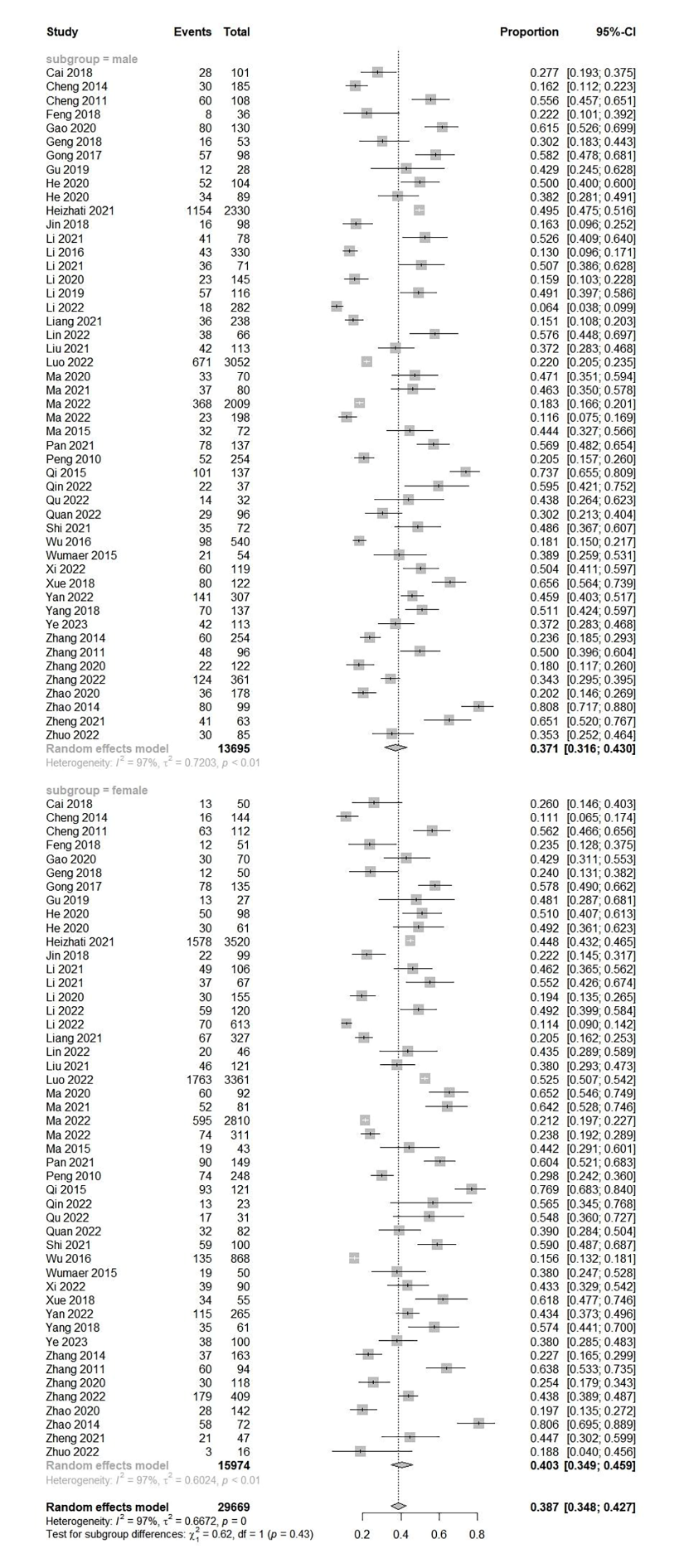


**Supplementary Figure 5A.** **The forest plot of prevalence of cognitive impairment in Chinese hypertensive patients based on the gender**


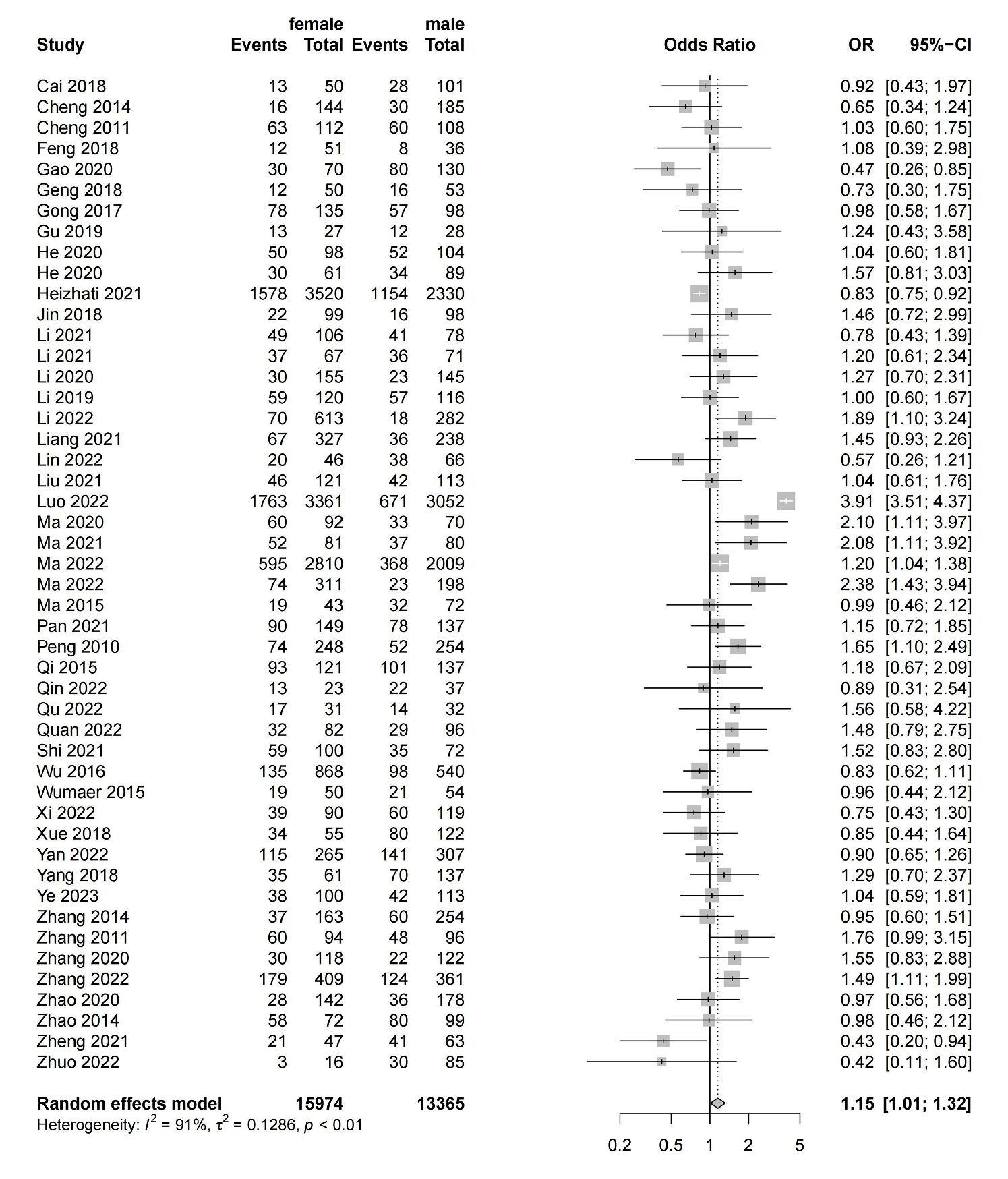


**Supplementary Figure 5B.** **The forest plot of OR of cognitive impairment in Chinese hypertensive patients based on the gender (female vs. male)**


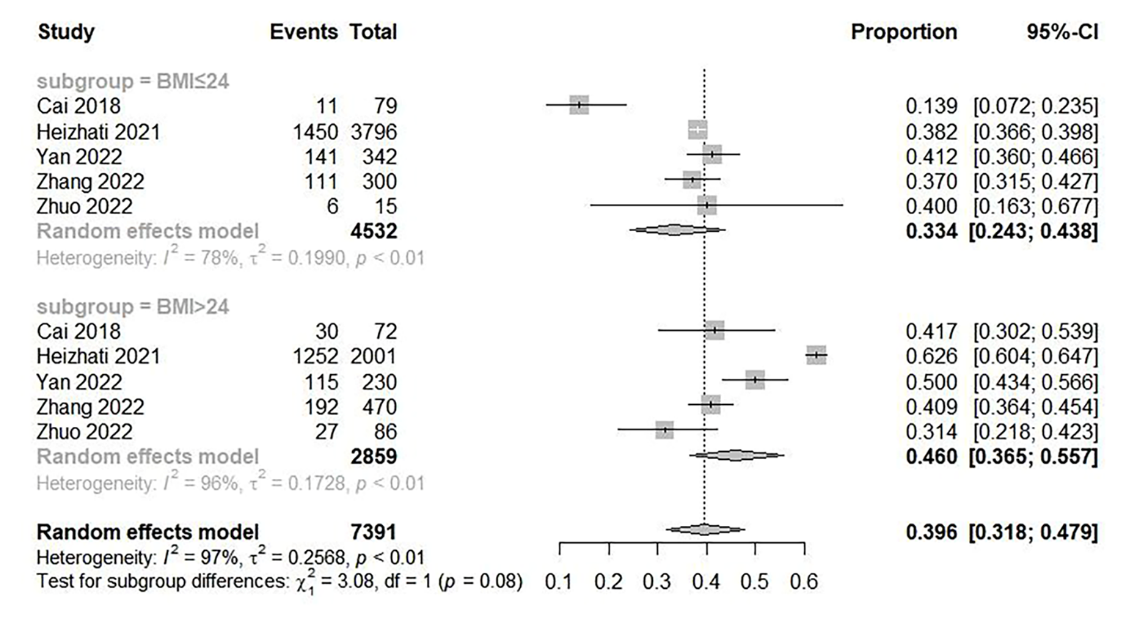


**Supplementary Figure 6A.** **The forest plot of prevalence of cognitive impairment in Chinese hypertensive patients based on the weight status**


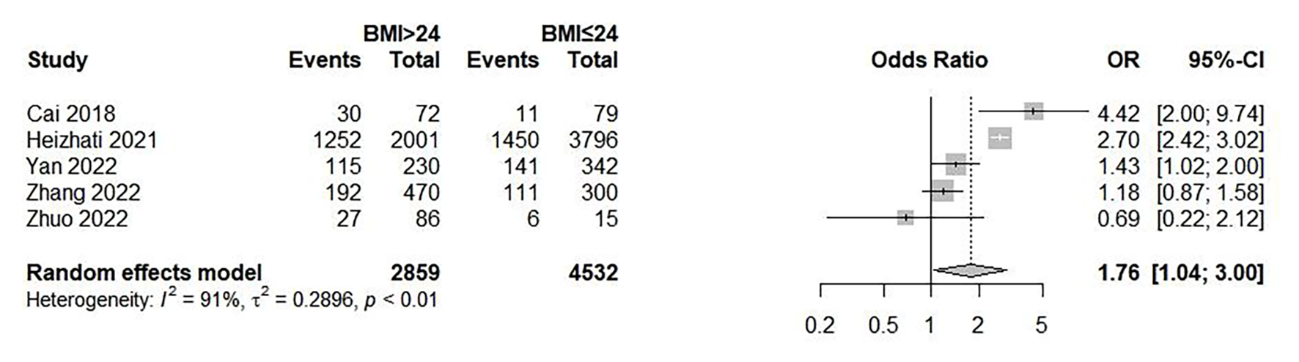


**Supplementary Figure 6B.** **The forest plot of OR of cognitive impairment in Chinese hypertensive patients based on the weight status (BMI>24 vs. BMI≤24)**


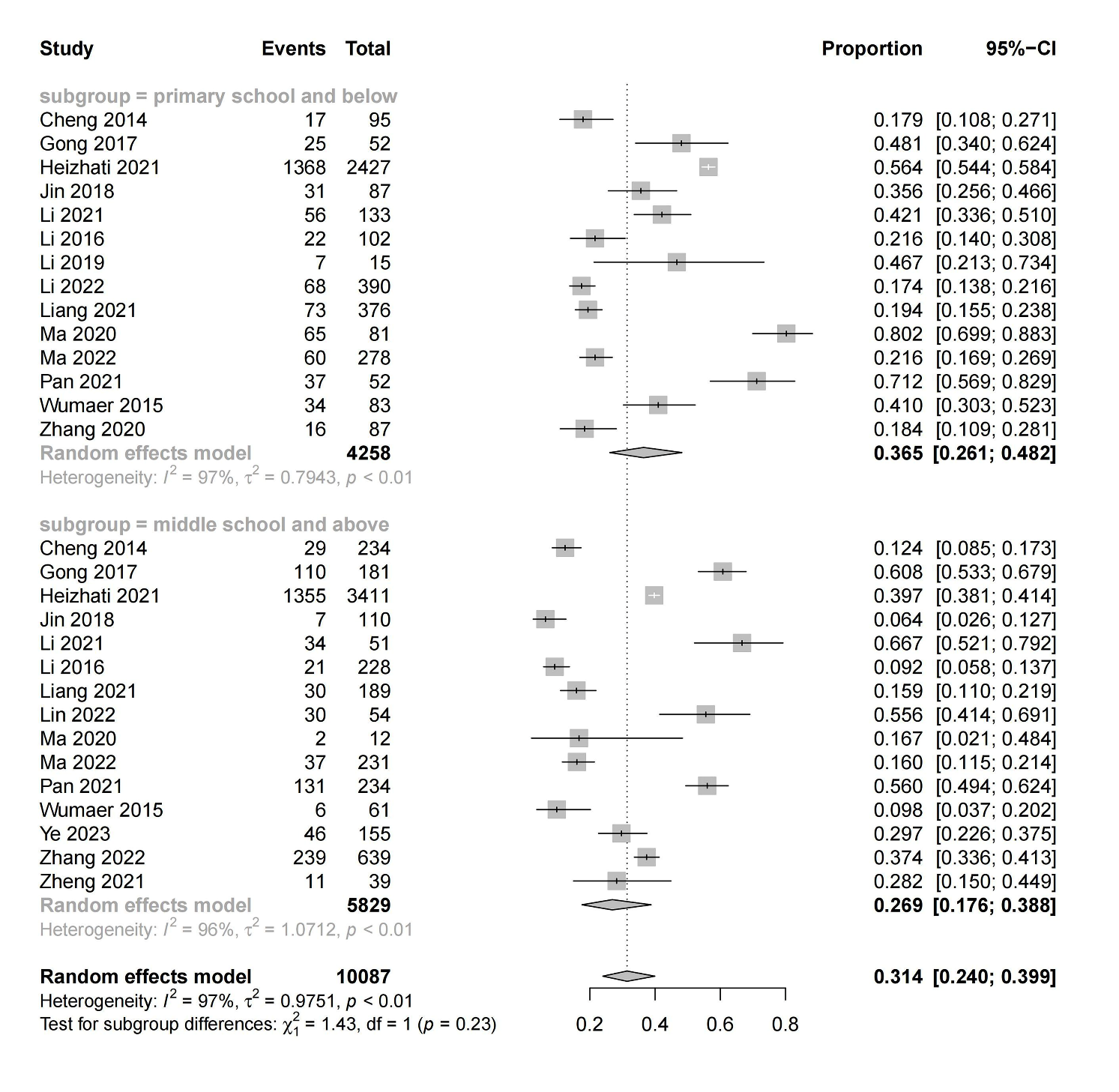


**Supplementary Figure 7A.** **The forest plot of prevalence of cognitive impairment in Chinese hypertensive patients based on the education level**


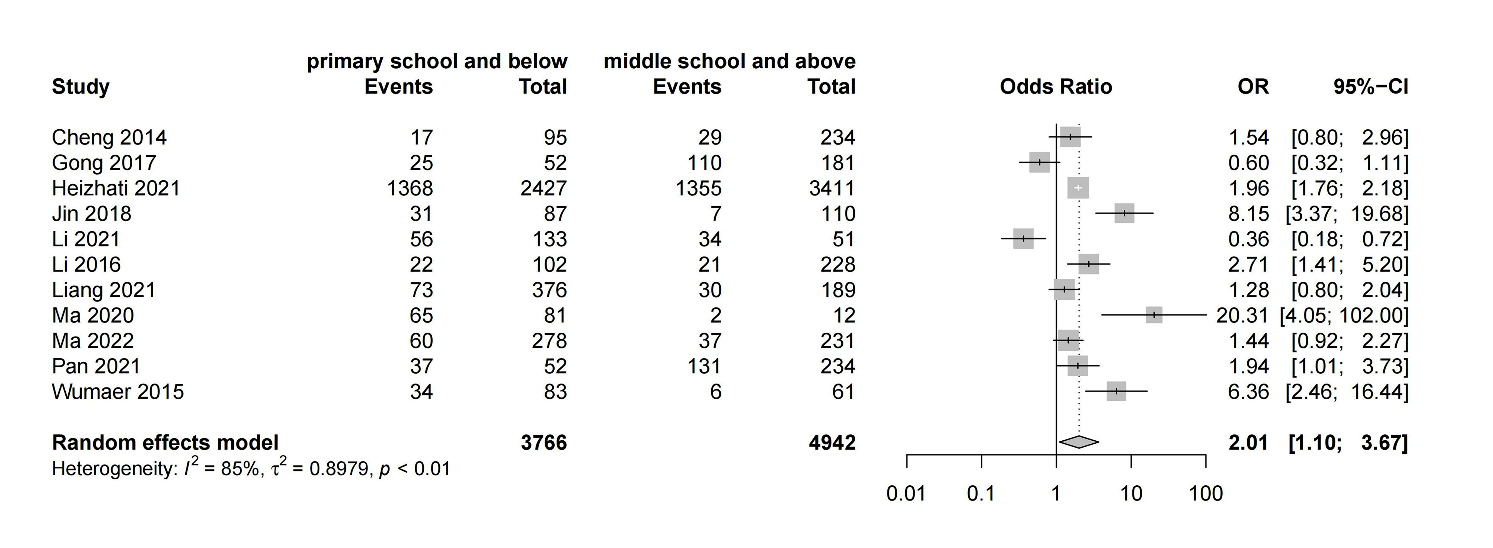


**Supplementary Figure 7B.** **The forest plot of OR of cognitive impairment in Chinese hypertensive patients based on the education level (primary school or below vs. middle school and above)**


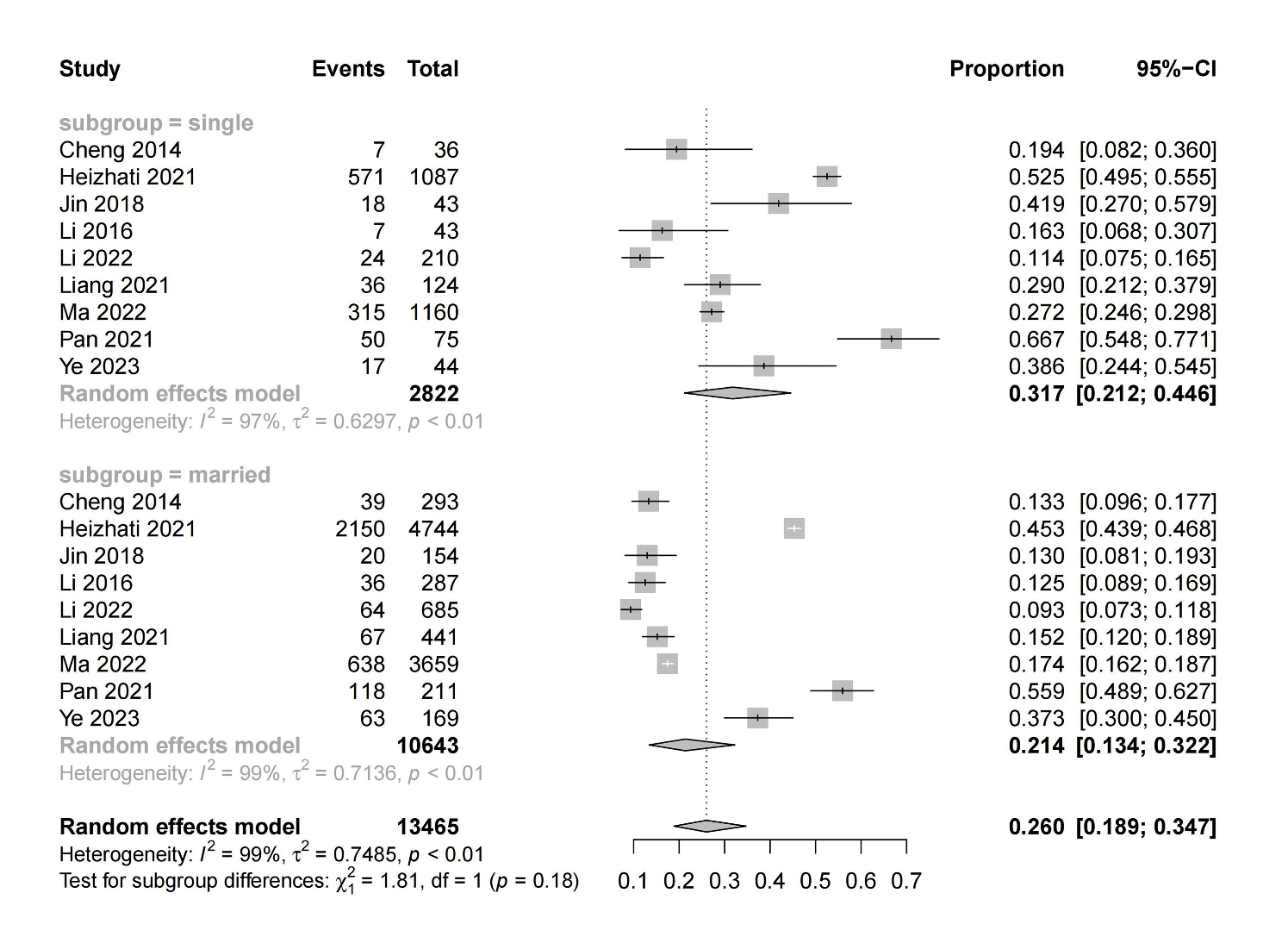


**Supplementary Figure 8A.** **The forest plot of prevalence of cognitive impairment in Chinese hypertensive patients based on the marital status**


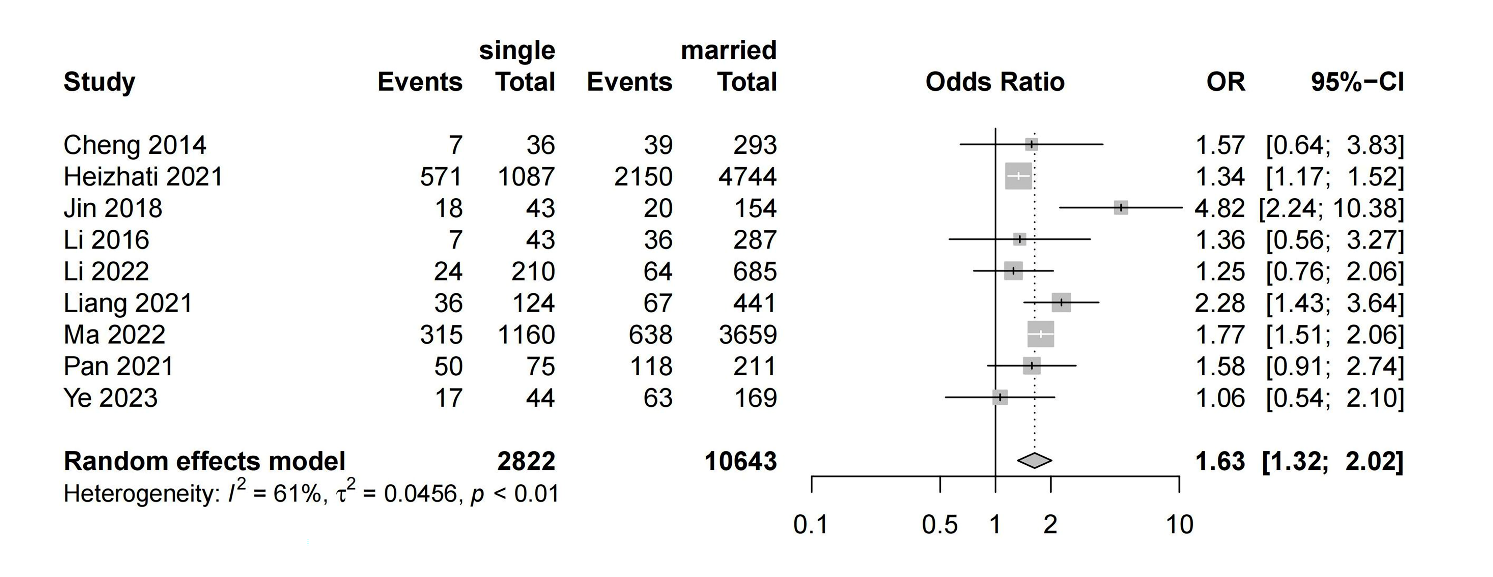


**Supplementary Figure 8B.** **The forest plot of OR of cognitive impairment in Chinese hypertensive patients based on the marital status (single vs. married)**


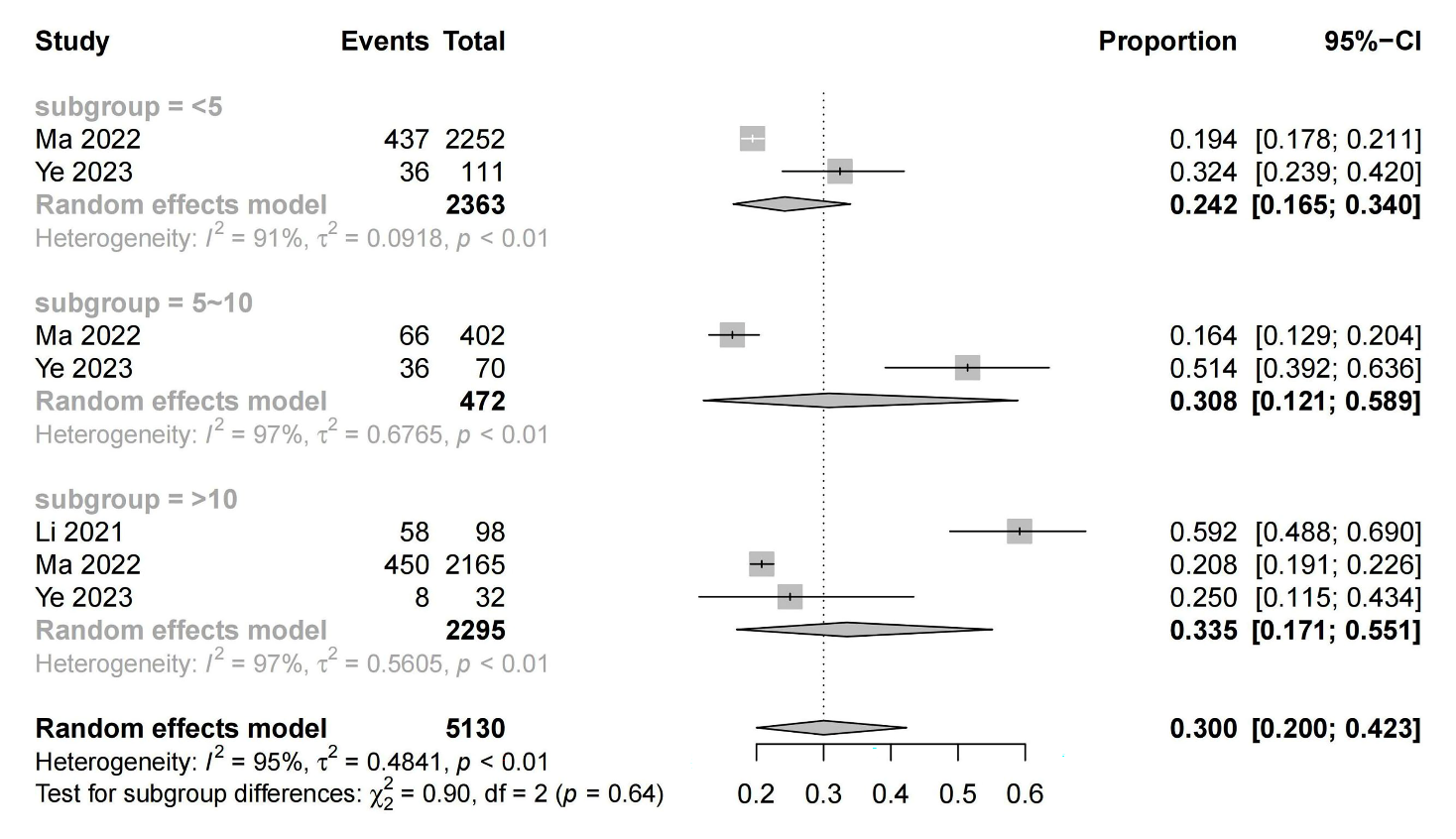


**Supplementary Figure 9A.** **The forest plot of prevalence of cognitive impairment in Chinese hypertensive patients based on the duration of hypertension**


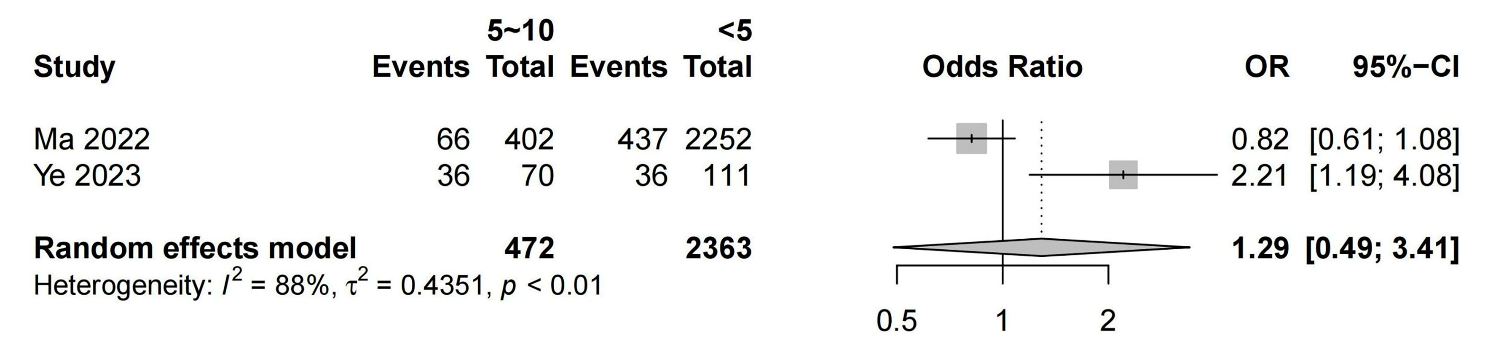


**Supplementary Figure 9B.** **The forest plot of OR of cognitive impairment in Chinese hypertensive patients based on the duration of hypertension (5~10 vs. <5)**


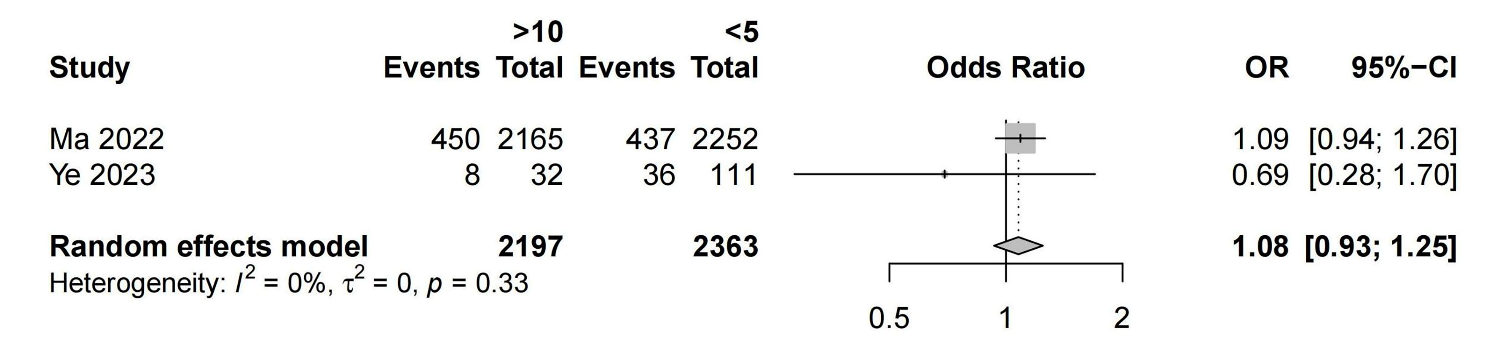


**Supplementary Figure 9B.** **The forest plot of OR of cognitive impairment in Chinese hypertensive patients based on the duration of hypertension (>10 vs. <5)**


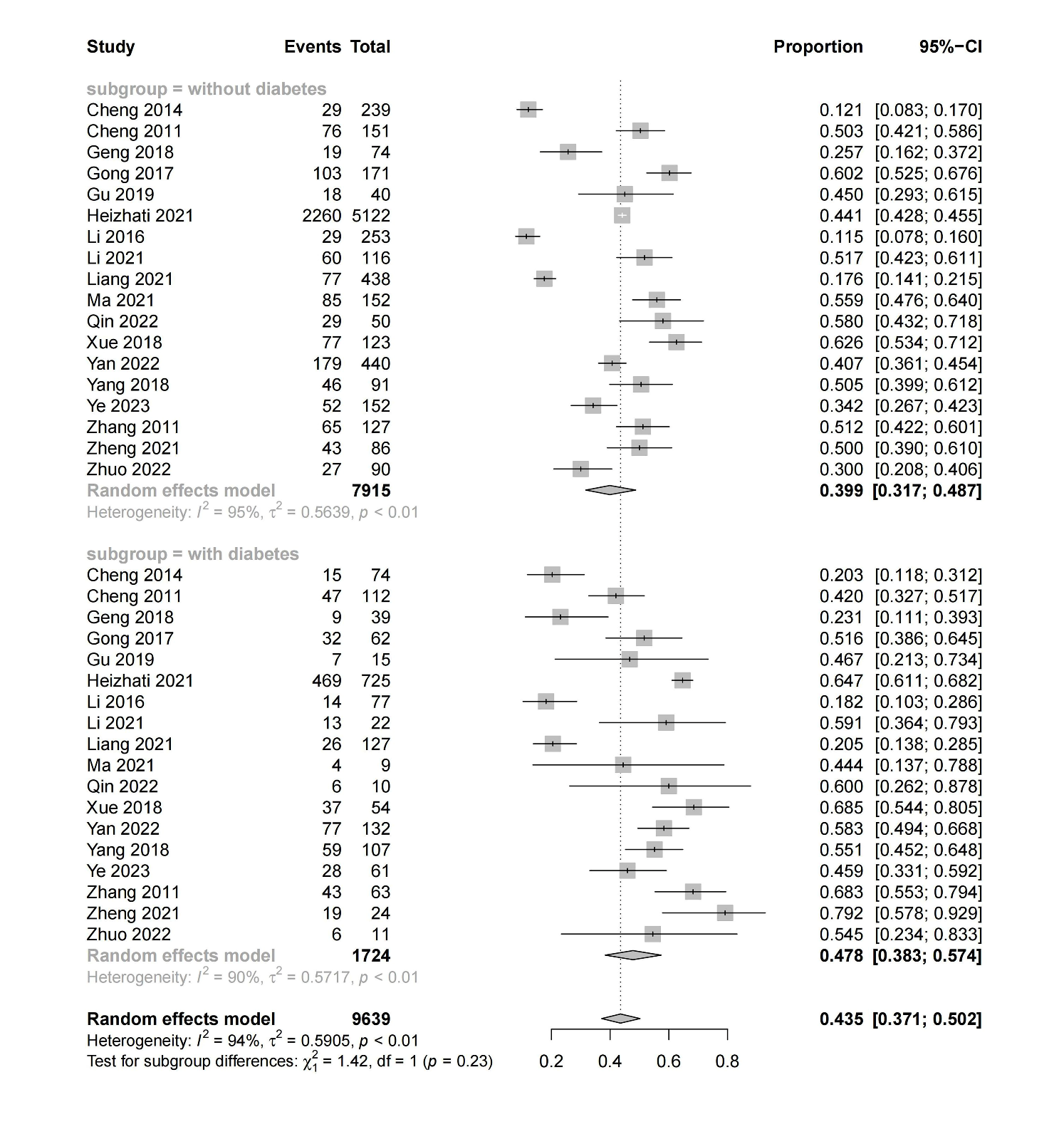


**Supplementary Figure 10A.** **The forest plot of prevalence of cognitive impairment in Chinese hypertensive patients based on the diabetes complication**


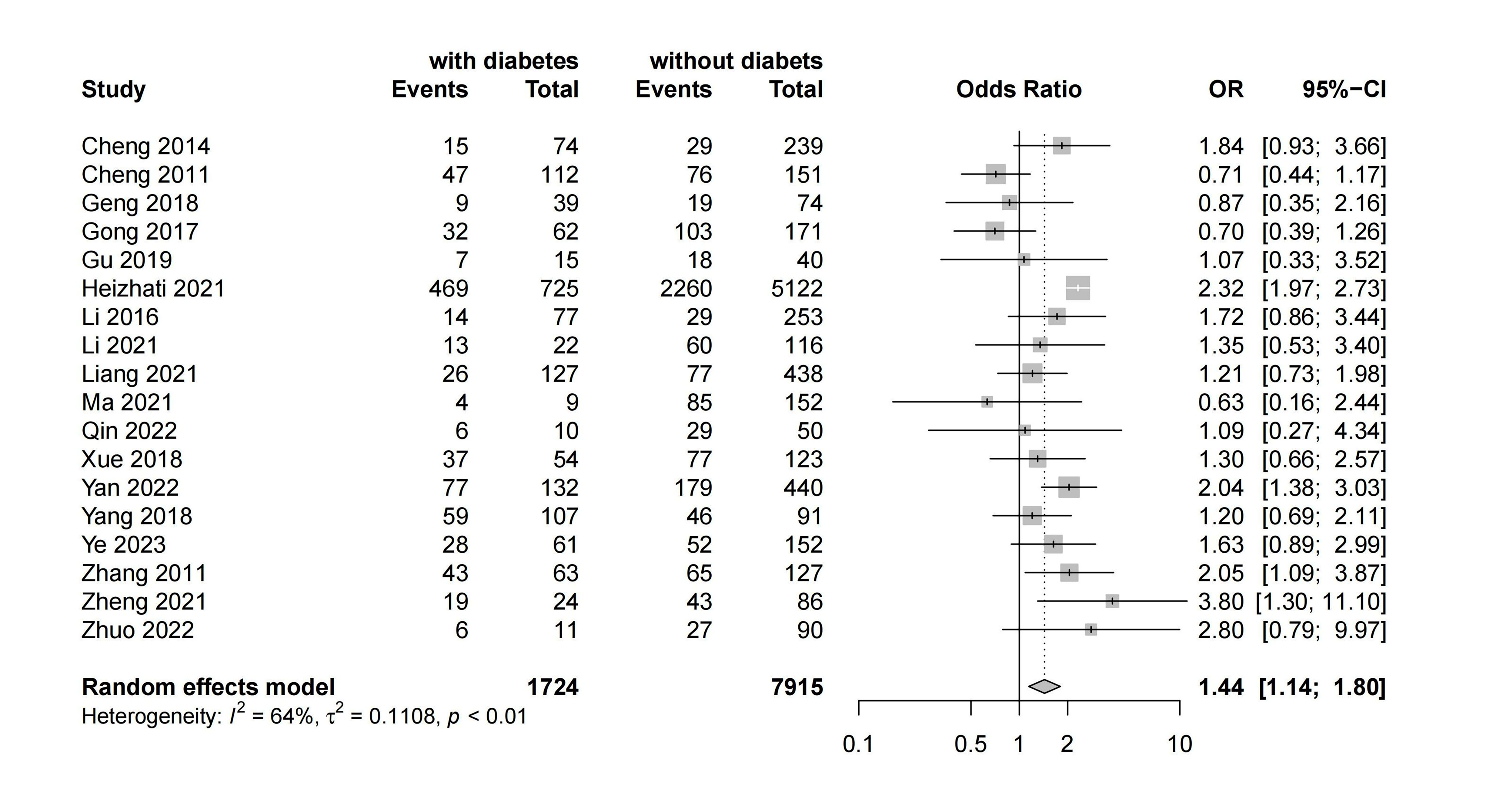


**Supplementary Figure 10B.** **The forest plot of OR of cognitive impairment in Chinese hypertensive patients based on the diabetes complication (with diabetes vs. without diabetes)**


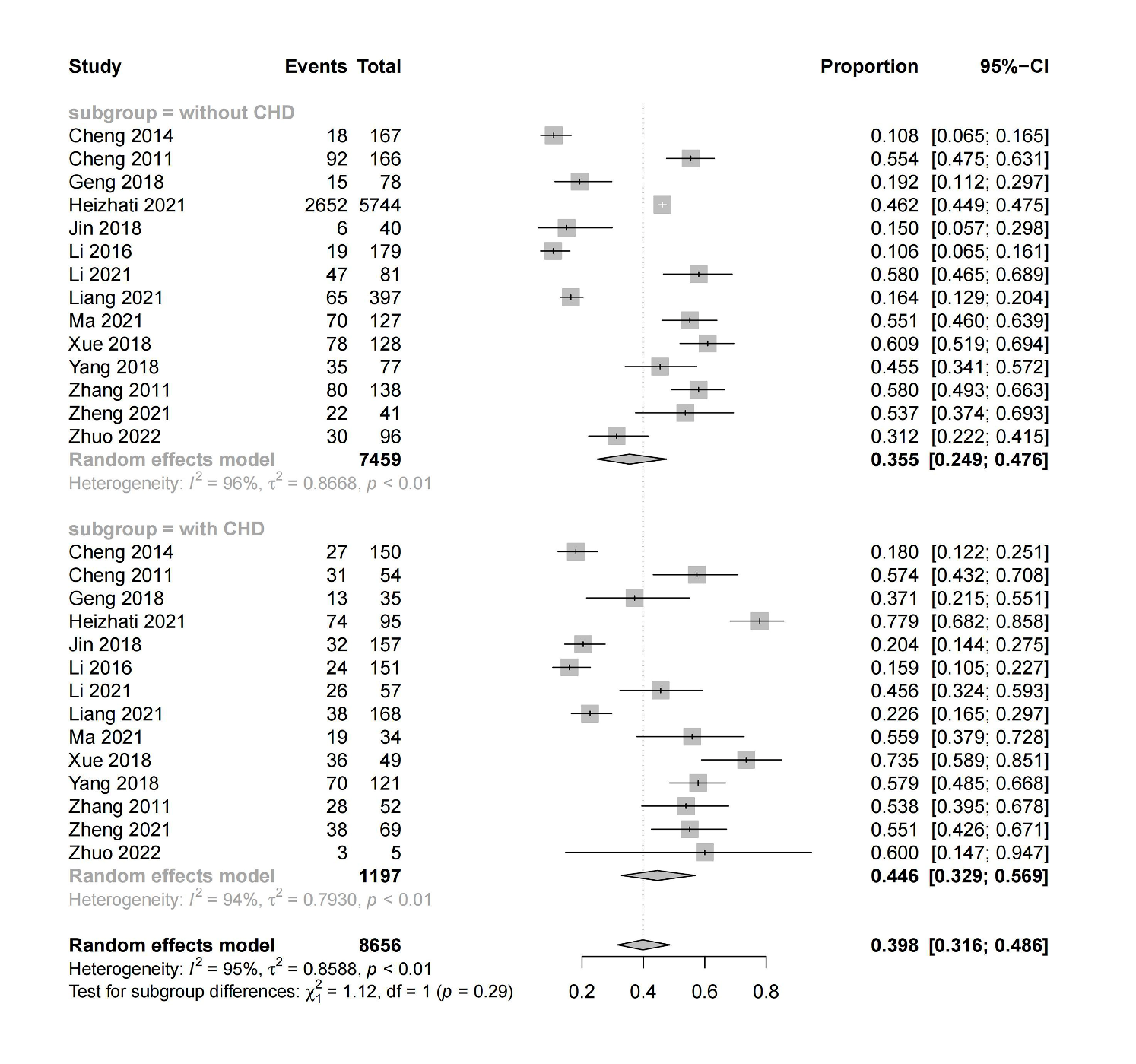


**Supplementary Figure 10C.** **The forest plot of prevalence of cognitive impairment in Chinese hypertensive patients based on the CHD complication**


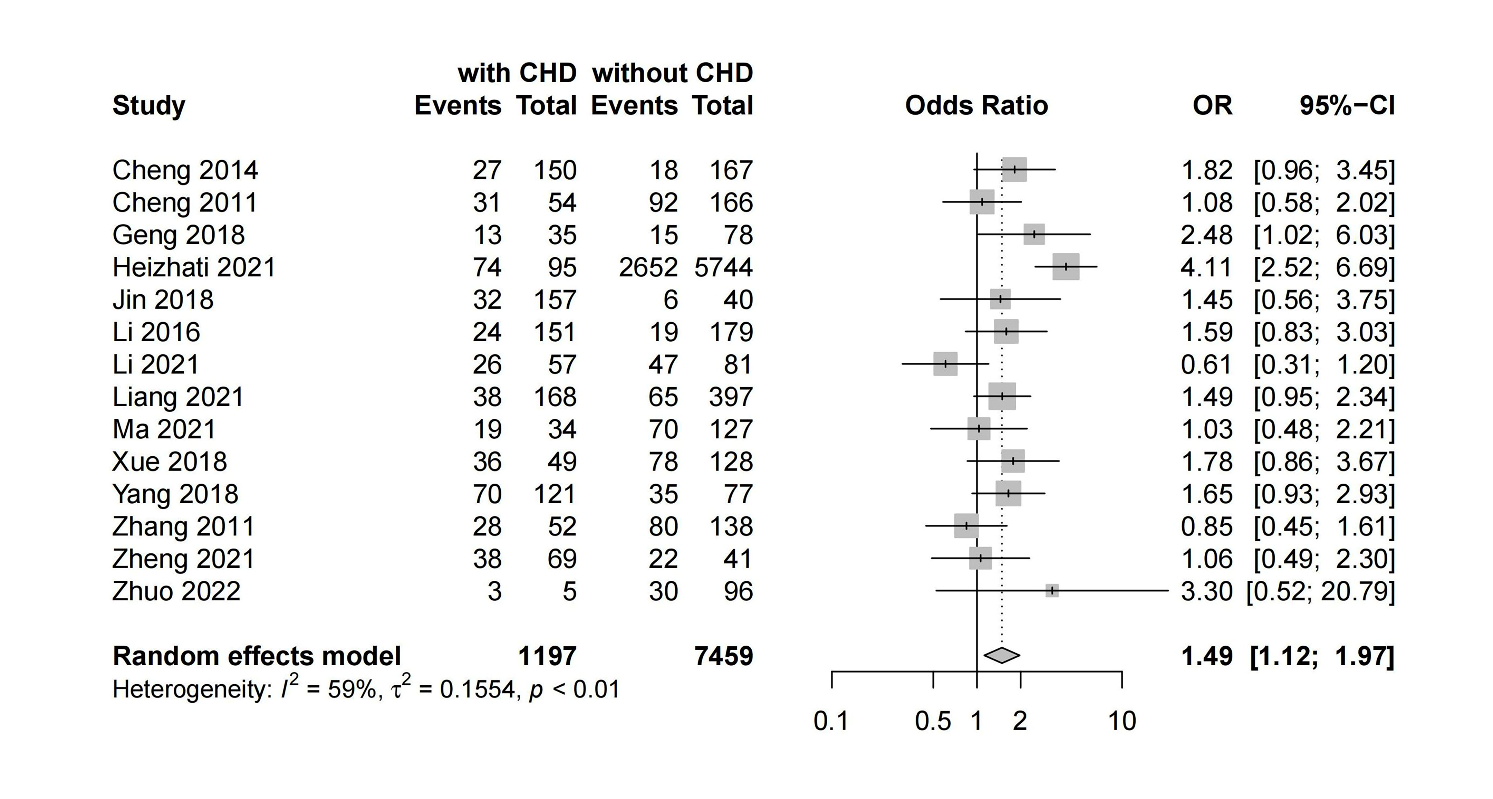


**Supplementary Figure 10D.** **The forest plot of OR of cognitive impairment in Chinese hypertensive patients based on the CHD complication (with CHD vs. without CHD)**


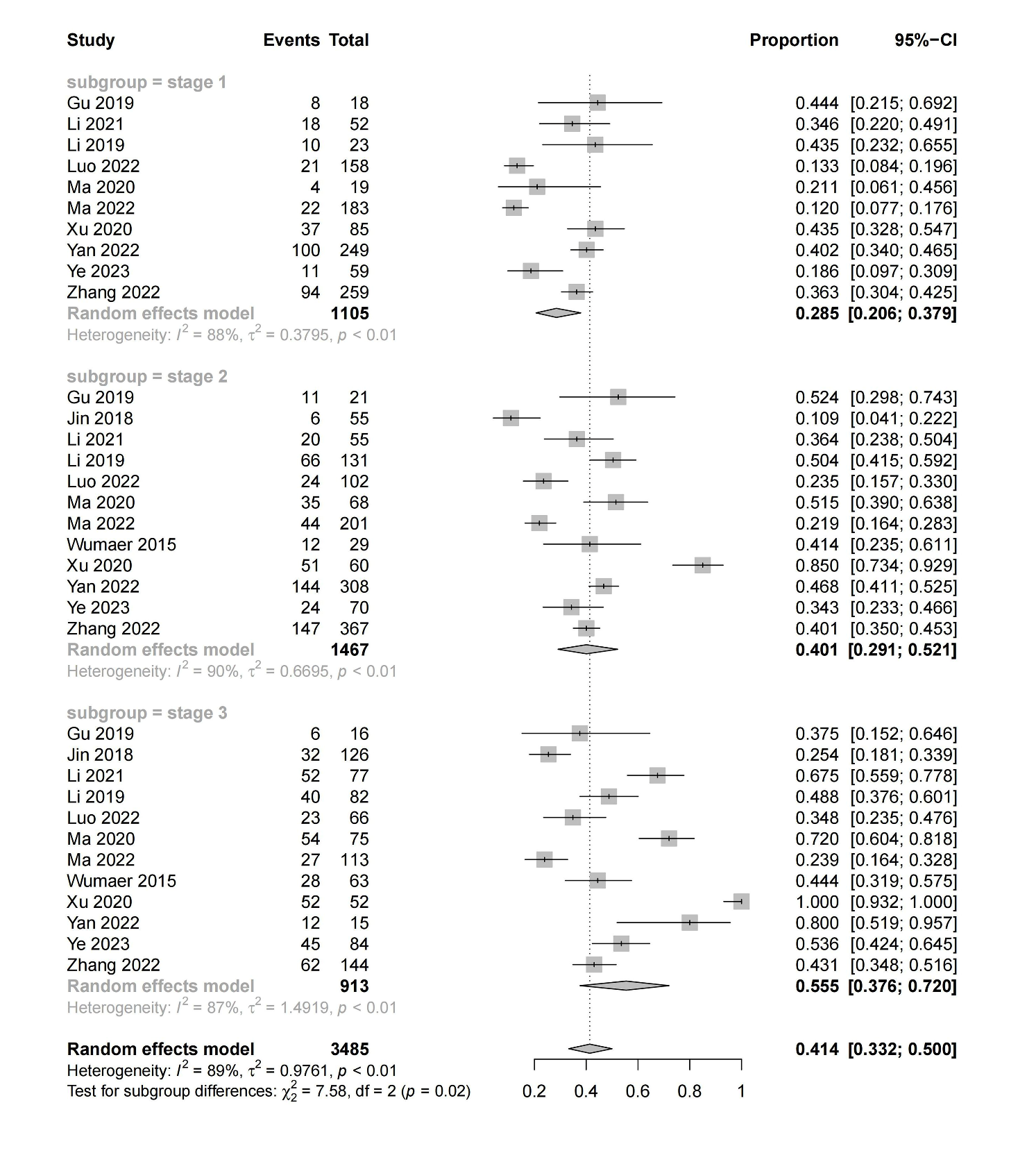


**Supplementary Figure 11A.** **The forest plot of prevalence of cognitive impairment in Chinese hypertensive patients based on the** **classification of hypertension**


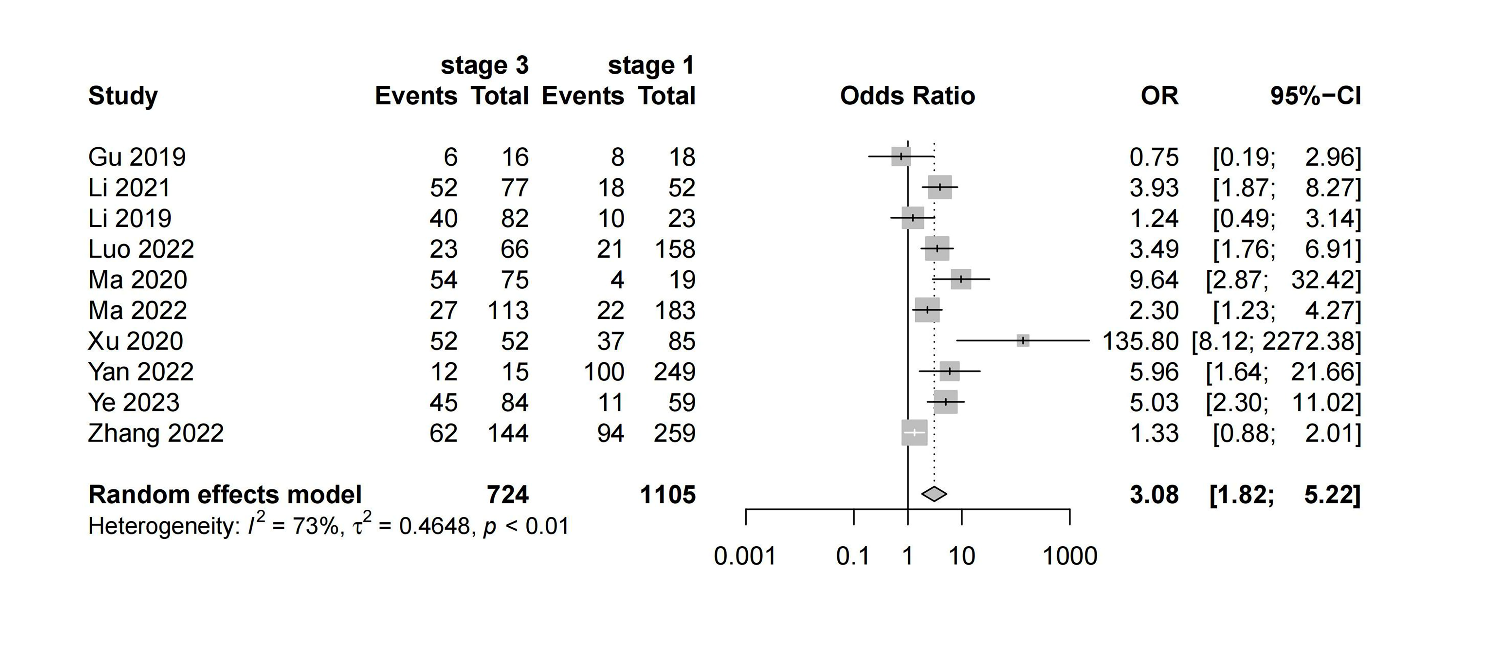


**Supplementary Figure 11B.** **The forest plot of OR of cognitive impairment in Chinese hypertensive patients based on the classification of hypertension (stage 3 vs. stage 1)**


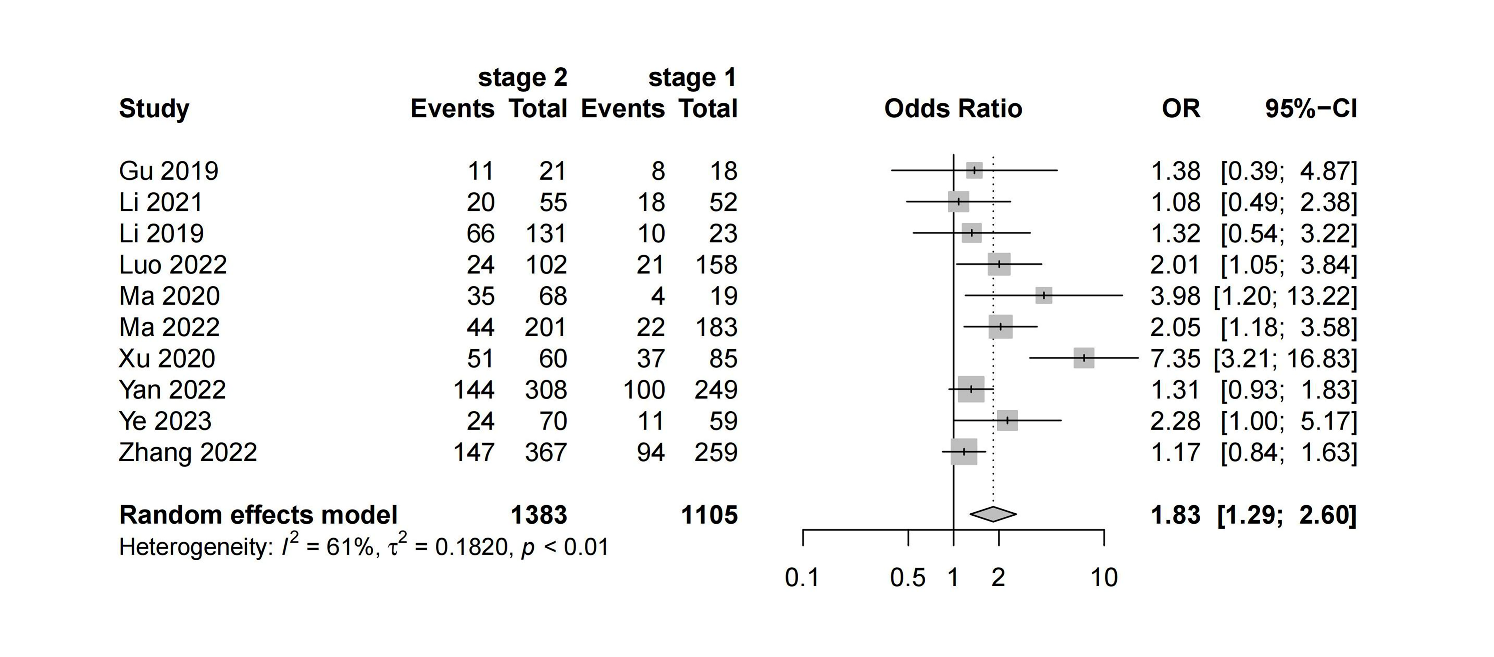


**Supplementary Figure 11C.** **The forest plot of OR of cognitive impairment in Chinese hypertensive patients based on the classification of hypertension (stage 2 vs. stage 1)**


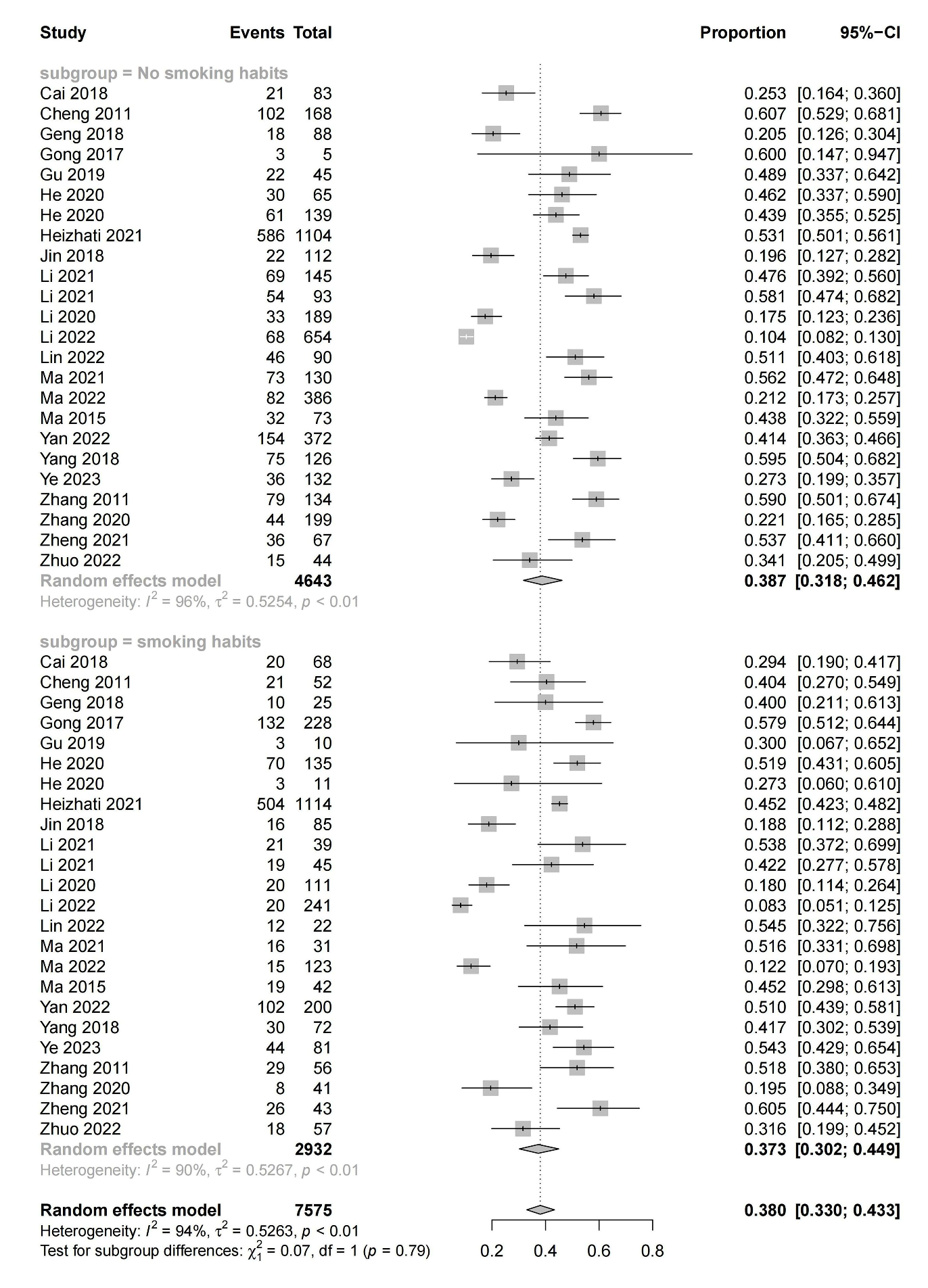


**Supplementary Figure 12A.** **The forest plot of prevalence of cognitive impairment in Chinese hypertensive patients based on the smoking habits**


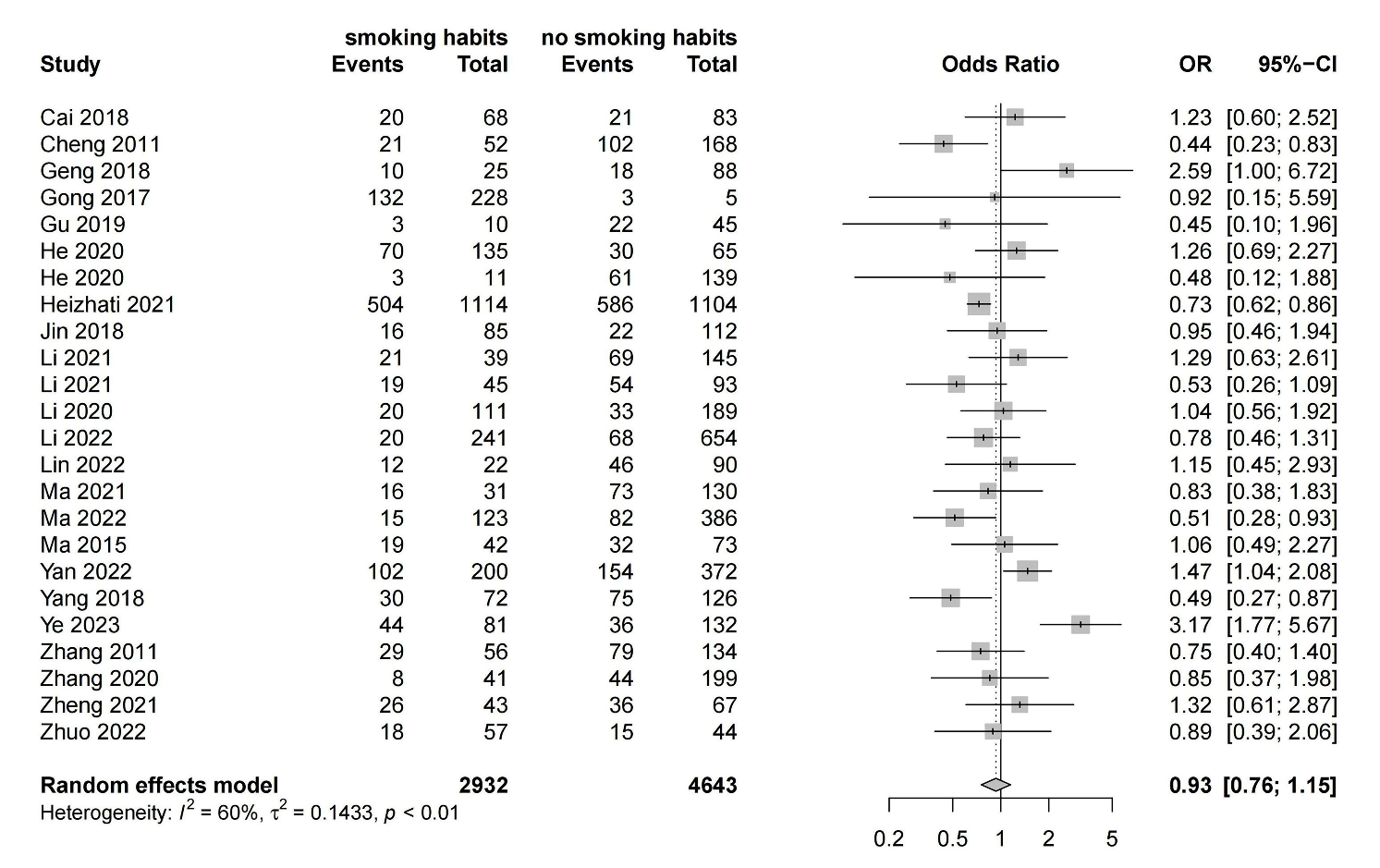


**Supplementary Figure 12B.** **The forest plot of OR of cognitive impairment in Chinese hypertensive patients based on the smoking habits (smoking habits vs. no smoking habits)**


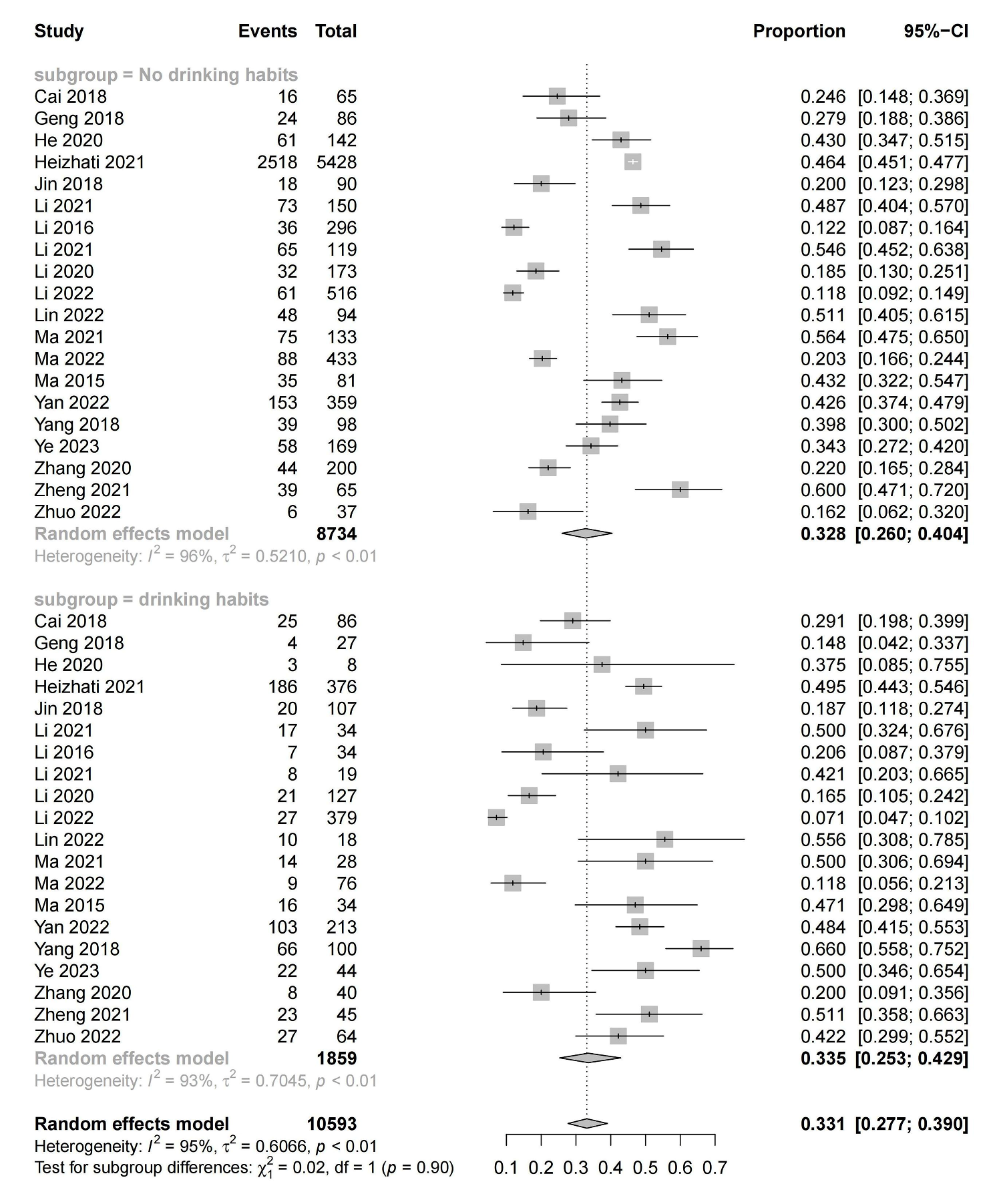


**Supplementary Figure 13A.** **The forest plot of prevalence of cognitive impairment in Chinese hypertensive patients based on the drinking habits**


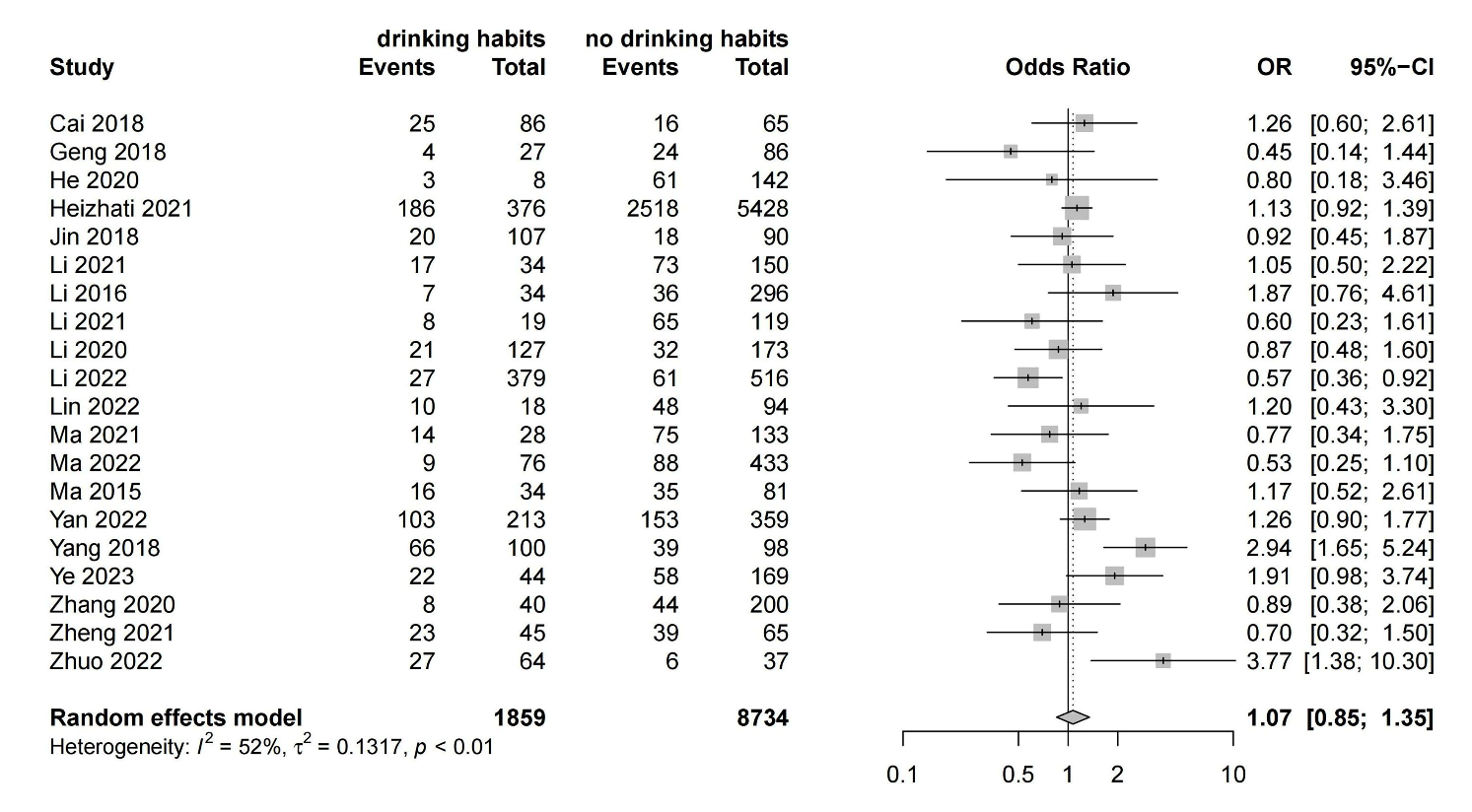


**Supplementary Figure 13B.** **The forest plot of OR of cognitive impairment in Chinese hypertensive patients based on the smoking habits (drinking habits vs. no drinking habits)**


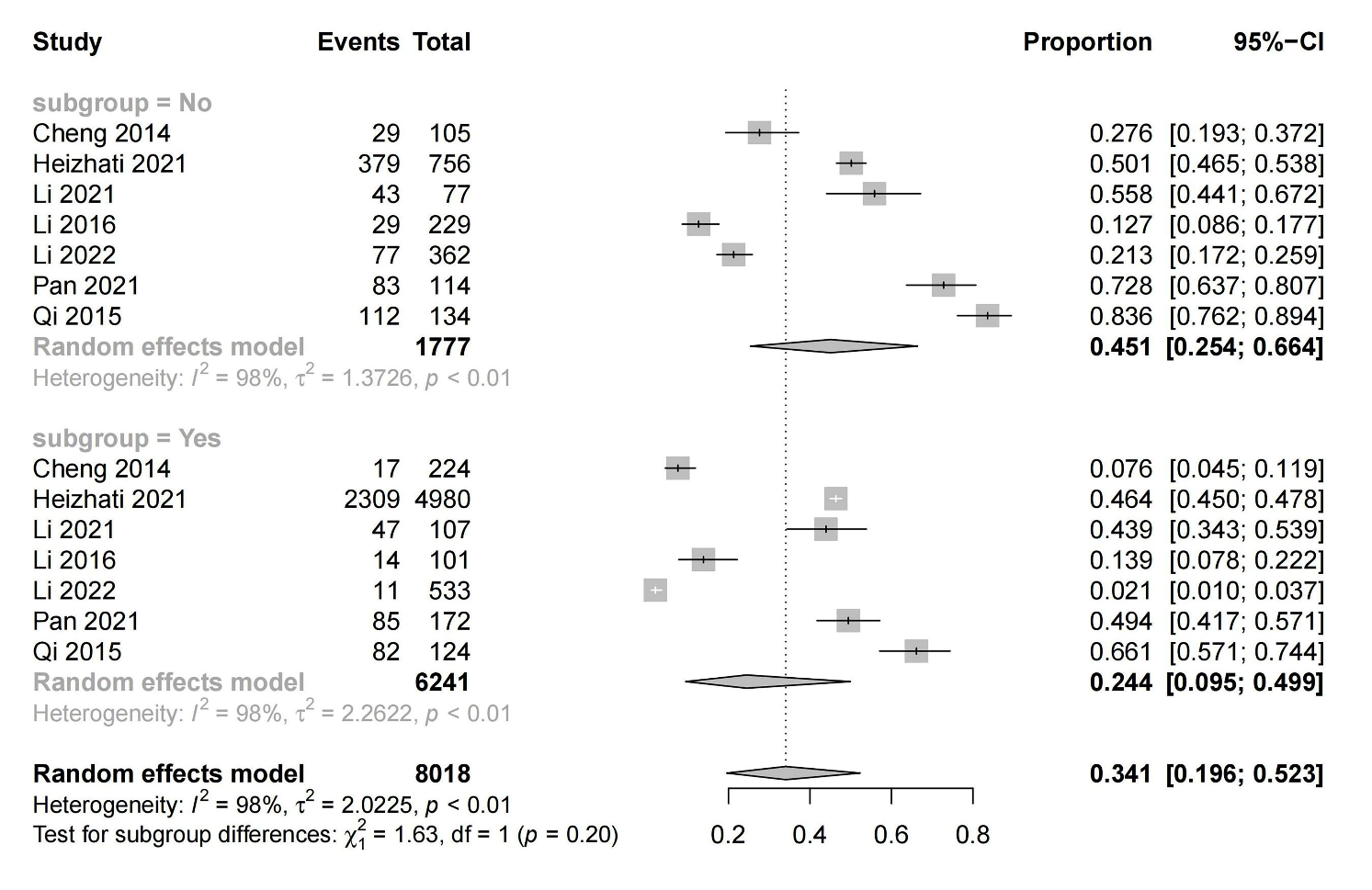


**Supplementary Figure 14A.** **The forest plot of prevalence of cognitive impairment in Chinese hypertensive patients based on the regular physical activity**


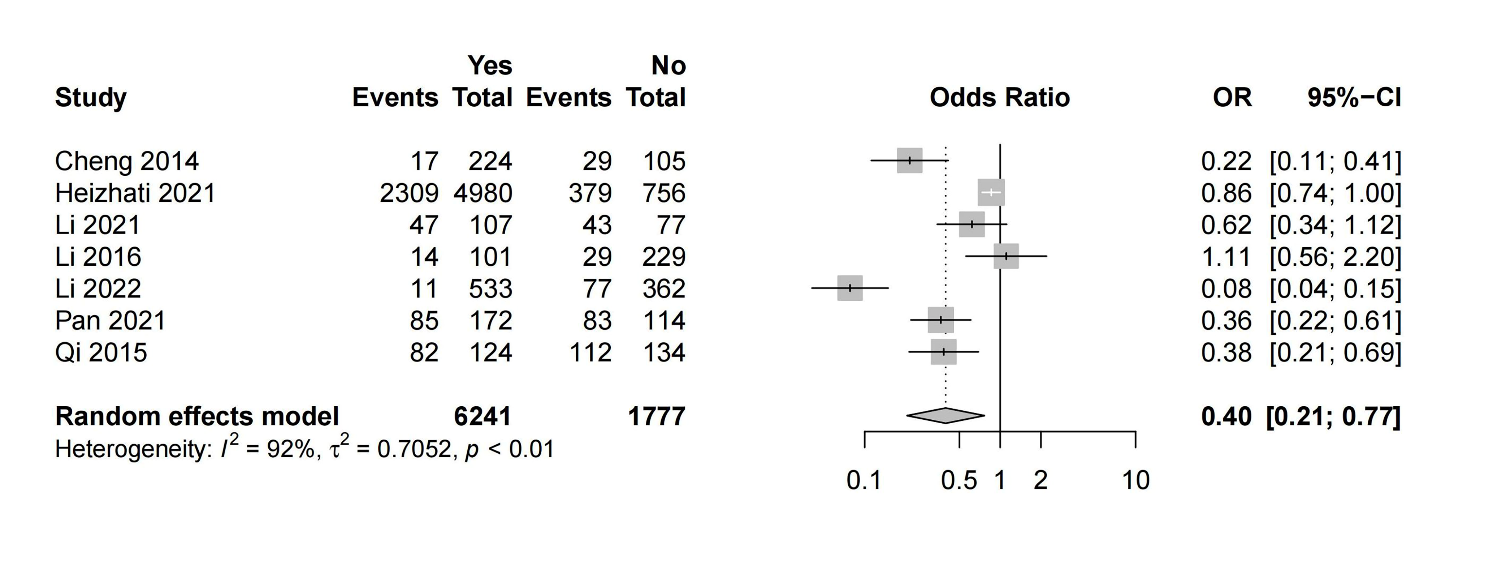


**Note: Yes: with regular physical activity; No: without regular physical activity**

**Supplementary Figure 14B.** **The forest plot of OR of cognitive impairment in Chinese hypertensive patients based on the regular physical activity (with regular physical activity vs. without regular physical activity)**


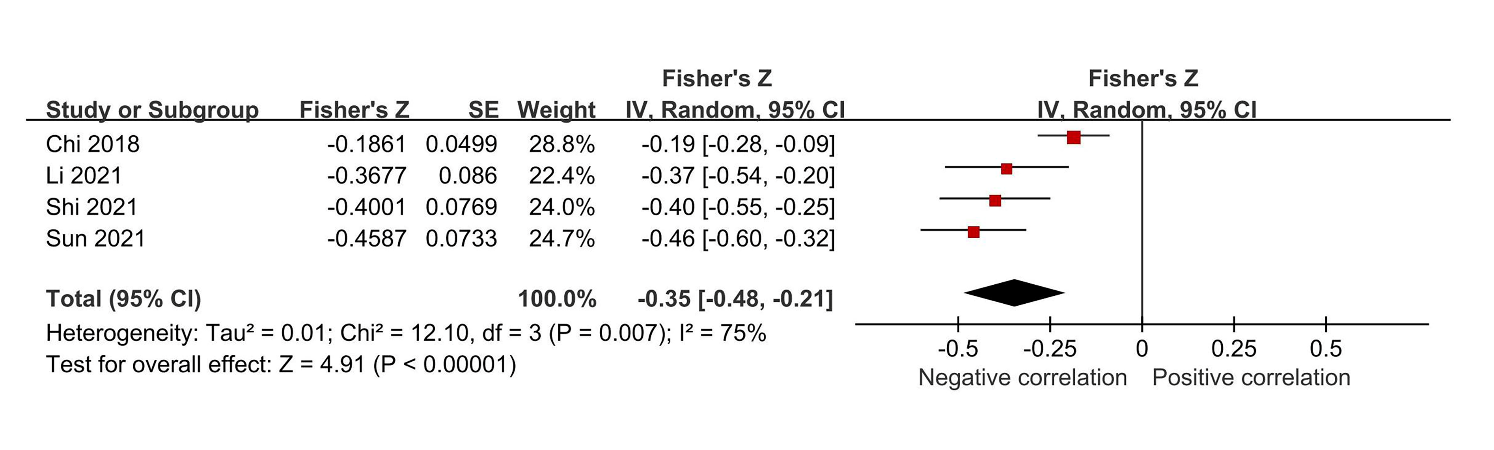


**Supplementary Figure 15.** **The forest plot of association between age and cognitive function in Chinese hypertensive patients**


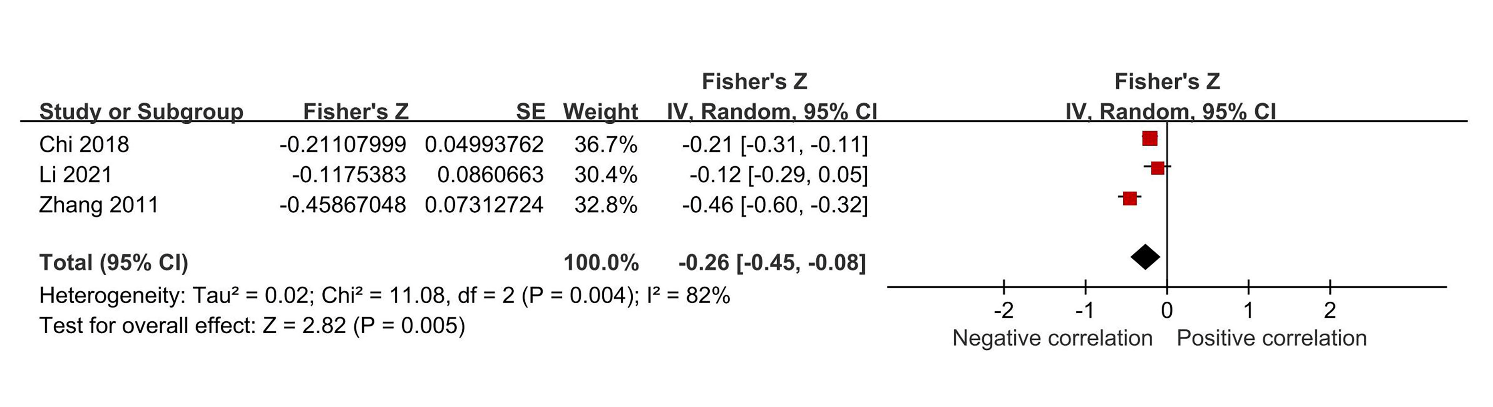


**Supplementary Figure 16.** **The forest plot of association between SBP and cognitive function in Chinese hypertensive patients**


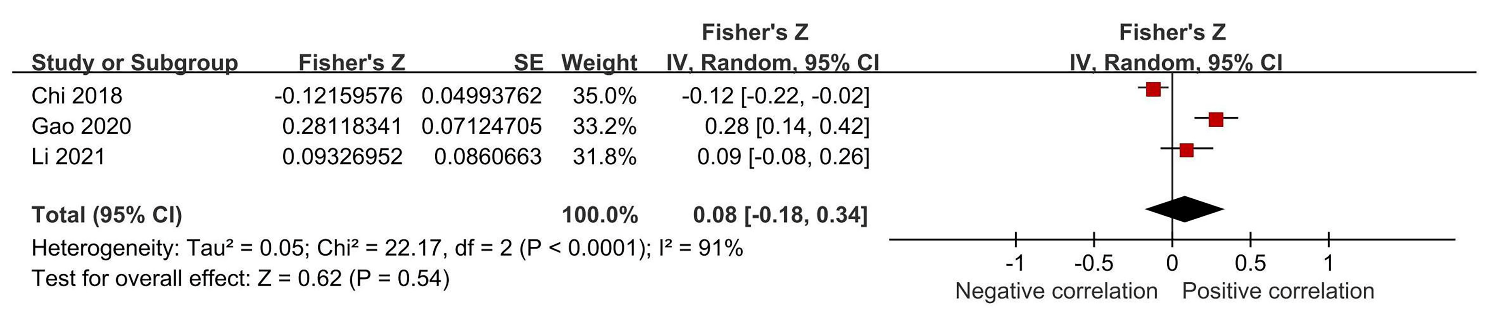


**Supplementary Figure 17.** **The forest plot of association between DBP and cognitive function in Chinese hypertensive patients**


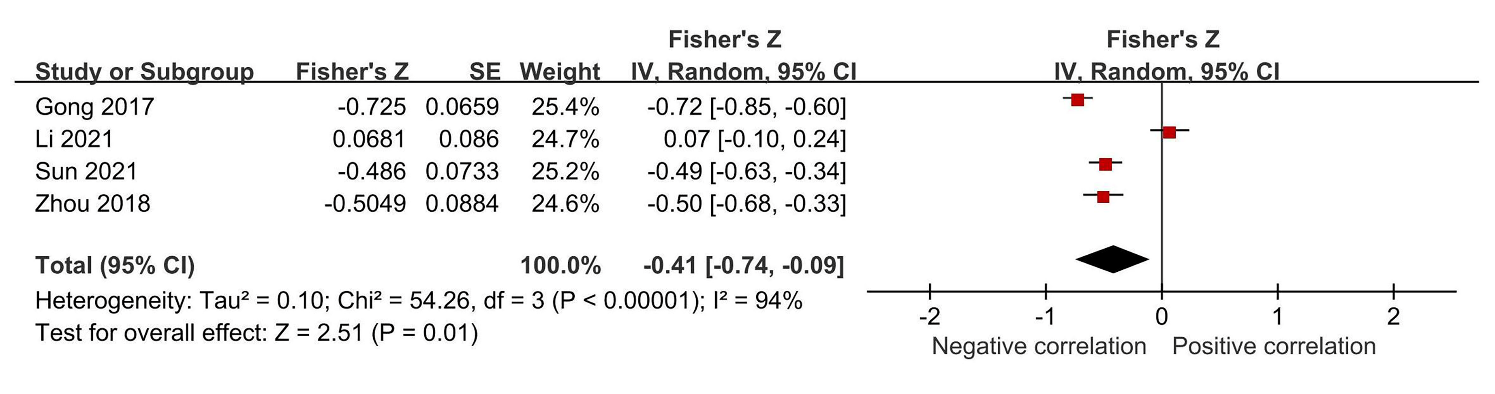


**Supplementary Figure 18.** **The forest plot of association between Hcy and cognitive function in Chinese hypertensive patients**


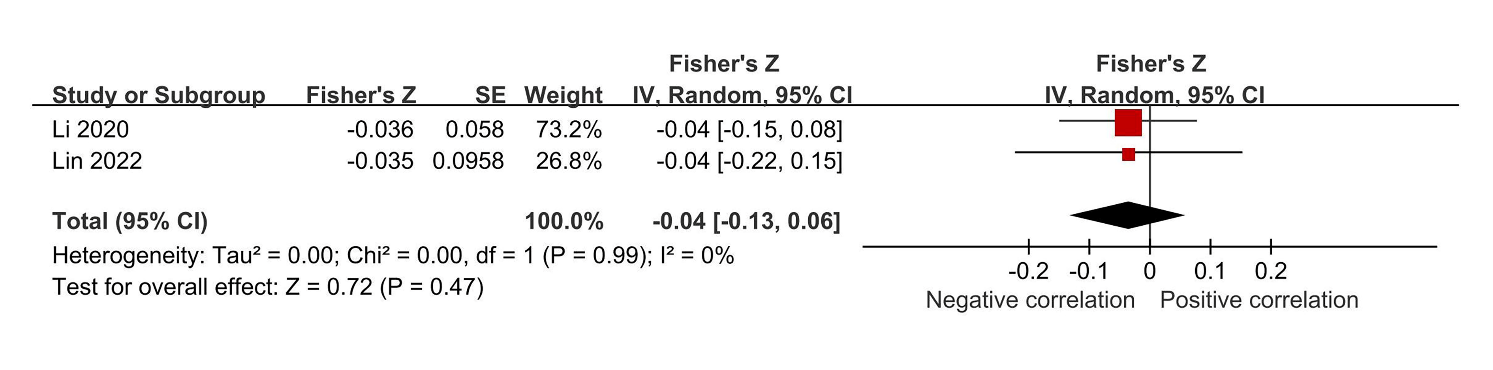


**Supplementary Figure 19.** **The forest plot of association between** **hs-CRP and cognitive function in Chinese hypertensive patients**


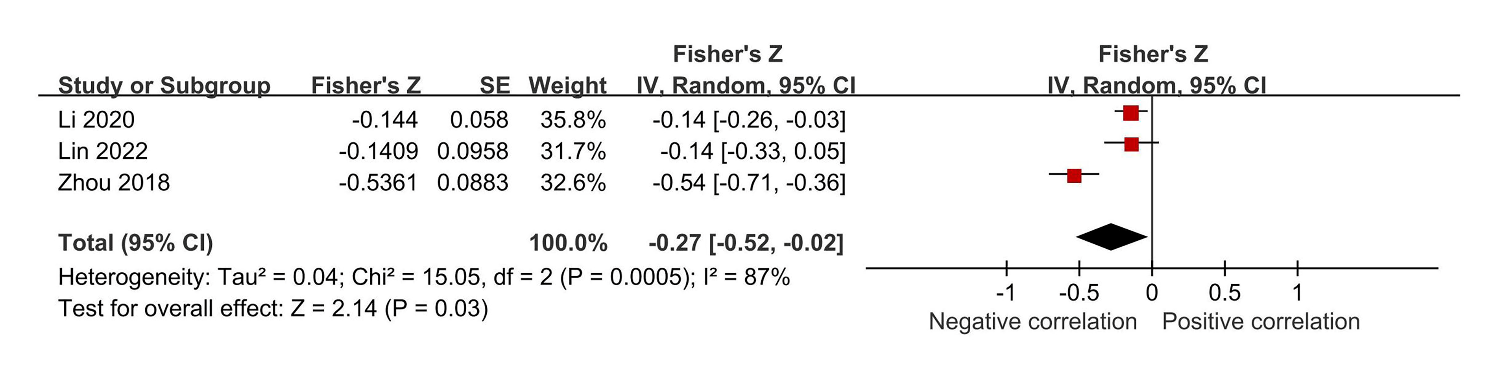


**Supplementary Figure 20.** **The forest plot of association between IL-6 and cognitive function in Chinese hypertensive patients**

## Supplementary Tables

**Supplementary Table 1.** **PRISMA checklist**

| **Section and Topic** | **Item #** | **Checklist item** | **Location where item is reported** |
| --- | --- | --- | --- |
| **TITLE** | | |  |
| Title | 1 | Identify the report as a systematic review. | 1 |
| **ABSTRACT** | | |  |
| Abstract | 2 | See the PRISMA 2020 for Abstracts checklist. | 1 |
| **INTRODUCTION** | | |  |
| Rationale | 3 | Describe the rationale for the review in the context of existing knowledge. | 2 |
| Objectives | 4 | Provide an explicit statement of the objective(s) or question(s) the review addresses. | 2 |
| **METHODS** | | |  |
| Eligibility criteria | 5 | Specify the inclusion and exclusion criteria for the review and how studies were grouped for the syntheses. | 3 |
| Information sources | 6 | Specify all databases, registers, websites, organisations, reference lists and other sources searched or consulted to identify studies. Specify the date when each source was last searched or consulted. | 2,3 |
| Search strategy | 7 | Present the full search strategies for all databases, registers and websites, including any filters and limits used. | Supplementary Table 2 |
| Selection process | 8 | Specify the methods used to decide whether a study met the inclusion criteria of the review, including how many reviewers screened each record and each report retrieved, whether they worked independently, and if applicable, details of automation tools used in the process. | 3,4 |
| Data collection process | 9 | Specify the methods used to collect data from reports, including how many reviewers collected data from each report, whether they worked independently, any processes for obtaining or confirming data from study investigators, and if applicable, details of automation tools used in the process. | 2,3 |
| Data items | 10a | List and define all outcomes for which data were sought. Specify whether all results that were compatible with each outcome domain in each study were sought (e.g. for all measures, time points, analyses), and if not, the methods used to decide which results to collect. | 3,4 |
|  | 10b | List and define all other variables for which data were sought (e.g. participant and intervention characteristics, funding sources). Describe any assumptions made about any missing or unclear information. | 3,4 |
| Study risk of bias assessment | 11 | Specify the methods used to assess risk of bias in the included studies, including details of the tool(s) used, how many reviewers assessed each study and whether they worked independently, and if applicable, details of automation tools used in the process. | 3,4 |
| Effect measures | 12 | Specify for each outcome the effect measure(s) (e.g. risk ratio, mean difference) used in the synthesis or presentation of results. | 4 |
| Synthesis methods | 13a | Describe the processes used to decide which studies were eligible for each synthesis (e.g. tabulating the study intervention characteristics and comparing against the planned groups for each synthesis (item #5)). | 3,4 |
|  | 13b | Describe any methods required to prepare the data for presentation or synthesis, such as handling of missing summary statistics, or data conversions. | 3,4 |
|  | 13c | Describe any methods used to tabulate or visually display results of individual studies and syntheses. | 3,4 |
|  | 13d | Describe any methods used to synthesize results and provide a rationale for the choice(s). If meta-analysis was performed, describe the model(s), method(s) to identify the presence and extent of statistical heterogeneity, and software package(s) used. | 3,4 |
|  | 13e | Describe any methods used to explore possible causes of heterogeneity among study results (e.g. subgroup analysis, meta-regression). | 3,4 |
|  | 13f | Describe any sensitivity analyses conducted to assess robustness of the synthesized results. | 4 |
| Reporting bias assessment | 14 | Describe any methods used to assess risk of bias due to missing results in a synthesis (arising from reporting biases). | 4 |
| Certainty assessment | 15 | Describe any methods used to assess certainty (or confidence) in the body of evidence for an outcome. | 4 |
| **RESULTS** | | |  |
| Study selection | 16a | Describe the results of the search and selection process, from the number of records identified in the search to the number of studies included in the review, ideally using a flow diagram. | 5 |
|  | 16b | Cite studies that might appear to meet the inclusion criteria, but which were excluded, and explain why they were excluded. | Supplementary Table 3 |
| Study characteristics | 17 | Cite each included study and present its characteristics. | Supplementary Table 4 |
| Risk of bias in studies | 18 | Present assessments of risk of bias for each included study. | Supplementary Table 5 |
| Results of individual studies | 19 | For all outcomes, present, for each study: (a) summary statistics for each group (where appropriate) and (b) an effect estimate and its precision (e.g. confidence/credible interval), ideally using structured tables or plots. | 6-14 |
| Results of syntheses | 20a | For each synthesis, briefly summarise the characteristics and risk of bias among contributing studies. | 6-14 |
|  | 20b | Present results of all statistical syntheses conducted. If meta-analysis was done, present for each the summary estimate and its precision (e.g. confidence/credible interval) and measures of statistical heterogeneity. If comparing groups, describe the direction of the effect. | 6-14, Supplementary Figure 1-20 |
|  | 20c | Present results of all investigations of possible causes of heterogeneity among study results. | 16 |
|  | 20d | Present results of all sensitivity analyses conducted to assess the robustness of the synthesized results. | 15 |
| Reporting biases | 21 | Present assessments of risk of bias due to missing results (arising from reporting biases) for each synthesis assessed. | 8-10 |
| Certainty of evidence | 22 | Present assessments of certainty (or confidence) in the body of evidence for each outcome assessed. | 16,17 |
| **DISCUSSION** | | |  |
| Discussion | 23a | Provide a general interpretation of the results in the context of other evidence. | 17 |
|  | 23b | Discuss any limitations of the evidence included in the review. | 17,18 |
|  | 23c | Discuss any limitations of the review processes used. | 19 |
|  | 23d | Discuss implications of the results for practice, policy, and future research. | 17,18,19 |
| **OTHER INFORMATION** | | |  |
| Registration and protocol | 24a | Provide registration information for the review, including register name and registration number, or state that the review was not registered. | 2 |
|  | 24b | Indicate where the review protocol can be accessed, or state that a protocol was not prepared. | 2 |
|  | 24c | Describe and explain any amendments to information provided at registration or in the protocol. | 2 |
| Support | 25 | Describe sources of financial or non-financial support for the review, and the role of the funders or sponsors in the review. | 20 |
| Competing interests | 26 | Declare any competing interests of review authors. | 20 |
| Availability of data, code and other materials | 27 | Report which of the following are publicly available and where they can be found: template data collection forms; data extracted from included studies; data used for all analyses; analytic code; any other materials used in the review. | / |

**Supplementary Table 2. The detailed search strategies**

**PubMed**

| **Number** | **search terms** |
| --- | --- |
| #1 | China [MeSH Terms] |
| #2 | China [Text Word] OR Chinese [Text Word] |
| #3 | #1 OR #2 |
| #4 | hypertension [MeSH Terms] |
| #5 | hypertension [Title/Abstract] OR high blood pressure [Title/Abstract] |
| #6 | #4 OR #5 |
| #7 | cognitive dysfunction [MeSH Terms] |
| #8 | cognition disorder [Title/Abstract] OR cognitive impairment [Title/Abstract] OR cognitive deficit [Title/Abstract] |
| #9 | #7 OR #8 |
| #10 | #3 AND #6 AND #9 |

**Web of Science**

| **Number** | **search terms** |
| --- | --- |
| #1 | TS=(China) |
| #2 | TS=(Chinese) |
| #3 | #1 OR #2 |
| #4 | TS=(hypertension) |
| #5 | TS=(“high blood pressure”) |
| #6 | 6: #4 OR #5 |
| #7 | TS=(“cognitive dysfunction”) |
| #8 | TS=(“cognition disorder”) |
| #9 | TS=(“cognitive impairment”) |
| #10 | TS=(“cognitive deficit”) |
| #11 | #7 OR #8 OR #9 OR #10 |
| #12 | #3 AND #6 AND #11 |

**Embase**

| **Number** | **search terms** |
| --- | --- |
| #1 | 'China' OR 'Chinese' |
| #2 | 'hypertension':ti,ab,kw OR 'high blood pressure':ti,ab,kw |
| #3 | 'cognitive dysfunctio':ab,ti,kw OR 'cognition disorde':ab,ti,kw OR 'cognitive impairment':ab,ti,kw OR 'cognitive deficit':ab,ti,k |
| #4 | #1 AND #2 AND #3 |

**The Cochrane Library**

| **Number** | **search terms** |
| --- | --- |
| #1 | (China OR Chinese):ti,ab,kw |
| #2 | (hypertension OR “high blood pressure”):ti,ab,kw |
| #3 | (“cognitive dysfunction” OR “cognition disorder” OR “cognitive impairment” OR “cognitive deficit”):ti,ab,kw |
| #4 | #4 #1 and #2 and #3 |

**CNKI**

FT = (中国+中国人) AND SU=(高血压) AND SU=(认知障碍+认知功能障碍）

**CBM**

( "中国"[全部字段:智能] OR "中国人"[全部字段:智能]) AND "高血压"[常用字段:智能] AND( "认知障碍"[常用字段:智能] OR "认知功能障碍"[常用字段:智能])

**Wanfang database**

**全部:(中国) and 主题:(高血压) and 主题:(认知障碍 OR 认知功能障碍)**

**VIP任意字段=中国 AND 题名或关键词=高血压 AND 题目或关键词=认知障碍 OR 认知功能**

**障碍**

**Supplementary Table 3. The list of excluded studies**

**Not observational studies (n=4) [1-4]**

1. Cui J, Yu R, Li M, Gao J, Cui Y. Intervention Affects the Cognitive Performance of Middle-Aged Patients with Essential Hypertension. *International Journal of Clinical and Experimental Medicine* (2016) 9(1):308-15.

2. Huang Y, Zheng H, Tan K, Sun X, Ye J, Zhang Y. Circulating Metabolomics Profiling Reveals Novel Pathways Associated with Cognitive Decline in Patients with Hypertension. *Therapeutic Advances in Neurological Disorders* (2020) 13:1-18. doi: 10.1177/1756286420947973.

3. Li X, Ma C, Sun X, Zhang J, Chen Y, Chen K, et al. Disrupted White Matter Structure Underlies Cognitive Deficit in Hypertensive Patients. *European Radiology* (2016) 26(9):2899-907. doi: 10.1007/s00330-015-4116-2.

4. Xiao Y, Chen X, Lin Q, Wu L. Risk Factors for Cognitive Impairment in Hypertensive Patients and Their Prevention. *Chin J Lab Diagn* (2021) 25(2):272-5. Epub 20210910.

**Conference studies (n=2) [5-6]**

5. Zou J, Chen X, Xu Y. Effect of Hypertension on White Matter Microstructure and Its Correlation with Cognitive Function. *Journal of Cerebral Blood Flow and Metabolism* (2019) 39(1):404. doi: 10.1177/0271678X19851020.

6. Zou Y, Zhu QL, Dai R, Deng YT, Duan JX, Pan L, et al. Vascular Risk Factors and Mild Cognitive Impairment in the Elderly Population in Southwest China. *Journal of the American Geriatrics Society* (2013) 61:S343. doi: 10.1111/jgs.12439.

**Unclear time and location of research (n=9) [7-15]**

7. Chen L, Yang LZ, Lu JY, Gao H. The Mild Cognitive Dysfunction and Its Influencing Factors in Community Dwelling Older Patients with Hypertension in Nanning. *Chin J Nurs Educ* (2022) 19(06):566-71. doi: 10.3761/j.issn.1672-9234.2022.06.017.

8. Chen XP, Wu J, Peng Q, Huang H, Mao ZX, Huang Y, et al. Relationship between Microalbuminuria and Cognition in Primary Hypertension Patients. *Chin J Cardio* (2008) 36(8):722-5. Epub 20090130. doi: 10.3321/j.issn:0253-3758.2008.08.012.

9. Chen XY, Yang Y, Tang ZY, Lyu J, Shi FX, Chen YR, et al. Association between Hypertension with Hyperhomocysteinemia and Cognitive Impairment in the Kailuan Community of China: A Cross-Sectional Study. *Biomedical and environmental sciences : BES* (2021) 34(7):557-61. Epub 2021/08/07. doi: 10.3967/bes2021.076.

10. Ciao XL. Study on the Relationship between Insulin Resistance and Cognition Impairment in Middle-Aged Hypertensive Patients. *J Bengbu Med Coll* (2019) 44(2):264-7. Epub 20191110.

11. Lu JY. (2021) Construction of a Risk Prediction Model for Mild Cognitive Impairment in Elderly Hypertensive Patients in the Community [dissertation/master's thesis].[China]: Guangxi University of Traditional Chinese Medicine.

12. Peng Q, Wang XJ, Yu Q, Yang Y, Chen M. Relationship between Blood Pressure Variability and Cognition in Hypertensive Patients. *China Journal of Modern Medicine* (2012) 22(7). Epub 20120726.

13. Tian C. (2015) Analysis on the Assessment of Cognitive Function Status and Evidence Characteristics of Elderly Hypertensive Patients [dissertation/master's thesis].[China]: Beijing University of Chinese medicine.

14. Zhang Y, Li Y, Wang R, Sha G, Jin H, Ma L. Elevated Urinary Ad7c-Ntp Levels in Older Adults with Hypertension and Cognitive Impairment. *Journal of Alzheimer's Disease* (2020) 74(1):237-44. doi: 10.3233/JAD-190944.

15. Zhao YW, Qu Y, Zhao JH, Fang NY, Hu ZP. The Relationship between the Cognitive Impairment of the Elderly Hypertensive with the Adc Values of the Mr-Dwi. *Chin J Clin Neurosci* (2011) 19(3). Epub 20111026.

**Ineligible patients (n=55) [16-70]**

16. Bai H, Yin XM, Tang WJ, Yin LF, Liu FF. Effects of Estradiol, Homocysteine and Circadian Rhythm of Blood Pressure on Cognitive Function in the Postmenopausal Women Paitents with Hypertension. *Chin J Clin Healthc* (2022) 25(1):75-8. Epub 20220921. doi: 10.3969/J.issn.1672-6790.2022.01.017.

17. Bai J, Wei P, Zhao N, Xiao Y, Yang C, Zhong J, et al. A Study of Mild Cognitive Impairment in Veterans: Role of Hypertension and Other Confounding Factors. *Neuropsychology, development, and cognition Section B, Aging, neuropsychology and cognition* (2016) 23(6):703-15. doi: 10.1080/13825585.2016.1161000.

18. Bao J, Liu J, Li Z, Zhang Z, Su X, Sun J, et al. Relationship between Hypertension and Cognitive Function in an Elderly Population: A Population-Based Study in Rural Northern China. *Frontiers in neurology* (2022) 13:885598. Epub 2022/06/03. doi: 10.3389/fneur.2022.885598.

19. Chen B, Jin X, Guo R, Chen Z, Hou X, Gao F, et al. Metabolic Syndrome and Cognitive Performance among Chinese ≥50 Years: A Cross-Sectional Study with 3988 Participants. *Metabolic syndrome and related disorders* (2016) 14(4):222-7. Epub 2016/03/10. doi: 10.1089/met.2015.0094.

20. Chen XF, Sun YJ, Liu HY, Ding YQ, Liu SJ, Chen JH, et al. Related Factors of Cognitive Impairment after Stroke. *Neural Regeneration Research* (2006) 1(8):763-5.

21. Chou C, Chien L, Lin M, Wang C-J. Cognitive Function and Associated Factors among Postmenopausal Women with Hypertension and Natural Menopause in Taiwan. *Geriatric Nursing* (2021) 42(1):110-6. doi: 10.1016/j.gerinurse.2020.12.007.

22. Chuang S, Cheng H, Mitchell GF, Sung S-H, Chen C-H, Pan W-H, et al. Carotid Flow Velocities and Blood Pressures Are Independently Associated with Cognitive Function. *American Journal of Hypertension* (2019) 32(3):289-97. doi: 10.1093/ajh/hpy165.

23. Cui GH, Guo HD, Xu RF, Jiang GX, Chen SD, Cheng Q. The Association of Weight Status with Cognitive Impairment in the Elderly Population of a Shanghai Suburb. *Asia Pacific journal of clinical nutrition* (2013) 22(1):74-82. Epub 2013/01/29. doi: 10.6133/apjcn.2013.22.1.18.

24. Cui QT, Fu QL, Han PL, Zhang J. Risk Factors of Cognitive Impairment after Off-Pump Coronary Artery Bypass Grafting. *Chinese Journal of Cardiology* (2012) 40(2):104-7. doi: 10.3760/cma.j.issn.0253-3758.2012.02.006.

25. Duan JH, Chen YX, Li DX, Li SH. Analysis of Risk Factors for Postoperative Cognitive Dysfunction in Elderly Patients with Hypertension. *Chinese Journal of Practical Nervous Disease* (2014) 17(7):86-7. Epub 20141027.

26. Gao H, Wang K, Ahmadizar F, Zhuang J, Jiang Y, Zhang L, et al. Associations of Changes in Late-Life Blood Pressure with Cognitive Impairment among Older Population in China. *Bmc Geriatrics* (2021) 21(1). doi: 10.1186/s12877-021-02479-1.

27. Gao L. (2013) Effects of Hypertension on Brain Structure and Function in Patients with Alzheimer's Disease [dissertation/master's thesis].[China]: LanZhou University.

28. Gao X, Duan C, Bao L, Yu H, Qin B, Qi R, et al. Effect of Type 2 Diabetes with Hypertension on Cognitive Function-a Study on Elderly Living in the Communities of Beijing. *Zhonghua liu xing bing xue za zhi = Zhonghua liuxingbingxue zazhi* (2014) 35(7):784-6.

29. Gao Z, Zhou JF, Tong L, Q. Relationship between Depression after Hypertension and Mild Cognitive Impairment. *Anhui Medical and Pharmaceutical Journal* (2012) 16(09):1307-8.

30. Ge TA. (2018) Effect of Cerebral Vascular Hemodynamic Index on Mild Cognitive Impairment in Elderly Patients with Hyperteen [dissertation/master's thesis].[China]: Zhejiang University.

31. Gong QY, Shao PL, M. HJ. Relationship of Carotid Intima-Media Thickness and Epicardial Fat Thickness with Mild Cognitive Impairment in Elderly Patients with Masked Hypertension. *Chinese Journal Practice* (2022) 25(33):4139-44. Epub 20230120.

32. Gou MQ, Sun HQ, Yang YR, Zuo GC. Analysis of Risk Factors of Cognitive Dysfunction in Elderly Patients with Hypertensive Stroke. *Geriatr Health Care* (2021) 27(05):982-6.

33. Gu Y, Liu R, Qin R, Chen X, Zou J, Jiang Y, et al. Characteristic Changes in the Default Mode Network in Hypertensive Patients with Cognitive Impairment. *Hypertension Research* (2019) 42(4):530-40. doi: 10.1038/s41440-018-0176-4.

34. Guan BB. (2020) Case-Control Study on the Risk Factors of the Stroke and Hypertension Patients with Memory Dysfunction [dissertation/master's thesis].[China]: North China University of Science and Technology.

35. Guan SC, Tang Z, Wu XG, Diao LJ, Liu HJ, Sun F, et al. Investigation of the Incidence of Mild Cognitive Impairment and Its Risk Factors in an Elderly Population Sample in Beijing Area. *Chinese Journal of Cerebrovascular Diseases* (2008) 5(9):395-8.

36. Guo LJ, Bian QW, Zhang SY. Postural Blood Pressure Changes in Elderly Hypertensive Patients and the Effect on Neurocognitive Impairment. *Chinese Journal of Practical Nervous Disease* (2013) 16(20):73-4. Epub 20140326.

37. Guo Q, Zhang JQ, Shi R. The Relationship between Hypertension Combined with Diabetes Mellitus and Cognitive Function in the Elderly. *Chinese Journal of Multiple Organ Diseases in the Elderly* (2017) 16(1):38-42. doi: 10.11915/j.issn.1671-5403.2017.01.009.

38. Guo XL, Yang B, Zhuang XS, Hu CY, Tang LL, Hao CY. Analysis of Cognitive Function and the Influencing Factors in Elderly Patients with Chronic Heart Failure Complicated with Hypertension. *Chinese Journal of Frontiers in Medicine* (2021) 13(3):90-4. Epub 20210910.

39. He Q. (2017) Risk Factors of Cognitive Impairment in Elderly Patients of Hypertension with Atrial Fibrillation [dissertation/master's thesis].[China]: Lanzhou University.

40. Kou YF, Zhao HJ, Wang QY. Relationship between Serum Hcy Level and Cognitive Impairment in Patients with Hypertensive Basal Ganglia Hemorrhage Complicated with Depression. *PJCCPVD* (2020) 28(11):54-8. Epub 20210603.

41. Li D, Wang ZX. Vascular Cognitive Function in Patients with Hypertensive Cerebral Hemorrhage Factors Influencing the Occurrence of Impairment. *Chin J of Public Health Eng* (2021) 20(2):227-8,30. Epub 20210910.

42. Li H, Sun D, Lu D, Zhang J, Zeng J. Low Hippocampal Dentate Gyrus Volume Associated with Hypertension-Related Cognitive Impairment. *American Journal of Alzheimer's Disease and other Dementias* (2020) 35. doi: 10.1177/1533317520949782.

43. Li J, Yu S, Tan Z, Yu Y, Luo L, Zhou W, et al. High Estimated Glomerular Filtration Rate Is Associated with Worse Cognitive Performance in the Hypertensive Population: Results from the China H-Type Hypertension Registry Study. *Frontiers in aging neuroscience* (2021) 13:706928. Epub 2022/03/08. doi: 10.3389/fnagi.2021.706928.

44. Lin Y. Ambulatory Blood Pressure and Left Ventricular Hypertrophy and Cognitive Status of Correlation. *Chin J Postgrad Med* (2010) (22). Epub 20141222.

45. Liu L, Li J, Wang CX, Wang YF, Guo JF, Guo LH. Factors Influencing the Development of Cognitive Dysfunction in Patients with Primary Hypertension Combined with Lacunar Cerebral Infarction. *Chinese Physicians Journal* (2022) 24(6):931-3. Epub 20220921.

46. Lu S, Shao L, Zhang Y, Yang Y, Wang Z, Zhang B, et al. Predictive Value of Gut Microbiome for Cognitive Impairment in Patients with Hypertension. *Disease Markers* (2021) 2021. doi: 10.1155/2021/1683981.

47. Lu S, Xu Q, Yu J, Yang Y, Wang Z, Zhang B, et al. Prevalence and Possible Factors of Cognitive Frailty in the Elderly with Hypertension and Diabetes. *Frontiers in Cardiovascular Medicine* (2022) 9. doi: 10.3389/fcvm.2022.1054208.

48. Ma CL. Risk Factors of Early Vascular Cognitive Impairment in Patients with Hypertensive Cerebral Hemorrhage. *Chinese Journal of Practical Nervous Disease* (2013) 16(19):16-8. Epub 20140326.

49. Ma L, Feng M, Lan Y, Yang W, Liu J, Han R, et al. Insulin Resistance Is an Important Risk Factor for Cognitive Impairment in Elderly Patients with Primary Hypertension. *Yonsei Medical Journal* (2015) 56(1):89-94. doi: 10.3349/ymj.2015.56.1.89.

50. Ma Y, Hua R, Yang Z, Zhong B, Yan L, Xie W. Different Hypertension Thresholds and Cognitive Decline: A Pooled Analysis of Three Ageing Cohorts. *BMC medicine* (2021) 19(1):287. Epub 2021/11/03. doi: 10.1186/s12916-021-02165-4.

51. Qin H, Zhu B, Hu C, Zhao X. Later-Onset Hypertension Is Associated with Higher Risk of Dementia in Mild Cognitive Impairment. *Frontiers in Neurology* (2020) 11. doi: 10.3389/fneur.2020.557977.

52. S G, Y J, FW U, C L, KS H, F M, et al. Hypertension and Cognitive Decline in Rural Elderly Chinese. *Journal of the American Geriatrics Society* (2009) 57(6):1051-7.

53. Shen YQ, Ma L, Ji CY. Characteristics of Cardiac Structural Function and Vascular Function in Perimenopausal Hypertensive Patients and Correlation with Cognitive Function. *Maternal and Child Health Care of China* (2020) 35(24). Epub 20210629.

54. Wang C, Zhao JF, Feng LL, Zhang ZP, Liu QR, Yu HL, et al. Analysis of Cognitive Function in Hypertension Patients. *Chin J Prev Contr Chron Dis* (2016) (4). Epub 20161130.

55. Wang H, Liu T, Cai YY, Jiang H, Liu HX, Lin C. Kidney Function and Cognitive Impairment in People Aged 80 Years and over with Untreated Hypertension: A Cross-Sectional Survey. *Kidney & blood pressure research* (2016) 41(1):70-7. Epub 2016/02/13. doi: 10.1159/000368550.

56. Wang TT. (2017) Research on the Prevalence and Community Management Strategies of Mild Cognitive Impairment among the Elderly in Three District of Chongqing [dissertation/master's thesis].[China]: Chongqing Medical University.

57. Wang W, Fang J, Lei B. Blood Pressure Levels and Prognosis of Intracranial Trauma Patients with Cognitive Dysfunction. *Pakistan Journal of Medical Sciences* (2014) 30(4). doi: 10.12669/pjms.304.4930.

58. Wu YJ, Wang LN. Study on the Cognitive Function and R Elated Factors of the Elders over 60. *Chin J Contemp Neurol Neurosurg* (2007) 7(2). Epub 20131012.

59. Xue CJ, Dong B, Zhao YN, Li JR, Zhang Y. The Relationship between Changes of Plasma Endothelin －1 and Homocysteine with Cognitive Dysfunction in Patients of Hypertensive Cerebral Hemorrhage Combined Obstructive Sleep Apnea Hypopnea. *Modern Preventive Medicine* (2016) 43(06):1138-41.

60. Zeng BM, Guo GL. Characteristics of Cognitive Impairment in Hypertension Combined with Lacunar Cerebral Infarction and Chinese Medical Evidence Analysis. *Chin J Mod Drug Appl* (2018) 12(21). Epub 20190520.

61. Zhang B, Guo Q, Jia XX. Characteristics of Cognitive Impairment in Hypertension Combined with Lacunar Cerebral Infarction and Analysis of Factors Affecting It. *Neural Injury And Functional Reconstruction* (2020) 15(12):737-9+44. doi: 10.16780/j.cnki.sjssgncj.20181346.

62. Zhang HM, Li J. Relationship between Serum Uric Acid Levels and Cognitive Impairment in Patients with Vascular Dementia and Type H Hypertension. *China Geriatric Health Care Medicine* (2019) 17(05):25-8.

63. Zhang J. Correlation between Cognitive Function and Prethrombotic State in Elderly Patients with Hypertension. *Chinese Journal of Thrombosis and Hemostasis* (2021) 27(01):50-2.

64. Zhang J, Liu L, Sun H, Li M, Li Y, Zhao J, et al. Cerebral Microbleeds Are Associated with Mild Cognitive Impairment in Patients with Hypertension. *Journal of the American Heart Association* (2018) 7(11). Epub 2018/06/03. doi: 10.1161/jaha.117.008453.

65. Zhang X, He JK, Zhao Q. Analysis of Cognitive Impairment and Risk Factors in Patients with Obstructive Sleep Apnea Syndrome Complicated with Hypertension. *Chinese Journal of Frontiers in Medicine* (2016) 8(12). Epub 20170605.

66. Zhang YX, Yu JL. Investigation Cognitive Impairment and Related Factors of Aged People in Elderly Care Institutions of Chongqing. *J Mod Med Health* (2016) 32(16). Epub 20170128.

67. Zhao HG, Liang YL, Qi LS, Xu BX. Effect of Apoe Gene Polymorphism and Serum Cyclophilin a on Cognitive Function in Patients with Hypertensive Intracerebral Hemorrhage. *Chinese Journal of Practical Nervous Diseases* (2021) 24(4). Epub 20210910.

68. Zhou L, Lai YW, Jiang C, Wang J, Wang ZY, Dai WL, et al. Association of Hypertension and Blood Pressure Levels with Mild Cognitive Impairment in Patients with Atrial Fibrillation. *Journal of Cardiovascular & Pulmonary Diseases* (2022) 41(07):743-8.

69. Zhu Y. (2021) Blood Pressure Parameters in Patients with Maintenance Peritoneal Dialysis Complicated with Hypertension Cognitive Function [dissertation/master's thesis].[China]: Nanjing Medical University.

70. Zong YH, Shi XH. The Effect of Hypertension on Cognitive Function in the Elderly in China. *J Prev Med Inf* (2021) 37(02):243-8.

**No related outcomes (n=32) [71-102]**

71. Cao JJ, Hou R, Zhao C, Zhao DX, Wang YW, Jiang HS, et al. Evaluation of Ankle-Brachial Index on Cognitive Dysfunction in Elderly Patients with Essential Hypertension. *Chinese Maternal and Child Health Study* (2017) (S4). Epub 20190520.

72. Cao JJ, Hou R, Zhao DX, Wang YW, Zhao C, Zhang KY, et al. Relationship between Ankle-Brachial Index, Pulse Pressure Index and Cognitive Function in Elderly Patients with Essential Hypertension. *Chinese Maternal and Child Health Study* (2017) (S4). Epub 20190520.

73. Ding LL, Zhu XH, Xiong ZF, Yang F, Zhang XN. The Association of Age at Diagnosis of Hypertension with Cognitive Decline: The China Health and Retirement Longitudinal Study (Charls). *Journal of General Internal Medicine* (2022). doi: 10.1007/s11606-022-07951-1.

74. Du BZ. Relation between Serum Lipid and Cognitive Function in Elderly Patients with Essential Hypertension. *CHINA HEALTH STANDARD MANAGEMENT* (2018) 9(6). Epub 20191110.

75. Du WW, Jia X, F., Su C, Wang ZH, Wang HJ, Zhang CX, et al. Blood Pressure Status of People Aged ≥55 Years in Four Chinese Provinces Association with Mild Cognitive Impairment. *Chin J Hyperten* (2021) 29(11). Epub 20230120.

76. Du WW, Jia X, F., Su C, Wang ZH, Wang HJ, Zhang CX, et al. Blood Pressure Status of People Aged ≥55 Years in Four Chinese Provinces Association with Mild Cognitive Impairment. *Journal of Environmental and Occupational* (2021) 38(8). Epub 20220523.

77. Guo H. (2022) A Longitudinal Study on the Association between Blood Pressure and Mild Cognitive Impairment in Adults in Rural Areas of Liaoning Province [dissertation/master's thesis].[China]: Chinese Medical University.

78. Guo HF, Pan FF, Geng H, Sun XK, Zhong Y. Correlation of Mean Arterial Pressure with Cognitive Decline in the Elderly. *Chinese Journal of Gerontology* (2019) (3). Epub 20200430.

79. He HB, Yang CM, Duan XW, Fan JH. Study on Relationship between Cognitive Impairment and Dynamic Blood Pressure in the Elderly Patients with Hypertension. *Chinese Journal of Misdiagnostics* (2002) 2(3). Epub 20021231.

80. He HB, Zhou HD, Chen ME, Li JC, Zhang M, Wang YJ, et al. Relationship between Blood Pressure and Cognitive Function of Aged People in Chongqing Communities: Stratified Cluster Sampling. *Chinese Journal of Clinical Rehabilitation* (2004) 8(24). Epub 20050130.

81. Huang F, Yuan Y, Shi W, Zhu P. The Association between Anticardiolipin Antibodies and Cognitive Function in Hypertensive Patients. *Journal of the American Geriatrics Society* (2019) 67:S88-S9. doi: 10.1111/jgs.15898.

82. Huang WY, Zhang N, Yang JY, Yang X, Deng HC, Lei MY. Effect of Hypertension on Cognitive Function among the Elderly in a Community of Guiyang City. *Chin J Geriatr Heart Brain Vessel Dis* (2008) (01):7-10.

83. Jiang C, Li S, Wang Y, Lai Y, Bai Y, Zhao M, et al. Diastolic Blood Pressure and Intensive Blood Pressure Control on Cognitive Outcomes: Insights from the Sprint Mind Trial. *Hypertension (Dallas, Tex : 1979)* (2023) 80(3):580-9. doi: 10.1161/HYPERTENSIONAHA.122.20112.

84. Jiang HS. (2012) The Relationship between Ankle-Brachial Index, Pulse Pressure Index and Cognitive Function in the Elderly Patients with Essential Hypertension [dissertation/master's thesis].[China]: Chengde Medical University.

85. Li J, Xu GH, Liu XS, Shen QY, Wang K, Li YH. Study of the Effects of Stage I Hypertension on Cognitive Function in the Elderly. *Acta Universitatis Medicinalis Anhui* (2015) 50(03):349-52. doi: 10.19405/j.cnki.issn1000-1492.2015.03.021.

86. Liao CY. (2022) Study on the Relationship between Physical Activity, Cardiorespiratory Fitness and Cognitive Function in the Elderly with Hypertension [dissertation/master's thesis].[China]: Shanghai Institute of Sports.

87. Liu YH, Liu XD. Application Effect of Minimental State Examination in Elderly Hypertensive Patients in Community. *J Community Med* (2019) 17(15):900-3. doi: 10.19790/j.cnki.JCM.2019.15.05.

88. Qian JH, Ren XH. Association between Comorbid Conditions and Badl/Iadl Disability in Hypertension Patients over Age 45 Based on the China Health and Retirement Longitudinal Study (Charls). *Medicine* (2016) 95(31). doi: 10.1097/md.0000000000004536.

89. Sun HT, Wang C. Different Manifestations of Cognitive Dysfunction in Hypertensive Patients with Different Syndromes of Traditional Chinese Medicine. *Chinese Journal of Clinical Rehabilitation* (2005) 9(20):63-5.

90. Sun PX, Yan ZR, Yue HM, Meng QY. Analysis on the Correlation between Hypertension and Geriatric Cognitive Impairment. *J Jining Med Univ* (2013) 36(01):43-5.

91. Wang B, Qiao YS, Li YJ, Wang JJ. Correlation Analysis of Hippocampal Volume and Mild Cognitive Impairment in Patients with Hypertension *Chinese Remedies & Clinics* (2016) 16(7). Epub 20170323.

92. Wang L, Chen J, Li J, Hu F, Xie Y, Zhou X, et al. Threshold Effect of Plasma Total Homocysteine Levels on Cognitive Function among Hypertensive Patients in China: A Cross-Sectional Study. *Frontiers in neurology* (2022) 13:890499. Epub 2022/09/06. doi: 10.3389/fneur.2022.890499.

93. Wang TT, Cao C, Deng J, Lian JX, Yan K, Wang ZZ, et al. Prevalence and Risk Factors of Mild Cognitive Impairment among Old People in Chongqing, China. *Chin J Rehabil Theory Pract* (2017) 23(7). Epub 20171231.

94. Wang XN, Bai XJ, Qi GX, Wang CL, Shan HY. Relationship between H-Type Hypertension and Cognitive Impairment in Elderly Patients. *Chinese Journal of Arteriosclerosis* (2013) 21(10):894-8.

95. Wang YN, Tang ZH, Chen JL. Relationships of Hypertension and Diabetes with Cognitive Function among Community-Dwelling Middle-Aged and Elderly Population. *Chinese General Practice* (2020) 23(5). Epub 20200804.

96. Xie Y, Li J, Yu G, Zhou X, Zhou W, Zhu L, et al. Association between Lipid Accumulation Product and Cognitive Function in Hypertensive Patients with Normal Weight: Insight from the China H-Type Hypertension Registry Study. *Frontiers in neurology* (2021) 12:732757. Epub 2022/02/22. doi: 10.3389/fneur.2021.732757.

97. Xu JH. Relationship between Visit-to-Visit Blood Pressure Variability and Mild Cognition Impairment. *Chronic Pathematology J* (2019) 20(10):1470-3+6. doi: 10.16440/j.cnki.1674-8166.20191029.001.

98. Yang SQ, Zhang W, Liu JL. Changes of Cognitive Function in Elderly Patients with Essential Hypertension. *Clin J Med Offic* (2018) 46(7). Epub 20190520.

99. Yi ZG, Sun Q, Zhang HM, Yang MY, Zhao MD. Effect of Hypertension on Cognitive Function in People over 65 *Contemporary Medicine* (2013) 19(04):40-1.

100. Yuan JQ, Lv YB, Chen HS, Gao X, Yin ZX, Wang WT, et al. Association between Late-Life Blood Pressure and the Incidence of Cognitive Impairment: A Community-Based Prospective Cohort Study. *Journal of the American Medical Directors Association* (2019) 20(2):177-82.e2. Epub 2018/07/19. doi: 10.1016/j.jamda.2018.05.029.

101. Zhong J. Analysis of Community Hypertension Control Effects and Influencing Factors. *Mod Diagn Treat* (2017) 28(04):701-2.

102. Zhu YP, Chen MF, Shen BH. A Prevalencestudy on Mild Cognitive Impairment among Elderly Populationsin Zhejiang Province. *Chin J Epidemiol* (2013) 34(5). Epub 20130917.

**Unavailable data (n=3) [103-105]**

103. Liao D, Guo ZP, Tang LR, Gao Y, Zhang ZQ, Yang MH, et al. Alterations in Regional Homogeneity and Functional Connectivity Associated with Cognitive Impairment in Patients with Hypertension: A Resting-State Functional Magnetic Resonance Imaging Study. *Hypertension Research* (2023). doi: 10.1038/s41440-023-01168-3.

104. Liao D, Zhang ZQ, Guo ZP, Tang LR, Yang MH, Wang RP, et al. Disrupted Topological Organization of Functional Brain Networks Is Associated with Cognitive Impairment in Hypertension Patients: A Resting-State Fmri Study. *Neuroradiology* (2023) 65(2):323-36. doi: 10.1007/s00234-022-03061-1.

105. Wang WH, Zhao D, Liu S. A Cross-Sectional Study on Cognitive Function and Influencing Factors in Patients with Hypertension. *Chin J Epidemiol* (2007) 28(06).

**Overlapping publication (n=10) [106-115]**

106. Chen XP, Wu J, Peng Q, Huang H, Mao ZX, Huang Y, et al. Relationship between Microalbuminuria and Cognition in Primary Hypertension Patients. *Zhonghua xin xue guan bing za zhi [Chinese journal of cardiovascular diseases]* (2008) 36(8):722-5. doi: 10.3321/j.issn:0253-3758.2008.08.012.

107. He ZL, Yin S, Wu XC, Hu SL. Influencing Factors of Cognitive Dysfunction in Elderly Patients with Hypertension. *Clinical Research and Practice* (2020) 5(21):16-7+23. doi: 10.19347/j.cnki.2096-1413.202021006.

108. Heizhati M, Wang L, Li N, Li M, Pan F, Yang Z, et al. Prevalence of Mild Cognitive Impairment Is Higher in Hypertensive Population: A Cross-Sectional Study in Less Developed Northwest China. *Medicine (Baltimore)* (2020) 99(19):e19891. Epub 2020/05/10. doi: 10.1097/md.0000000000019891.

109. Li H, Zhao C, Lin ZJ, Wang L. Clinical Characteristics of Cognitive Dysfunction in Elderly Patients with Essential Hypertension and Analysis of Their Risk Factors. *Chinese Journal of Integrative Medicine on Cardio-/Cerebrovascular Disease* (2022) 20(03):565-9.

110. Li T, Bai J, Xiang JW, Wang R, Tuo XP, Zhao ZX. Risk Factors of Cognitive Impairment in Elderly Patients with Hypertension: A Cross-Sectional Study in Chinese Elderly. *Journal of the American Geriatrics Society* (2014) 62:S370. doi: 10.1111/jgs.13075.

111. Liu H, Xu XJ. The Analysis of Risk Factors of Primary Hypertensive and Lacunar Infarction Patients with Cognitive Impairment. *Journal of Hypertension* (2018) 36:e181.

112. Liu JL, Chen JH, Yang SQ, Liu XH, Hu XJ, Wang LH, et al. Changes of Cognitive Function in Elderly Patients with Essential Hypertension. *Chinese Journal of Clinical Rehabilitation* (2004) 8(12):2234-6.

113. Peng Y, Luo XJ, Chen XP, Li LX, Wan LY, He S, et al. Association of Pparγ2pro12ala Polymorphism and Cognitive Dysfunction in Hypertension Patients. *J Sichuan Univ (Med Sci Edi)* (2010) 41(06):1034-8. doi: 10.13464/j.scuxbyxb.2010.06.011.

114. Zhang J, Tang G, Xie H, Wang B, He M, Fu J, et al. Higher Adiposity Is Associated with Slower Cognitive Decline in Hypertensive Patients: Secondary Analysis of the China Stroke Primary Prevention Trial. *Journal of the American Heart Association* (2017) 6(10). doi: 10.1161/JAHA.117.005561.

115. Zou MJ, Huang JW, Zeng Y, Cheng GR, Chen C, Li L, et al. Association between Systolic Blood Pressure Control and Cognitive Function in Community-Living Older Adults 65 Years and Older with Hypertension. *Chin J Dis Control Prev* (2021) 25(11):1282-6+92. doi: 10.16462/j.cnki.zhjbkz.2021.11.008.

**Supplementary Table 4. The basic characteristics of the included studies**

| Study | Period | Region/City | Source | Design | Sample size | Gender(M/F) | Mean age | Assessment tools for cognitive impairment | Number of patients with cognitive impairment | Prevalence |
| --- | --- | --- | --- | --- | --- | --- | --- | --- | --- | --- |
| Cai 2018 | 2015.10-2016.10 | North China/Hebei | Hospital-based | Cross-sectional | 151 | 101/50 | 67.6±5.2 | MoCA | 41 | 27.15% |
| Chen 2022 | 2018.01-2021.01 | East China/Zhejiang | Hospital-based | Cross-sectional | 97 | 53/44 | 71.15±8.76 | MMSE | 6 | 6.19% |
| Cheng 2014 | 2011.01-2013.06 | Northwest China/Shaanxi | Hospital-based | Cross-sectional | 329 | 185/144 | 71.87±8.42 | MMSE | 46 | 13.98% |
| Cheng 2011 | 2009.09-2010.12 | Northeast China/Liaoning | Hospital-based | Cross-sectional | 220 | 108/112 | / | MoCA | 123 | 55.91% |
| Chi 2018 | 2014-2015 | Northwest China/Shaanxi | Hospital-based | Cross-sectional | 404 | 200/204 | 63.0±7.8 | MMSE | 81 | 20.05% |
| Feng 2018 | 2016.01-2017.07 | North China/Beijing | Hospital-based | Cross-sectional | 87 | 36/51 | / | MMSE, MoCA, Peterson | 20 | 22.99% |
| Gan 2021 | 2019-2020 | Central China/Hubei | Community-based | Cohort | 1133 | 557/576 | 71.37±5.48 | MoCA | 216 | 19.06% |
| Gao 2020 | 2015.06-2018.06 | Northwest China/Shaanxi | Hospital-based | Cross-sectional | 200 | 130/70 | 74.3±9.2 | MMSE | 120 | 60% |
| Ge 2009 | 2004.07-2004.10 | North China/Beijing | Community-based | Cohort | 589 | / | / | MMSE, Peterson | 107 | 18.17% |
| Geng 2018 | 2013.06-2017.06 | East China/Shanghai | Hospital-based | Cross-sectional | 113 | 63/50 | 74.5±5.0 | MMSE | 28 | 24.78% |
| Gong 2017 | 2014.09-2016.11 | Northeast China/Liaoning | Hospital-based | Cross-sectional | 233 | 104/129 | ≥65 | MMSE | 135 | 57.9% |
| Gu 2019 | 2017.01-2017.09 | East China/Jiangsu | Hospital-based | Cross-sectional | 55 | 28/27 | 50-75 | MoCA, MMSE | 25 | 45.45% |
| He 2020 | 2017.01-2019.01 | Southwest China/Chongqing | Hospital-based | Cross-sectional | 202 | 104/98 | 36-76 | MMSE | 102 | 50.50% |
| He 2020 | 2019.01-2019.10 | East China/Anhui | Hospital-based | Cross-sectional | 150 | 89/61 | 83.24±7.67 | MMSE | 64 | 42.67% |
| Heizhati 2021 | 2019 | Northwest China/Xinjiang | Community-based | Cross-sectional | 11270 | 5922/5348 | 55.19±12.25 | MMSE | 2732 | 24.24% |
| Hu 2013 | 2010.01-2010.10 | North China/Hebei | Community-based | Cohort | 1110 | / | ≥60 | MMSE | 198 | 17.84% |
| Huang 2009 | 2005.04 | Southwest China/Sichuan | / | Cross-sectional | 446 | 139/307 | 93.6±3.41 | MMSE | 265 | 59.42% |
| Jin 2018 | 2015.03-2017.03 | East China/Zhejiang | Hospital-based | Cross-sectional | 90 | 48/42 | 69.5±5.6 | MMSE | 30 | 33.33% |
| Jin 2010 | 2009.01-2009.11 | North China/Hebei | Hospital-based | Cross-sectional | 197 | 98/99 | 68.08±6.69 | MMSE | 38 | 19.29% |
| Ke 2018 | 2010.06-2017.06 | East China/Fujian | Community-based | Cross-sectional | 229 | / | ≥60 | MMSE | 119 | 51.97% |
| Li 2020 | 2018.07-2019.08 | East China/Zhejiang | Hospital-based | Cross-sectional | 146 | 66/80 | ≥65 | MMSE | 60 | 41.10% |
| Li 2021 | 2019.08-2010.08 | North China/Shanxi | Hospital-based | Cross-sectional | 184 | 78/106 | ≥60 | MoCA, MMSE | 90 | 48.91% |
| Li 2016 | 2014.06-2015.06 | North China/Hebei | Hospital-based | Cross-sectional | 330 | 330/0 | ≥60 | MMSE | 43 | 13.03% |
| Li 2017 | 2014.12-2016.03 | East China/Jiangsu | Hospital-based | Cross-sectional | 127 | 84/43 | 73.4±7.12 | MoCA | 95 | 74.80% |
| Li 2021 | 2019.01-2020.09 | Central China/Henan | Hospital-based | Cross-sectional | 138 | 71/67 | / | MoCA, MMSE | 73 | 52.90% |
| Li 2014 | 2010.06-2012.03 | East China/Shanghai | Community-based | Cross-sectional | 1386 | 1180/206 | / | MMSE, CDT | 628 | 45.31% |
| Li 2020 | 2017.10-2019.12 | Southwest China/Sichuan | Hospital-based | Cross-sectional | 300 | 145/155 | 78.3±5.1 | MMSE | 53 | 17.67% |
| Li 2019 | 2015.09-2017.03 | Central China/Henan | Hospital-based | Cross-sectional | 236 | 116/120 | 63±8.8 | MMSE | 116 | 49.15% |
| Li 2022 | 2014.06-2016.10 | North China/Beijing | Community-based | Cohort | 895 | 282/613 | ≥60 | MMSE | 88 | 9.83% |
| Liang 2021 | 2020.08-2020.09 | East China/Shanghai | Community-based | Cohort | 565 | 238/327 | ≥65 | AD-8 | 103 | 18.23% |
| Lin 2015 | 2014.04-2015.02 | East China/Fujian | Hospital-based | Cross-sectional | 346 | 234/112 | 67.8±8.9 | MMSE, MoCA | 189 | 54.62% |
| Lin 2022 | 2019.01-2021.10 | East China/Fujian | Hospital-based | Cross-sectional | 112 | 66/46 | ≥65 | MMSE | 58 | 51.79% |
| Liu 2021 | 2016.01-2019.12 | North China/Beijing | Hospital-based | Cross-sectional | 234 | 113/121 | ≥60 | MoCA | 88 | 37.61% |
| Liu 2018 | 2016.10-2017.06 | Northwest China/Xinjiang | Hospital-based | Cross-sectional | 106 | 52/54 | 61.4±9.01 | MMSE | 30 | 28.30% |
| Liu 2014 | 2011.01-2013.05 | Northeast China/Heilongjiang | Hospital-based | Cross-sectional | 157 | 82/75 | 58.24±6.36 | MoCA | 41 | 26.11% |
| Luo 2022 | 2018.07-2018.08 | Nationwide/nationwide | / | Cohort | 6413 | 3052/3361 | 68.97±6.36 | MMSE | 2434 | 37.95% |
| Luo 2015 | 2011.01-2013.12 | South China/Guangdong | Hospital-based | Cross-sectional | 326 | 180/146 | 66.2±6.43 | MMSE | 68 | 20.86% |
| Ma 2020 | 2018.12-2019.09 | Northwest China/Qinghai | / | Cross-sectional | 162 | 70/92 | 68.3±6.6 | Peterson, CDR | 93 | 57.41% |
| Ma 2021 | 2017.07-2019.12 | East China/Jiangsu | Hospital-based | Cross-sectional | 161 | 80/81 | / | MoCA, MMSE | 89 | 55.28% |
| Ma 2022 | 2019.04-2019.12 | North China/Tianjin | Community-based | Cross-sectional | 4819 | 2009/2810 | 75.1±7.2 | MMSE | 953 | 19.78% |
| Ma 2022 | 2011.09-2011.11 | Northwest China/Ningxia | Community-based | Cross-sectional | 509 | 198/311 | 66.3±6.4 | MMSE | 97 | 19.06% |
| Ma 2015 | 2014.03-2014.08 | Southwest China/Sichuan | Hospital-based | Cross-sectional | 115 | 72/43 | 75.42±6.14 | MMSE | 51 | 44.35% |
| Pan 2021 | 2019.09-2019.12 | East China/Shanghai | Community-based | Cross-sectional | 286 | 137/149 | 75.11±4.86 | MoCA | 168 | 58.74% |
| Peng 2010 | 2018.01-2019.02 | Southwest China/Sichuan | Hospital-based | Cross-sectional | 502 | 254/248 | / | MMSE | 126 | 25.10% |
| Qi 2015 | 2010.06-2014.09 | Northeast China/Liaoning | Hospital-based | Cross-sectional | 258 | 137/121 | 65.45±10.61 | MMSE | 194 | 75.19% |
| Qin 2022 | 2019.10-2021.06 | Central China/Hubei | Hospital-based | Cross-sectional | 60 | 36/24 | 61 | MoCA | 35 | 58.33% |
| Qu 2022 | 2019.12-2020.06 | East China/Shandong | Hospital-based | Cohort | 63 | 32/31 | / | MoCA | 31 | 49.21% |
| Quan 2022 | 2019.01-2020.10 | Northwest China/Shaanxi | Hospital-based | Cross-sectional | 178 | 96/82 | 67.5±7.1 | MMSE, MoCA | 61 | 34.27% |
| Shi 2021 | 2019.08-2020.02 | North China/Hebei | Hospital-based | Cross-sectional | 172 | 72/100 | 57.48 | MMSE, MoCA | 94 | 54.65% |
| Song 2014 | 2010.10-2010.12 | Southwest China/Chongqing | / | Cohort | 134 | / | ≥60 | MMSE | 83 | 61.94% |
| Song 2020 | 2017.01-2018.02 | North China/Hebei | Hospital-based | Cross-sectional | 113 | 77/36 | 62.68±12.43 | MMSE, MoCA | 60 | 53.10% |
| Su 2009 | 2005.12-2006.02 | Southwest China/Sichuan | Hospital-based | Cross-sectional | 200 | 98/102 | 33-82 | MMSE | 42 | 21% |
| Sun 2021 | 2018.10-2020.06 | North China/Inner Mongolia | Hospital-based | Cross-sectional | 189 | 99/90 | 55.62±9.74 | MMSE, MoCA | 72 | 38.10% |
| Wei 2021 | 2007.03-2008.11 | North China/Tianjin | Community-based | Cohort | 97 | 34/63 | / | MMSE, BCAT | 17 | 17.53% |
| Wu 2016 | 2009.09-2010.06 | North China/Beijing | Community-based | Cohort | 1408 | 540/868 | / | MMSE | 233 | 16.55% |
| Wumaer 2015 | 2013.11-2014.08 | Northwest China/Xinjiang | Hospital-based | Cross-sectional | 104 | 54/50 | ≥60 | MMSE | 40 | 38.46% |
| Xi 2022 | 2018.01-2019.01 | Northwest China/Shaanxi | Hospital-based | Cross-sectional | 209 | 119/90 | ≥60 | MMSE | 99 | 47.37% |
| Xu 2020 | 2014.01-2016.12 | East China/Shanghai | Hospital-based | Cross-sectional | 197 | 101/96 | 72.73±6.24 | MMSE, MoCA | 140 | 71.07% |
| Xu 2018 | 2013.01-2016.10 | Southwest China/Sichuan | Hospital-based | Cross-sectional | 164 | 96/68 | 66.8±10.5 | MMSE, MoCA | 69 | 42.07% |
| Xue 2018 | 2017.03-2017.12 | North China/Beijing | Hospital-based | Cross-sectional | 177 | 122/55 | 80-91 | MoCA | 114 | 64.41% |
| Xue 2020 | 2019.01-2019.12 | Northeast China/Liaoning | Hospital-based | Cross-sectional | 100 | 34/66 | / | MMSE, MoCA | 64 | 64% |
| Xue 2019 | 2017.06-2018.06 | North China/Tianjin | Hospital-based | Cross-sectional | 182 | 122/60 | 68.1±8.5 | MoCA | 116 | 63.74% |
| Yan 2022 | 2017.05-2020.10 | East China/Jiangsu | Hospital-based | Cohort | 572 | 307/265 | 61.31±4.74 | MoCA | 256 | 44.76% |
| Yang 2018 | 2017.01-2018.07 | East China/Shanghai | Hospital-based | Cross-sectional | 198 | 137/61 | 83.25±2.51 | MMSE | 105 | 53.03% |
| Yang 2022 | 2020-2021 | North China/Beijing | Community-based | Cohort | 664 | / | ≥65 | MoCA | 424 | 63.86% |
| Ye 2023 | 2021.05-2021.12 | Northeast China/Heilongjiang | Hospital-based | Cross-sectional | 213 | 113/100 | 49.2±11.3 | MoCA | 80 | 37.56% |
| Yuan 2021 | 2010.01-2011.09 | East China/Shanghai | Community-based | Cohort | 2182 | 1047/1135 | 72.9±7.9 | CDR | 139 | 6.37% |
| Zhang 2014 | 2013.01-2013.07 | North China/Hebei | Hospital-based | Cross-sectional | 417 | 254/163 | ≥60 | MMSE | 97 | 23.26% |
| Zhang 2008 | 2007 | nationwide | Hospital-based | Cross-sectional | 525 | / | / | MMSE | 203 | 38.67% |
| Zhang 2008 | 2006.11-2007.02 | North China/Hebei | Hospital-based | Cross-sectional | 194 | / | / | MMSE | 66 | 34.02% |
| Zhang 2011 | 2009.09-2010.12 | Northeast China/Liaoning | Hospital-based | Cross-sectional | 190 | 96/94 | 62.14±11.35 | MoCA | 108 | 56.84% |
| Zhang 2020 | 2018.11-2019.04 | Northwest China/Qinghai | / | Cross-sectional | 240 | 122/118 | 64.11±8.34 | MMSE, MoCA | 52 | 21.67% |
| Zhang 2022 | 2020.08-2020.12 | Central China/Hubei | Community-based | Cohort | 770 | 361/409 | 71.6±5.3 | MMSE | 303 | 39.35% |
| Zhang 2021 | 2019.03-2019.08 | Central China/Hubei | Community-based | Cohort | 639 | / | ≥65 | MMSE | 114 | 17.84% |
| Zhao 2020 | 2017.10-2019.03 | Northwest China/Shaanxi | Hospital-based | Cross-sectional | 320 | 178/142 | 72.5±4.2 | MMSE | 64 | 20% |
| Zhao 2016 | 2011.01-2015.03 | Central China/Henan | Hospital-based | Cross-sectional | 502 | / | / | MMSE | 197 | 39.24% |
| Zhao 2014 | 2009.04-2011.02 | East China/Shanghai | Hospital-based | Cross-sectional | 171 | 99/72 | 60-79 | MoCA, CDR | 138 | 80.7% |
| Zheng 2021 | 2019.04-2021.04 | Northwest China/Gansu | Hospital-based | Cross-sectional | 110 | 63/47 | / | MMSE, MoCA | 62 | 56.36% |
| Zhou 2018 | 2015.01-2017.05 | Central China/Hubei | Hospital-based | Cross-sectional | 260 | 138/122 | 33-69 | MMSE | 68 | 26.15% |
| Zhou 2011 | 2009.11-2010.12 | North China/Beijing | Community-based | Cross-sectional | 76 | 33/43 | 68.37±9.29 | MMSE, CDR, CMS | 59 | 77.63% |
| Zhuo 2022 | 2019.02-2020.02 | East China/Fujian | Hospital-based | Cross-sectional | 101 | 85/16 | 52 | MoCA, MMSE | 33 | 32.67% |
| Zou 2021 | 2018-2020 | Nationwide | Community-based | Cohort | 5414 | 2479/2935 | 73.4±6.6 | MMSE | 1405 | 25.95% |

**The list of included studies**

1. Cai SL, Liu YF, Chen CX, Han XH. Analysis on the Influencing Factors of Elderly Hypertensive Patients with Cognitive Dysfunction. *Clinical Medicine of China* (2018) 34(3):228-32. Epub 20181105. doi: 10.3760/cma.j.issn.1008-6315.2018.03.007.

2. Chen ZZ, Jiang FF, Yang M. Influencing Factors of Cognitive Dysfunction in Hypertension Patients with Lacunar Cerebral Infarction. *Chin J Geriatr Heart Brain Vessel Dis* (2022) 24(04):408-11. doi: 10.3969/j.issn.1009-0126.2022.04.019.

3. Cheng K, Yue J, Zhang RH, Guo XW, Yang L, Liu YY, et al. Correlation between Cardiovascular Risk Stratification and Cognitive Impairment in Elderly Hypertensive Patients. *Chin J Geriatr Heart Brain Vessel Dis* (2014) 16(04):380-3. doi: 10.3969/j.issn.1009-0126.2014.04.013.

4. Cheng YP. (2011) The Research of the Risk Factors for Vascular Cognitive Impairment in the Inpatients with Hypertension between Different Stages [dissertation/master's thesis].[China]: Dalian Medical University.

5. Chi LY, Hu XQ, Hu J, Bai T, Zhang YH, Tian HY. Correlation between Urinary Albumin/Creatinine Ratio and Cognitive Function in Patients with Hypertension. *Chin J Cardiovasc Med* (2018) 23(1):47-50. Epub 20181105. doi: 10.3969/j.issn.1007-5410.2018.01.011.

6. Feng P. (2018) Neuropsychology and Neurology of Hypertensive Patients with Mci Preliminary Study of Chinese Medical Evidence [dissertation/master's thesis].[China]: Beijing university of chinese medicine.

7. Gan XG, Liu D, Cheng GR, Zhang B, An LN, Wu Y, L., et al. The Association Analysis of Concurrent Diabetes and Hypertension with Mild Cognitive Impairment in Community Dwelling Older Adults. *Modern Preventive Medicine* (2021) 48(07):1202-5+14.

8. Gao YL, Kang L, Zhang Y. Correlation between Blood Pressure Variability, Circadian Arterial Blood Pressure, Pulse Pressure Index and Cognitive Impairment in Elderly Patients with Hypertension. *Journal of Clinical Medicine in Practice* (2020) 24(3):56-60. Epub 20210210. doi: 10.7619/jcmp.202003016.

9. Ge GZ, Tang Z, Sun F, Wu XG, Diao LJ, He HJ. Study on the Relationship between Mild Cognitive Impairment and Chronic Disease in Aged People of Beijing Community. *Chin J Geriatr Heart Brain Vessel Dis* (2009) (7):518-20. Epub 20110320. doi: 10.3969/j.issn.1009-0126.2009.07.012.

10. Geng SS, Jiang H, Ge JL, Li QQ, Niu XP, Liu Y. Value of Vitamin D3 for the Identification of Cognitive Impairment in Elderly Patients with Hypertension. *Chinese Journal Practice* (2018) 21(34):4222-6. Epub 20190520. doi: 10.12114/j.issn.1007-9572.2018.34.012.

11. Gong QY, Wang XL. Study on Relationship among H-Type Hypertension ,Carotid Artery Intima Mediathickness and Cognitive Impairmentin Elderly People. *Medicine and Philosophy(B)* (2017) 38(03):29-31. doi: 10.12014/j.issn.1002-0772.2017.03b.09.

12. Gu YC. (2019) The Study About the Cerebral Default Mode Network and Cortical Morphology Alteration of Hypertension Related Cognitive Impairment [dissertation/master's thesis].[China]: Southeast University.

13. He XJ, Xu ZH. The Relationships between Cognitive Impairment and Levels of Hcy, Hif - 1alpha and Ccl2 in Patients with Hypertension. *Labeled Immunoassays ＆ Clin Med* (2020) 27(02):271-4. doi: 10.11748/bjmy.issn.1006-1703.2020.02.019.

14. He ZL. (2020) Effects of Sleep Quality on Cognitive Impairment in the Elderly Patients with Hypertension [dissertation/master's thesis].[China]: Anhui Medical University.

15. Heizhati M, Li N, Wang L, Hong J, Li M, Yang W, et al. Association of Hypertension with Mild Cognitive Impairment in Population from Less-Developed Areas of Multiethnic Northwest China. *Neuroepidemiology* (2021) 55(5):407-15. Epub 2021/09/14. doi: 10.1159/000517956.

16. Hu R, Wang ZF, lv LZ, Wang L, Wang XY, Wang YL. Effects of Hypertensive Disorders on Cognitive Function in Community-Dwelling Older Adults. *Clinical Focus* (2013) 28(09):1018-20. doi: 10.3969/j.issn.1004-583X.2013.09.019.

17. Huang CQ, Dong BR, Zhang YL, Wu HM, Liu QX, Flaherty JH. Cognitive Impairment and Hypertension among Chinese Nonagenarians and Centenarians. *Hypertension research : official journal of the Japanese Society of Hypertension* (2009) 32(7):554-8. Epub 2009/05/30. doi: 10.1038/hr.2009.72.

18. Jin DA, Ye ZH. Effects of Hypertension and Diabetes on Cognitive Function in the Elderly. *Chin J Hypertens* (2018) 26(09):875-8. doi: 10.16439/j.cnki.1673-7245.2018.09.025.

19. Jin X. (2010) Relationship between Cognition and Microalbuminuria in Elderly Patients with Primary Hypertension [dissertation/master's thesis].[China]: Hebei Medical University.

20. Ke TX. Cognitive Function Changes in Elderly Hypertensive Patients in Community. *China &Foreign Medical Treatment* (2018) 37(35):69-71. doi: 10.16662/j.cnki.1674-0742.2018.35.069.

21. Li GD, Zhu F, Liu y. Cognitive Dysfunction in Elderly Hypertensive Patients and Postural Correlation between the Occurrence of Blood Pressure. *China Higher Medical Education* (2020) (8):135+47. Epub 20210603. doi: 10.3969/j.issn.1002-1701.2020.08.071.

22. Li H. (2021) Study on the Clinical Characteristics and Related Factors of Cognitive Dysfunction in Elderly Patients with Hypertension [dissertation/master's thesis].[China]: Shanxi Medical University.

23. Li HW, Zhao H, Li HT, Liu PG. Association between Serum Dehydroepiandrosterone Sulfate and Cognition in Elderly Male Patients with Hypertension. *Modern Preventive Medicine* (2016) 43(16):3061-4.

24. Li J, Sun JX, Ding LF, Wang Y, Guo HM, Wang XM. Effects of Homocysteine on the Cognitive Function in Elderly Patients with Hypertension. *Pract Geriatr* (2017) 31(6):568-71. Epub 20171231. doi: 10.3969/j.issn.1003-9198.2017.06.020.

25. Li Q. (2021) Correlation between the Level of Serum Uric Acid and Mild Cognitive Impairment in Patients with H-Type Hypertension [dissertation/master's thesis].[China]: Xinxiang Medical University.

26. Li T, Bai Y, Xiang J, Wang R, Bai J, Tuo X, et al. Duration of Hypertension Is Associated with Cognitive Function: A Cross-Sectional Study in Chinese Adults. *Chinese medical journal* (2014) 127(11):2105-10. Epub 2014/06/04.

27. Li Y, Zeng L, Sun Y, Lu YR, Tang YF. Relationship Study between Inflammatory Factors Il-6, Il-10, Hs-Crp and Cognitive Dysfunction in Elderly Patients with Hypertension. *Sichuan Medical Journal* (2020) 41(07):676-9. doi: 10.16252/j.cnki.issn1004-0501-2020.07.002.

28. Li YM, Wan QL, He RL, Cheng GC. Hypertension Combined with Cognitive Dysfunction Blood Pressure Variability in Patients. *Chin J Hypertens* (2019) 27(06):559-61. doi: 10.16439/j.cnki.1673-7245.2019.06.014.

29. Li ZQ, Wang SS, Gong XR, Wang YD, Wu D, Yang MT, et al. Analysis on Prevalence and Risk Factors of Cognitive Frailty in Beijing Rural Elder Hypertensive Patients. *Chin J Prev Contr Chron Dis* (2022) 30(12):915-9. doi: 10.16386/j.cjpccd.issn.1004-6194.2022.12.007.

30. Liang X, Chen YJ, Bi XR, Li CH, Zhou Q, Xu M, et al. Influencing Factors of Cognitive Impairment among the Community Elderly Patients with Hypertension in Shanghai. *Journal of Neuroscience and Mental Health* (2021) 21(9):613-7. Epub 20220523. doi: 10.3969/j.issn.1009-6574.2021.09.002.

31. Lin M. (2015) The Relationship between Parameters of Ambulatory Blood Pressure Monitoring and Cognitive Impairment in Elderly Hypertensive Patients [dissertation/master's thesis].[China]: Fujian Medical University.

32. Lin XD. Expression Levels and Correlations of Inflammatory Factors in Cognitive Dysfunction in Elderly Hypertensive Patients. *Chinese Journal of Geriatric Care* (2022) 20(2):89-91. Epub 20230120. doi: 10.3969/j.issn.1672-2671.2022.02.024.

33. Liu H, Fu R, Wang HY, Sun HY. The Correlation between Serum Hcy Expression, Blood Pressure Fluctuation and Mild Cognitive Impairment in Elderly Patients with Essential Hypertension. *Journal of Logistics University of PAP(Medical Sciences)* (2021) 30(05):92-5. doi: 10.16548/j.2095-3720.2021.05.029.

34. Liu T. (2018) The Analysis of Risk Factors of Primary Hypertensive and Lacunar Infarction Patients with Cognitive Impairment [dissertation/master's thesis].[China]: Xinjiang Medical University.

35. Liu YD, Pu ZY, Zang Z, X., Wei YF. A Study on the Cognitive Function and Psychological Status of Patients with H Hypertension. *China health industry* (2014) 11(16):116-7. Epub 20141228.

36. Luo X. (2022) Association between Serum Uric Acid Levels and Cognitive Dysfunction in Elderly Patients with Hypertension [dissertation/master's thesis].[China]: Nanchang University.

37. Luo ZX. Correlation between Hypertension and Cognitive Impairment in Elderly People. *Journal of Molecular Imaging* (2015) 38(03):293-5. doi: 10.3969/j.issn.1674-4500.2015.03.39.

38. Ma J, Zhang J, Liu JL, Mei L, Wang SW, Zhang HM, et al. Serum Aβ1-42 Amyloid and Tau Protein Levels and Hypertension in Elderly Patients in Highland Areas the Correlation between Serum Aβ1-42 Amyloid and Tau Protein Levels and the Occurrence of Mild Cognitive Impairment in Elderly Patients with Hypertension in Highland Areas. *Journal of High Altitude Medicine* (2020) 30(03):1-5. doi: 10.3969/j.issn.1007-3809.2020.03.001.

39. Ma JY. (2021) The Study on the Correlation between White Matter Hyperintensity, Disrupted White Matter Microstructure and Hypertension Related Cognitive Impairment [dissertation/master's thesis].[China]: Southeastern University.

40. Ma LY, He F, Liu S, Wang XD, Gao Y, Shi Z, et al. The Association between the Prevalence, Medication Adherence and Control of Hypertension and the Prevalence of Mild Cognitive Impairment in Rural Northern China: A Cross-Sectional Study. *Patient preference and adherence* (2022) 16:493-502. Epub 2022/03/02. doi: 10.2147/ppa.S351588.

41. Ma WR, Li MN, Wang LQ, Wang ZZ. Risk Factors of Hypertension Combined with Mild Cognitive Impairment in Community Population over 55 Years. *Chinese Journal Practice* (2022) 26:1075-9. doi: 10.12114/j.issn.1007-9572.2022.0733.

42. Ma XX, Wang L, Cheng YF, Yang YH, Gong Y, Yang H, et al. Relationship between Cognitive Im Pairm Entand Serum Hbalc Levelin Elderly Hypertensive Patients. *Chin J Geriatr Heart Brain Vessel Dis* (2015) 17(07):689-92. doi: 10.3969/j.issn.1009-0126.2015.07.006.

43. Pan JX, Chen LQ, Wang JL, Xu HQ, Xie BQ. Cognitive Function Status and Influencing Factors of Hypertensive Elderly in Shanghai Community. *CHINESE PRIMARY HEALTH CARE* (2021) 35(10):36-9. Epub 20220523. doi: 10.3969/j.issn.1001-568X.2021.10.0011.

44. Peng Y, Luo XJ, Chen XP, Li LX, Wan LY, He S, et al. [Association of Ppargamma2 Pro12ala Polymorphism and Cognitive Dysfunction in Hypertension Patients]. *Sichuan da xue xue bao Yi xue ban = Journal of Sichuan University Medical science edition* (2010) 41(6):1034-8. Epub 2011/01/27. doi: 10.13464/j.scuxbyxb.2010.06.011.

45. Qi M, Wang XL. The Related Factors Analysis on Elderly Hypertension Patients with Cognitive Impairment Related Factors Analysis and the Influenceon Their Quality of Life. *Medicine and Philosophy(B)* (2015) 36(04):46-8.

46. Qin Z, Wu W, Liu D, Zheng C, Kang J, Zhou H, et al. Quantitative Susceptibility Mapping of Brain Iron Relating to Cognitive Impairment in Hypertension. *Journal of Magnetic Resonance Imaging* (2022) 56(2):508-15. doi: 10.1002/jmri.28043.

47. Qu L, Dong Z, Ma S, Liu Y, Zhou W, Wang Z, et al. Gut Microbiome Signatures Are Predictive of Cognitive Impairment in Hypertension Patients—a Cohort Study. *Frontiers in Microbiology* (2022) 13:841614. doi: 10.3389/fmicb.2022.841614.

48. Quan QY, Zhao XJ, Zhang Y, Wang JG, Li Q. Correlations between Serum Pro-Bnp and Hs-Crp Levels and Cognition in Elderly Patients with Hypertension. *Med J Chin PAP* (2022) 33(01):57-9. doi: 10.14010/j.cnki.wjyx.2022.01.011.

49. Shi XC. (2021) Correlation between Cognitive Impairment and Anxiety and Depression in Patients with Essential Hypertension [dissertation/master's thesis].[China]: Hebei Medical University.

50. Song TX, Wang M, Yuan L, Han D, Zhang Y, Zhou L, et al. Survey on the Hypertension and Cognitive Function of the Elderly at Old Folks'home in Chongqing. *Chinese Journal of Gerontology* (2014) (7):1899-901. Epub 20200908. doi: 10.3969/j.issn.1005-9202.2014.07.078.

51. Song YL, Lin J, Xia WJ, Xue B, Wu YP. Association of Homocysteine with Mild Cognitive Impairment in Hypertension Patients. *Chin J Stroke* (2020) 15(12):1322-6. Epub 20210629. doi: 10.3969/j.issn.1673-5765.2020.12.012.

52. Su YL, Chen XP, Huang Y, Jiang L, Huang H. [Relationship between Apolipoprotein E Polymorphism and Cognitive Function in Patients with Primary Hypertension]. *Journal of Biomedical Engineering* (2009) 26(4):856-60. Epub 2009/10/10.

53. Sun H. (2021) Effect of Hyperhomocyteinemia on Cognitive Function in Patients with Hypertension [dissertation/master's thesis].[China]: Inner mongolia medical university.

54. Wei XY, Jiang YG, Huang CY, Yang LR, Song YQ, Cheng DM. H-Type Hypertension Severity and Cognitive Function in Middle-Aged and Elderly Chinese People with Hypertension. *Chinese General Practice* (2021) 24(20):2520-6. Epub 20211116. doi: 10.12114/j.issn.1007-9572.2021.00.558.

55. Wu L, He Y, Jiang B, Liu M, Wang J, Yang S, et al. The Association between the Prevalence, Treatment and Control of Hypertension and the Risk of Mild Cognitive Impairment in an Elderly Urban Population in China. *Hypertension research : official journal of the Japanese Society of Hypertension* (2016) 39(5):367-75. Epub 2016/01/08. doi: 10.1038/hr.2015.146.

56. Wumaer., Aizezi. An Analysis on the Relationship between Cognitive Function and Microalbuminuria and Its Influence Factors in Elderly Patients with Hypertension. *Chinese Journal of Frontiers in Medicine* (2015) 7(10):90-3. Epub 20161024.

57. Xi M, Zhou R, Luo D, Yang W, Wang XQ, Jiang WH, et al. Analysis of the Characteristics and Associated Clinical Risk Factors of Hypertension with Mild Cognitive Dysfunction in the Elderly. *Chin J Clin Healthc* (2022) 25(1):70-4. Epub 20220921. doi: 10.3969/J.issn.1672-6790.2022.01.016.

58. Xu W, Ren JB, Xi B. Correlation between the Severity of Cognitive Impairment and Prognosis in Elderly Patients with Hypertension. *Hainan Med J* (2020) 31(01):23-6. doi: 10.3969/j.issn.1003-6350.2020.01.007.

59. Xu XY, Guo XC, Huang LM, Lai ZY, Liu KT. Analysis of the Cognitive Impairment Characteristics and Risk Factors in Hypertensive Patients with Vascular Mild Cognitive Impairment. *Chinese and Foreign Medical Research* (2018) 16(12):55-7. doi: 10.14033/j.cnki.cfmr.2018.12.025.

60. Xue Q, Song LQ, Tian W, Deng XH, Zhang P. Blood Pressure Variability and Cognitive Impairment in Very Elderly Hypertensive Patients. *Chin J Mult Organ Dis Elderly* (2018) 17(06):407-11. doi: 10.11915/j.issn.1671-5403.2018.06.091.

61. Xue QD. (2020) Correlation between Sleep Quality and Cognitive Function in Patients with Hypertension [dissertation/master's thesis].[China]: China Medical University.

62. Xue YH, Chen XJ. Relationship between Ambulatory Blood Pressure Parameters and Cognitive Function in Elderly Patients with Hypertension. *Chinese Practical Journal of Rural Doctor* (2019) 26(7):52-4. Epub 20200125. doi: 10.3969/j.issn.1672-7185.2019.07.020.

63. Yan X, Meng T, Liu H, Liu J, Du J, Chang C. The Association between the Duration, Treatment, Control of Hypertension and Lifestyle Risk Factors in Middle-Aged and Elderly Patients with Mild Cognitive Impairment: A Case-Control Study. *Neuropsychiatric disease and treatment* (2022) 18:585-95. Epub 2022/03/29. doi: 10.2147/ndt.S353164.

64. Yang CH, Cai W, Pang XF, Gong Y. Relationship between Dynamic Blood Pressure, Plasma Homocysteine Level and Cognitive Dysfunction in Elderly Patients with Hypertension. *Practical Journal of Cardiac Cerebral Pneumal and Vascular Disease* (2018) 26(S2):36-8.

65. Yang YQ, Qi Y, Liu S, Chang J, Hu DQ, Sun ZQ, et al. Association between Blood Pressure and the Prevalence of Mild Cognitive Impairment in Elderly Population. *Journal of Cardiovascular & Pulmonary Diseases* (2022) 41(3):215-20. Epub 20230120. doi: 10.3969/j.issn.1007-5062.2022.03.001.

66. Ye QF, Wang YN, Li L, Liu GJ, Lin P, Li QJ. Prevalence and Associated Factors of Mild Cognitive Impairment in Young and Middle-Aged Hospitalized Patients with Hypertension. *Chinese Journal Practice* (2023) 26(2):154-9,67. Epub 20230120. doi: 10.12114/j.issn.1007-9572.2022.0576.

67. Yuan F, Liu S, Liang XN, Fu SH, Ding D, Luo JF. Relationship between Hypertension and Incidence Risk of Dementia in Community Elderly. *Chin J Clin Neurosci* (2021) 29(5):519-26. Epub 20220523.

68. Zhang HY, Meng YL, Zhang JY, Li GR, Zhang M. Blood Pressure and Cognitive Function Status of the Elderly in Tangshan. *Chinese Journal of Gerontology* (2014) 34(5):1348-9. Epub 20200908. doi: 10.3969/j.issn.1005-9202.2014.05.087.

69. Zhang JN, Chen CX, Li JM. Hypertension, Diabetes Mellitus and the Cognitive Disorder in Cerebrovascular Patients: 915 Cases Investigate. *Chin J Rehabil Theory Pract* (2008) 14(3):251-2. Epub 20080530. doi: 10.3969/j.issn.1006-9771.2008.03.020.

70. Zhang JN, Chen CX, Li JM, Wen YL. An Investigation of the Cognitive Disorder of Cerebrovascular Disease Patients and the Related Factors. *Journal of Brain and Nervous Diseases* (2008) 16(01):14-6. doi: 10.3969/j.issn.1006-351X.2008.01.005.

71. Zhang L. (2011) The Incidence and Risk Factors of Vascular Cognitive Impairment in Inpatients with Hypertension [dissertation/master's thesis].[China]: Dalian Medical University.

72. Zhang T. (2020) Cognitive Impairment and Risk Factors in High Altitude Hypertension [dissertation/master's thesis].[China]: Ningxia Medical University.

73. Zhang W, Guo Y, Zhou XQ, Liu XH, Yan YQ. Effect of Leisure Physical Activities on Cognitive Function of Elderly Patients with Hypertension in Community. *Chinese Journal Practice* (2022) 25(22):2720-5. Epub 20230120. doi: 10.12114/j.issn.1007-9572.2022.0221.

74. Zhang W, Yan YQ, Liu XH, Zhou XQ, Guo Y. The Impact of Hypertension,Diabetes, Hyperlipidemia and the Comorbidities on Cognitive Function in the Elderly. *Chin Prev Med* (2021) 22(6):411-7. Epub 20220921. doi: 10.16506/j.1009-6639.2021.06.003.

75. Zhao XJ, Quan QY, Zhang Y. Correlation between Serum Cystatin-C Level and Congnitive Impairment in Elderly Patients with Hypertension. *J Mod Lab Med* (2020) 35(01):136-8. doi: 10.3969/j.issn.1671-7414.2020.01.036.

76. Zhao XL, Wang M. Analysis Influence of Hypertension, Diabetes on Cognitive Function in Patients with Cerebrovascular Disease. *China Continuing Medical Education* (2016) 8(6):129-30. Epub 20161024. doi: 10.3969/j.issn.1674-9308.2016.06.098.

77. Zhao YW, Wu G, Shi HM, Xia Z, Sun T. Relationship between Cognitive Impairment and Apparent Diffusion Coefficient Values from Magnetic Resonance-Diffusion Weighted Imaging in Elderly Hypertensive Patients. *Clinical interventions in aging* (2014) 9:1223-31. Epub 2014/08/13. doi: 10.2147/cia.S63567.

78. Zheng JL. Correlation between Homocysteine Levels and Cognitive Performance in Patients with H Hypertension. *Health for Everyone* (2021) 543(10):60-1. doi: 10.3969/j.issn.1004-597X.2021.10.035.

79. Zhou D, Zhou L, Hu XY. Correlation Degree of Cognitive Dysfunction and Inflammation in Patients with H Hypertension and Oxidative Stress Factor Levels *Chin J Hypertens* (2018) 26(09):871-4. doi: 10.16439/j.cnki.1673-7245.2018.09.024.

80. Zhou T. (2011) Preliminary Study of Chinese Medical Evidence in Patients with Hypertension with Mild Cognitive Impairment [dissertation/master's thesis].[China]: Beijing University of Chinese Medicine.

81. Zhuo X, Huang M, Wu M, Elshmaa M. Analysis of Cognitive Dysfunction and Its Risk Factors in Patients with Hypertension. *Medicine (United States)* (2022) 101(10):E28934. doi: 10.1097/MD.0000000000028934.

82. Zou MJ, Huang JW, Zeng Y, Cheng GR, Chen C, Li L, et al. Association between Systolic Blood Pressure Control and Cognitive Function in Community-Living Older Adults 65 Years and Older with Hypertension. *Chinese Journal of Disease Control and Prevention* (2021) 25(11):1282-92. doi: 10.16462/j.cnki.zhjbkz.2021.11.008.

**Supplementary Table 5. The risk of bias of the included studies**

| Study | 1 | 2 | 3 | 4 | 5 | 6 | 7 | 8 | 9 | 10 | Overall risk |
| --- | --- | --- | --- | --- | --- | --- | --- | --- | --- | --- | --- |
| Cai 2018 | High | High | Low | Low | Low | Low | Low | Low | Low | Low | Moderate |
| Chen 2022 | High | High | High | Low | Low | Low | Low | Low | Low | Low | Moderate |
| Cheng 2014 | High | High | High | Low | Low | Low | Low | Low | Low | Low | Moderate |
| Cheng 2011 | High | High | High | Low | Low | Low | Low | Low | Low | Low | Moderate |
| Chi 2018 | High | High | High | Low | Low | Low | Low | Low | Low | Low | Moderate |
| Feng 2018 | High | High | High | Low | Low | Low | Low | Low | Low | Low | Moderate |
| Gan 2021 | High | Low | Low | Low | Low | Low | Low | Low | Low | Low | Low |
| Gao 2020 | High | High | High | Low | Low | Low | Low | Low | Low | Low | Moderate |
| Ge 2009 | High | Low | Low | Low | Low | Low | Low | Low | Low | Low | Low |
| Geng 2018 | High | High | High | Low | Low | Low | Low | Low | Low | Low | Moderate |
| Gong 2017 | High | High | High | Low | Low | Low | Low | Low | Low | Low | Moderate |
| Gu 2019 | High | High | High | Low | Low | Low | Low | Low | Low | Low | Moderate |
| He 2020 | High | High | High | Low | Low | Low | Low | Low | Low | Low | Moderate |
| He 2020 | High | High | High | Low | Low | Low | Low | Low | Low | Low | Moderate |
| Heizhati 2021 | Low | Low | High | Low | Low | Low | Low | Low | Low | Low | Low |
| Hu 2013 | High | High | High | Low | Low | Low | Low | Low | Low | Low | Moderate |
| Huang 2009 | High | High | High | Low | Low | Low | Low | Low | Low | Low | Moderate |
| Jin 2018 | High | High | High | Low | Low | Low | Low | Low | Low | Low | Moderate |
| Jin 2010 | High | High | High | Low | Low | Low | Low | Low | Low | Low | Moderate |
| Ke 2018 | High | High | High | Low | Low | Low | Low | Low | Low | Low | Moderate |
| Li 2020 | High | High | High | Low | Low | Low | Low | Low | Low | Low | Moderate |
| Li 2021 | High | High | High | Low | Low | Low | Low | Low | Low | Low | Moderate |
| Li 2016 | High | High | High | Low | Low | Low | Low | Low | Low | Low | Moderate |
| Li 2017 | High | High | High | Low | Low | Low | Low | Low | Low | Low | Moderate |
| Li 2021 | High | High | High | Low | Low | Low | Low | Low | Low | Low | Moderate |
| Li 2014 | High | High | High | Low | Low | Low | Low | Low | Low | Low | Moderate |
| Li 2020 | High | High | High | Low | Low | Low | Low | Low | Low | Low | Moderate |
| Li 2019 | High | High | High | Low | Low | Low | Low | Low | Low | Low | Moderate |
| Li 2022 | High | Low | Low | Low | Low | Low | Low | Low | Low | Low | Low |
| Liang 2021 | High | High | High | Low | Low | Low | Low | Low | Low | Low | Moderate |
| Lin 2015 | High | High | High | Low | Low | Low | Low | Low | Low | Low | Moderate |
| Lin 2022 | High | High | High | Low | Low | Low | Low | Low | Low | Low | Moderate |
| Liu 2021 | High | High | High | Low | Low | Low | Low | Low | Low | Low | Moderate |
| Liu 2018 | High | High | High | Low | Low | Low | Low | Low | Low | Low | Moderate |
| Liu 2014 | High | High | High | Low | Low | Low | Low | Low | Low | Low | Moderate |
| Luo 2022 | Low | Low | Low | Low | High | Low | Low | Low | Low | Low | Low |
| Luo 2015 | High | High | High | Low | Low | Low | Low | Low | Low | Low | Moderate |
| Ma 2020 | High | High | High | Low | Low | Low | Low | Low | Low | Low | Moderate |
| Ma 2021 | High | High | High | Low | Low | Low | Low | Low | Low | Low | Moderate |
| Ma 2022 | High | Low | Low | Low | Low | Low | Low | Low | Low | Low | Low |
| Ma 2022 | High | Low | Low | Low | Low | Low | Low | Low | Low | Low | Low |
| Ma 2015 | High | High | High | Low | Low | Low | Low | Low | Low | Low | Moderate |
| Pan 2021 | High | High | High | Low | Low | Low | Low | Low | Low | Low | Moderate |
| Peng 2010 | High | High | High | Low | Low | Low | Low | Low | Low | Low | Moderate |
| Qi 2015 | High | High | High | Low | Low | Low | Low | Low | Low | Low | Moderate |
| Qin 2022 | High | High | High | Low | Low | Low | Low | Low | Low | Low | Moderate |
| Qu 2022 | High | High | High | Low | Low | Low | Low | Low | Low | Low | Moderate |
| Quan 2022 | High | High | High | Low | Low | Low | Low | Low | Low | Low | Moderate |
| Shi 2021 | High | High | High | Low | Low | Low | Low | Low | Low | Low | Moderate |
| Song 2014 | High | Low | Low | Low | Low | Low | Low | Low | Low | Low | Low |
| Song 2020 | High | High | High | Low | Low | Low | Low | Low | Low | Low | Moderate |
| Su 2009 | High | High | High | Low | Low | Low | Low | Low | Low | Low | Moderate |
| Sun 2021 | High | High | High | Low | Low | Low | Low | Low | Low | Low | Moderate |
| Wei 2021 | High | High | High | Low | Low | Low | Low | Low | Low | Low | Moderate |
| Wu 2016 | High | Low | Low | Low | Low | Low | Low | Low | Low | Low | Low |
| Wumaer 2015 | High | High | High | Low | Low | Low | Low | Low | Low | Low | Moderate |
| Xi 2022 | High | High | High | Low | Low | Low | Low | Low | Low | Low | Moderate |
| Xu 2020 | High | High | High | Low | Low | Low | Low | Low | Low | Low | Moderate |
| Xu 2018 | High | High | High | Low | Low | Low | Low | Low | Low | Low | Moderate |
| Xue 2018 | High | High | High | Low | Low | Low | Low | Low | Low | Low | Moderate |
| Xue 2020 | High | High | High | Low | Low | Low | Low | Low | Low | Low | Moderate |
| Xue 2019 | High | High | High | Low | Low | Low | Low | Low | Low | Low | Moderate |
| Yan 2022 | High | High | High | Low | Low | Low | Low | Low | Low | Low | Moderate |
| Yang 2018 | High | High | High | Low | Low | Low | Low | Low | Low | Low | Moderate |
| Yang 2022 | High | Low | Low | Low | Low | Low | Low | Low | Low | Low | Low |
| Ye 2023 | High | High | High | Low | Low | Low | Low | Low | Low | Low | Moderate |
| Yuan 2021 | High | High | High | Low | Low | Low | Low | Low | Low | Low | Moderate |
| Zhang 2014 | High | High | High | Low | Low | Low | Low | Low | Low | Low | Moderate |
| Zhang 2008 | Low | Low | Low | Low | Low | Low | Low | Low | Low | Low | Low |
| Zhang 2008 | High | High | High | Low | Low | Low | Low | Low | Low | Low | Moderate |
| Zhang 2011 | High | High | High | Low | Low | Low | Low | Low | Low | Low | Moderate |
| Zhang 2020 | High | High | High | Low | Low | Low | Low | Low | Low | Low | Moderate |
| Zhang 2022 | High | Low | Low | Low | Low | Low | Low | Low | Low | Low | Low |
| Zhang 2021 | High | Low | Low | Low | Low | Low | Low | Low | Low | Low | Low |
| Zhao 2020 | High | High | High | Low | Low | Low | Low | Low | Low | Low | Moderate |
| Zhao 2016 | High | High | High | Low | Low | Low | Low | Low | Low | Low | Moderate |
| Zhao 2014 | High | High | High | Low | Low | Low | Low | Low | Low | Low | Moderate |
| Zheng 2021 | High | High | High | Low | Low | Low | Low | Low | Low | Low | Moderate |
| Zhou 2018 | High | High | High | Low | Low | Low | Low | Low | Low | Low | Moderate |
| Zhou 2011 | High | Low | Low | Low | Low | Low | Low | Low | Low | Low | Low |
| Zhuo 2022 | High | High | High | Low | Low | Low | Low | Low | Low | Low | Moderate |
| Zou 2021 | Low | Low | Low | Low | Low | Low | Low | Low | Low | Low | Low |

Note:1= representation of target population; 2= representation of sampling frame; 3= random selection of sample; 4= minimal non-response bias; 5= data collected directly from subjects; 6= suitable case definition; 7= validated measurement; 8= consistent mode of data collection; 9= suitable length of the shortest prevalence period; 10= right calculation of prevalence;

Low: low risk; Moderate: Moderate risk; High: high risk

**Supplementary Table 6. The results of the univariate meta-regression**

| **univariable** | **estimate** | **Standard**  **error** | **Z** | ***P*** | **95%CI** |
| --- | --- | --- | --- | --- | --- |
| **Survey period** |  |  |  |  |  |
| before 2010 | reference | / | / | / | / |
| 2010-2015 | -0.1845 | 0.3048 | -0.6053 | 0.5449 | -0.7819, 0.4129 |
| 2015-2020 | 0.0484 | 0.2792 | 0.1734 | 0.8624 | -0.4988, 0.5957 |
| after 2020 | 0.0123 | 0.4991 | 0.0245 | 0.9804 | -0.966, 0.9905 |
| **Region** |  |  |  |  |  |
| Nationwide | reference | / | / | / | / |
| Central China | 0.0981 | 0.5598 | 0.1752 | 0.8609 | -0.9991, 1.1953 |
| East China | 0.3807 | 0.51 | 0.7463 | 0.4555 | -0.619, 1.3803 |
| North China | -0.0526 | 0.5097 | -0.1031 | 0.9179 | -1.0515, 0.9464 |
| Northeast China | 0.8083 | 0.5714 | 1.4146 | 0.1572 | -0.3116, 1.9282 |
| Northwest China | -0.0812 | 0.5293 | -0.1534 | 0.8781 | -1.1187, 0.9563 |
| South China | -0.6705 | 0.9597 | -0.6987 | 0.4847 | -2.5514, 1.2104 |
| Southwest China | 0.214 | 0.5601 | 0.382 | 0.7025 | -0.8838, 1.3118 |
| **Recruitment Source** |  |  |  |  |  |
| Community-based | reference | / | / | / | / |
| Hospital-based | 0.6372 | 0.2221 | 2.8683 | 0.0041 | 0.2018, 1.0726 |
| **Study design** |  |  |  |  |  |
| Cohort | reference | / | / | / | / |
| Cross-sectional | 0.6805 | 0.2331 | 2.9191 | 0.0035 | 0.2236, 1.1375 |
| **Assessment tools** |  |  |  |  |  |
| AD-8 | reference | / | / | / | / |
| CDR, DSV | -1.1866 | 0.9523 | -1.2461 | 0.2127 | -3.0531, 0.6799 |
| MMSE | 0.7513 | 0.6833 | 1.0996 | 0.2715 | -0.5879, 2.0906 |
| MMSE, BCAT | -0.0644 | 0.9859 | -0.0653 | 0.9479 | -1.9966, 1.8679 |
| MMSE, CDR, CMS | 2.7668 | 0.9882 | 2.8 | 0.0051 | 0.8301, 4.7036 |
| MMSE, CDT | 1.3161 | 0.9498 | 1.3857 | 0.1658 | -0.5455, 3.1776 |
| MMSE, DSV-Ⅳ | 0.1342 | 0.826 | 0.1625 | 0.8709 | -1.4847, 1.7531 |
| MMSE, MoCA | 1.4499 | 0.709 | 2.0451 | 0.0408 | 0.0603, 2.8394 |
| MMSE, MoCA, Peterson | 0.2801 | 0.9825 | 0.2851 | 0.7756 | -1.6455, 2.2057 |
| MMSE, Peterson | -0.004 | 0.9543 | -0.0042 | 0.9966 | -1.8744, 1.8663 |
| MoCA | 1.4522 | 0.6977 | 2.0812 | 0.0374 | 0.0846, 2.8197 |
| MoCA, CDR | 2.946 | 0.9681 | 3.0432 | 0.0023 | 1.0487, 4.8434 |
| MoCA, MMSE | 1.3846 | 0.7429 | 1.8638 | 0.0624 | -0.0715, 2.8408 |
| Peterson, CDR | 1.8049 | 0.9615 | 1.877 | 0.0605 | -0.0797, 3.6894 |
| **Sample size** |  |  |  |  |  |
| 0-100 | reference | / | / | / | / |
| 100-500 | 0.2898 | 0.3171 | 0.9139 | 0.3608 | -0.3317, 0.9112 |
| 500-1000 | -0.3493 | 0.3812 | -0.9163 | 0.3595 | -1.0964, 0.3978 |
| 1000 | -0.7983 | 0.4078 | -1.9578 | 0.0503 | -1.5975, 0.0009 |
| **Risk of bias** |  |  |  |  |  |
| Low | reference | / | / | / | / |
| Moderate | 0.4123 | 0.2466 | 1.6721 | 0.0945 | -0.071, 0.8955 |

**Supplementary Table 7. The** **certainty for the prevalence of cognitive impairment in Chinese hypertensive patients**

|  | Quality assessment | | | | | | | Summary of findings | | | | |
| --- | --- | --- | --- | --- | --- | --- | --- | --- | --- | --- | --- | --- |
|  |  |  |  |  |  |  |  | No. of patients | | Effect indicator | |  |
| outcomes | No. of studies | Limitations | Inconsistency | Indirectness | Imprecision | Publication bias | Other  considerations | With CI | Without CI | Effect size | 95%CI | Quality |
| **Prevalence of cognitive impairment in Chinese hypertensive patients** | 82 | No serious  limitations | No serious  inconsistency | No serious  indirectness | No serious  imprecision | Detected | Large effect | 15867 | 37756 | 37.6% | (33.2%, 42.2%) | ⊕⊕○○  Low |
